# Oncogenic enhancers drive esophageal squamous cell carcinogenesis and metastasis

**DOI:** 10.1038/s41467-021-24813-2

**Published:** 2021-07-22

**Authors:** Bo Ye, Dandan Fan, Weiwei Xiong, Min Li, Jian Yuan, Qi Jiang, Yuting Zhao, Jianxiang Lin, Jie Liu, Yilv Lv, Xiongjun Wang, Zhigang Li, Jianzhong Su, Yunbo Qiao

**Affiliations:** 1 Department of Thoracic Surgery, Shanghai Chest Hospital, Shanghai Jiaotong University, Shanghai, China; 2 Precise Genome Engineering Center, School of Life Sciences, Guangzhou University, Guangzhou, China; 3 School of Biomedical Engineering, School of Ophthalmology and Optometry and Eye Hospital, Wenzhou Medical University, Wenzhou, China; 4 Guangzhou University & Zhongshan People’s Hospital Joint Biomedical Institute, Guangzhou, China; 5 Wenzhou Institute, University of Chinese Academy of Sciences, Wenzhou, China

**Keywords:** Cancer epigenetics, Oesophageal cancer, Epigenomics

## Abstract

The role of *cis*-elements and their aberrations remains unclear in esophageal squamous cell carcinoma (ESCC, further abbreviated EC). Here we survey 28 H3K27ac-marked active enhancer profiles and 50 transcriptomes in primary EC, metastatic lymph node cancer (LNC), and adjacent normal (Nor) esophageal tissues. Thousands of gained or lost enhancers and hundreds of altered putative super-enhancers are identified in EC and LNC samples respectively relative to Nor, with a large number of common gained or lost enhancers. Moreover, these differential enhancers contribute to the transcriptomic aberrations in ECs and LNCs. We also reveal putative driver onco-transcription factors, depletion of which diminishes cell proliferation and migration. The administration of chemical inhibitors to suppress the predicted targets of gained super-enhances reveals HSP90AA1 and PDE4B as potential therapeutic targets for ESCC. Thus, our epigenomic profiling reveals a compendium of reprogrammed *cis*-regulatory elements during ESCC carcinogenesis and metastasis for uncovering promising targets for cancer treatment.

## Abstract (web summary)

The role of regulatory cis-elements in carcinogenesis and metastasis in esophageal squamous cell carcinoma remains crucial. Here the authors investigate H3K27ac-marked active enhancer profiles and transcriptomes in different types of esophageal tissues and identify oncogenic events and potential therapeutic targets.

## Introduction

Esophageal cancer has two main subtypes, esophageal squamous cell carcinoma (ESCC, further abbreviated EC) and esophageal adenocarcinoma (EAC)^1^. It is associated with a poor prognosis and a 5‐year overall survival rate of 18% only^2^. ESCC accounts for ~90% of esophageal cancer cases worldwide and predominates in eastern Asia and Africa^3^. Although the 5-year survival rate of patients with esophageal cancer has improved in the past decade, this cancer still has a very poor prognosis because patients generally do not show clinical features before an advanced stage unless via screening. Stage III tumors invade through the muscular layer and involve lymph nodes or other adjacent structures^3^. The 5-year survival rate after esophagectomy can benefit from lymph node removal, and the depth of invasion and presence of node metastasis are important predictors^4^. Thus, a better understanding of molecular dependencies and vulnerabilities during esophageal carcinogenesis and its metastasis to lymph nodes is urgently needed to develop promising diagnostic and therapeutic strategies.

An aberrant and reprogrammed transcriptome is a universal hallmark of human cancers that is associated with deregulated cell proliferation, invasion, and metastasis^5^. The leading causes of aberrant transcriptional patterns are genetic alterations and the deregulated signaling pathways and epigenomic traits, including DNA methylation, histone modification, and other *cis*-regulatory elements^6^. Multiple studies have highlighted the importance of histone modifications in the establishment and maintenance of disease and cancer statuses^7,8^, and super-enhancers (SEs), comprising clustered enhancers, are considered to be the key controllers of different cell types and disease conditions^9^, associated with disease-specific genetic variants and mutations^8,10^. We and others recently proposed that key transcription factor (TF)-driven reprogramming of enhancers, which are marked by histone H3 lysine 27 acetylation (H3K27ac) and localized distal to promoters and transcription start sites (TSSs), promotes carcinogenesis and metastasis^11–13^.

Several familial genetic risk mutations in *MSR1*, *ASCC1*, *CTHRC1*, *CRTC1*, *BARX1*, *FOXP1, TP53*, *CDKN2A*, *SMAD4*, and *NFE2L2*, have been identified in Barrett’s esophagus and adenocarcinoma patients^14–18^. Meanwhile, some familial form of ESCC and a number of susceptibility loci in ESCC patients as well some genetic drivers (e.g., *TP53*) have been reported^16,17,19–22^, while the complexity of carcinogenesis might be more than just genetic alterations. In recent years, the epigenomic profiling of tumor-specific promoter, enhancer, and super-enhancer landscapes during carcinogenesis has been widely used to elucidate the molecular mechanisms underlying the dysregulated local and regional expression of oncogenic genes in several cancers, such as colon cancer, gastric cancer, renal cell carcinoma (RCC), and ependymoma^8,23–27^. Although global histone modifications have been linked to the evolution of distant metastasis during pancreatic and prostate cancer progression^28,29^, the genome-wide epigenomic landscape of ESCC tumorigenesis and metastasis is poorly understood.

In this work, to address the role of enhancer dynamics during ESCC progression and metastasis to lymph nodes, we characterize the enhancer and SE landscapes in primary ESCC and lymph node cancer (LNC) tissues as well as normal esophageal epithelium. We identify a large set of commonly altered enhancers and LNC-specific enhancers associated with regional gene expression that can be used for precise subclassification. By analyzing the *trans*-elements within altered enhancers, we discover several key TFs regulating esophageal cancer cell proliferation and migration. By performing an integrated analysis of SE-associated genes with drug interaction databases, we identify some promising oncotargets that are responsive to pharmacologic inhibition. These findings indicate that the identification of variant enhancers and SEs is of great help for illustrating core transcriptional regulatory circuitries and discovering therapeutic targets for esophageal and LNCs.

## Results

### H3K27ac profiling defines active regulatory elements of esophageal carcinogenesis and metastasis

**Fig. 1:**
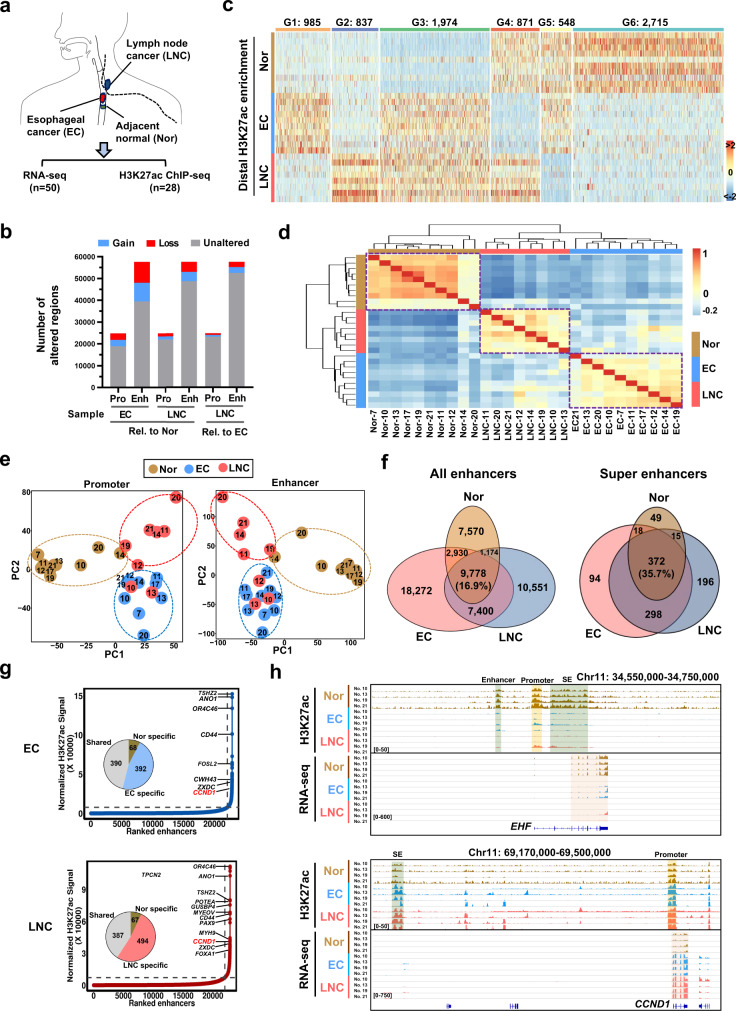
H3K27ac profiling defines active regulatory elements in primary esophageal and metastatic lymph node tumors. **a** Diagram of the experimental design for profiling the transcriptome and epigenome of adjacent normal tissues (Nor), esophageal squamous cell cancer (EC) tissues, and lymph node cancer (LNC) tissues. **b** Identification of the differentially distributed enhancers in Nor (*n* = 10), EC (*n* = 10), and LNC (*n* = 8) tissues. The numbers of altered (gained or lost) promoters (Pro) or enhancers (Enh) upon the comparison of Nor with EC or LNC, or EC with LNC are presented. Rel. relative. **c** Heatmap of the altered enhancers across Nor (*n* = 10), EC (*n* = 10), and LNC (*n* = 8) samples with H3K27ac enrichment signals. Six groups (G1–G6) of enhancers with H3K27ac enrichment signals with the indicated number of enhancer elements are presented. **d** Unsupervised hierarchical clustering analysis of 28 Nor, EC, and LNC samples’ differential enhancers shown in **c**. The number of patients number is also provided. **e** Principal component analysis of 28 Nor (*n* = 10), EC (*n* = 10), and LNC (*n* = 8) samples using all differentially distributed promoter elements shown in Supplementary Fig. 5a and enhancers shown in **c**. **f** Venn diagrams depicting the numbers of shared enhancers (left) and super-enhancers (right) across Nor, EC, and LNC samples. **g** Identification of super-enhancers in EC and LNC samples in infection plots. The top super-enhancer-associated genes are labeled. The number of shared, EC-specific, and LNC-specific super-enhancers across the three types of samples are displayed in pie charts. **h** Tracks of H3K27ac ChIP-seq and RNA-seq data at the *EHF* and *CCND1* loci in four representatives paired Nor, EC, and LNC samples. The previously identified enhancer^79^ upstream of the *CCND1* promoter is indicated. Source data are provided as a Source Data file.

To characterize active *cis*-regulatory elements in esophageal carcinogenesis and metastasis, we generated 28 active histone mark (H3K27ac) profiles from ten paired ESCC patients by chromatin immunoprecipitation and sequencing (ChIP-seq) with freshly dissected samples of adjacent normal esophageal (Nor or Adj) tissues, primary ESCC (further abbreviated EC herein), and LNC tissues. In addition, a total of 50 transcriptomes (Supplementary Data 1) from 18 paired ESCC patients were determined by RNA sequencing (RNA-seq), and four LNC samples with few tumor cells identified in the clinicopathological examination were excluded (Fig. 1a; detailed information for patients is provided in Supplementary Fig. 1a and representative histological images of normal and cancer tissues are presented in Supplementary Fig. 1b). All tumors were stage III or above. Quality control analysis showed that the Q30 score of the sequencing reads was ~90% and that the mapping ratio to the human genome was over 75% for both ChIP-seq and RNA-seq data (Supplementary Fig. 2a–d). Before element analysis, we performed quantile normalization of the H3K27ac signals to comparable levels in 28 ChIP-seq datasets as presented in box plots (Supplementary Fig. 2e, f). In this study, active typical enhancers were defined as significant H3K27ac peaks located more than 2 kb from the nearest TSSs, and regions within ±2 kb of TSSs (TSSs ±2 kb) with significant H3K27ac occupancy were considered as active promoter elements as described in previous studies^26,30^. The numbers of active promoter elements and enhancers reached saturation after 24 and 19 samples, respectively, suggesting that the majority of *cis*-regulatory elements could be retrieved with 28 samples from ten ESCC patients (Supplementary Fig. 2g, h).

Next, we established a differential analysis pipeline to identify altered *cis*-regulatory elements; meanwhile, differentially expressed genes (DEGs) were identified with DESeq2 (Supplementary Fig. 3). To check the reliability of our predicted *cis*-elements, we first overlapped our identified H3K27ac peaks (*P* value <1e-09) with the Epigenomics Roadmap dataset^31^ and found an overlap rate of ~90% (Supplementary Fig. 4a). By combining the predicted promoter and distal enhancer elements in the 28 samples together (Supplementary Fig. 4b), we recovered 24,823 active promoter elements and 57,675 active enhancers (Supplementary Fig. 5a and Supplementary Data 2). Unsupervised hierarchical clustering of enhancer profiles easily discriminated normal samples from primary EC and LNC samples (Supplementary Fig. 4c), demonstrating a significant epigenomic difference during tumourigenesis. Referring to a previous study^26^, we defined gained or lost elements as those with a fold change (FC) in H3K27ac reads per kilobase per million reads (RPKM) of ≥2 and an absolute difference of ≥0.5. Considering the large variation between patients and samples, we evaluated the distribution of altered elements in tumor or LNC/normal pairs. Approximately 80% of the altered enhancer regions were significantly different (*q* < 0.1, paired *t*-test; Benjamini–Hochberg corrected) at the threshold of ≥6/10 patients (EC vs. Nor) (Supplementary Fig. 4d, e). For the simplicity and consistency of subsequent analyses, we set the same threshold for comparisons between LNC and Nor (≥6/10) or EC (≥6/8) tissues (Supplementary Fig. 4f–i). With these criteria, we obtained a high-confidence and comprehensive set of 2917 gained and 3027 lost promoter elements and 8587 gained and 9642 lost enhancers in EC tissues (relative to Nor), 1471 gained and 1403 lost promoter elements and 4399 gained and 4626 lost enhancers in LNC (relative to Nor), and 864 gained and 715 lost promoter elements and 2733 gained and 2466 lost enhancers in LNC (relative to EC) (Fig. 1b and Supplementary Fig. 5a). These numbers were comparable to those in previous studies in other cancers^8,26^. The altered elements exhibited significant gain or loss in analyzed patients (Supplementary Fig. 5b and Supplementary Data 3) or in a representative patient (Supplementary Fig. 5c), as indicated in heatmaps.

Next, these differential enhancers were classified into six groups: G1 (EC-specific gain; *n* = 985), G2 (LNC-specific gain; *n* = 837), G3 (common gain; *n* = 1974), G4 (EC-specific loss; *n* = 871), G5 (LNC-specific loss; *n* = 548), and G6 (common loss; *n* = 2715) (Fig. 1c and Supplementary Data 4). These results demonstrate that primary and metastatic cancers have a majority (59%) of common and a minority (41%) of location-specific epigenomic features, a pattern distinct from that of promoter variances, which showed only a small number of commonly altered promoter elements (Supplementary Fig. 5d, e). Unsupervised hierarchical clustering analysis was performed with these differential enhancers, and these three types of enhancer profiles were clearly separated into group-specific traits (Fig. 1d). Similarly, the differential promoter element- and enhancer-associated genes were subjected to principal component analysis (PCA), which successfully separated normal and cancer samples along PC1; in addition, EC and LNC samples were discriminated along PC2 (Fig. 1e). Among the top 100 altered element-associated genes (50 for gain and 50 for loss), genes with positive PC1 scores (such as *GFI1*, *MSI2*, *RUNX3*, *CD72*, and *EXO1*) in the promoter analysis and genes with negative PC1 scores (such as *CD72*, *RUNX3*, *TNFSF8*, *NCF1C*, and *CTNNB1*) in the enhancer analysis were considered potential oncogenes promoting carcinogenesis (Supplementary Fig. 6a, b). In addition, genes with positive and negative PC2 scores in the promoter analysis partially overlapped with top genes with positive and negative PC2 scores in the enhancer analysis respectively, including functional regulators involved in carcinogenesis and metastasis (Supplementary Fig. 6a, b).

Considering that the epigenetic discrepancy among the three groups in distal regions, the epigenetic features of which were more dynamic among multiple patients, was much more stable than that in proximal promoter regions (Supplementary Fig. 5), we focused on investigating enhancer reprogramming in subsequent analyses. For altered enhancer-linked genes, genes with positive PC1 scores were associated mainly with the regulation of RNA polymerase II activities and epidermal development involving the Rap1 and Ras signaling pathways, while genes with negative PC1 scores were related to cell migration, the immune response, and the T-cell receptor signaling (Supplementary Fig. 6c, d). In the positive PC2 dimension with enrichment in LNC samples, the enhancer-linked genes were involved mainly in immune cell functions as well as cell shape regulation, while genes enriched in the negative PC2 direction were associated with cell adhesion, cellular organization, and pathway regulation in cancer (Supplementary Fig. 6e, f). These data validate the accuracy of sample collection and demonstrate that genome-wide alterations in distal enhancer-linked genes are potential regulators of esophageal carcinogenesis and metastasis.

SEs, a subtype of enhancers with extended physical proximity, modulate the expression of master regulators of cell identity and disease states^9,32^. Accumulating evidence suggest that SEs are enriched in cancer signatures as a hallmark in multiple cancers^23,25,26^. To examine the role of SEs in ESCC, we identified 1042 nonredundant predicted SEs in total (Supplementary Data 5) using ROSE^33^ and found that the three cohorts shared 35.7% of SE regions, higher than the percentage of common typical enhancers (Fig. 1f, g; Supplementary Fig. 6g), indicating the evolutionary conservation of SE regions across patients and cell types. It is worth noticing that the vast majority of SEs were altered, of which 436 and 427 SEs were gained and 481 and 310 SEs were lost in EC and LNC, respectively (Supplementary Fig. 6g), validating the heterogeneity of predicted SEs^23^. When PCA was performed on all of these SEs, the three groups of cancer profiles were roughly separated, with stringent overlap (Supplementary Fig. 6h).

Relative to the normal esophageal epithelium, 392 (46.3%) and 494 (52.1%) cancer-specific SEs were recovered in the EC and LNC H3K27ac profiles, respectively, and these SEs were enriched with cancer-associated genes or oncogenes, such as *CCND1*^25,26^, *CWH43*, *ANO1*, and *CXCR4* (Fig. 1f, g and Supplementary Fig. 7). For example, neighboring regions of *CCND1*, *CLUAP1*, and *MTMR8* showed a significant gain of promoter elements, enhancers, and SEs in cancer tissues, and loss of promoter elements, enhancers, and SEs was observed in *TFAP2B*, *MAU2*, and *EHF*, accompanied by transcriptional activation (*CCND1*) or repression (*EHF*) (Fig. 1h and Supplementary Figs. 6i, 7). Our consistent retrieval of previously identified distal enhancers (such as *CCND1* at the rs7105934 locus) from other cancers^25,26,34^ in our unbiased profiling of H3K27ac enrichment further supports the reliability of our data.

### Enhancer alterations correlate with aberrant transcriptional programs in ESCC

**Fig. 2:**
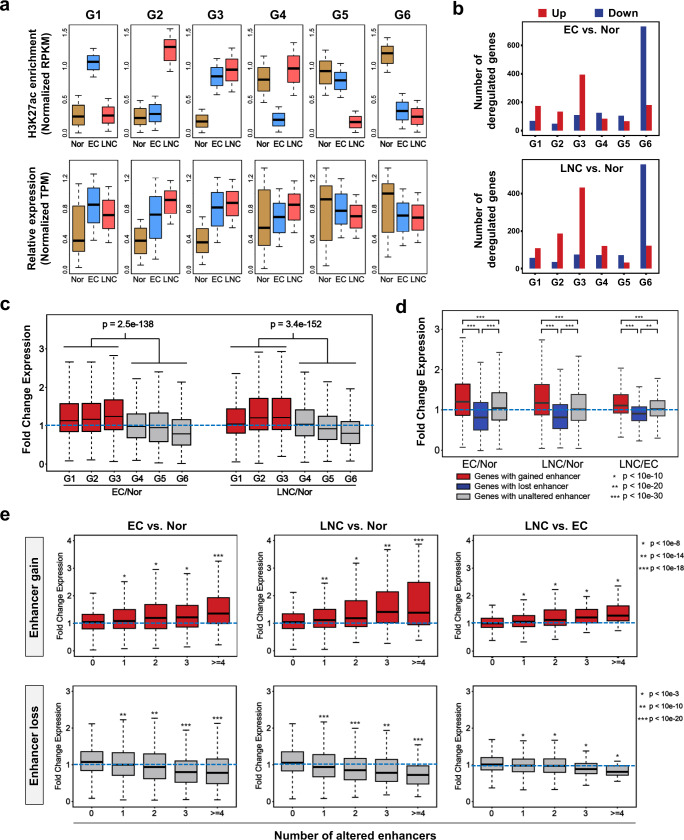
Gained and lost enhancers contribute to aberrant gene expression. **a** Normalized H3K27ac peak enrichment in (upper panel) and expression (lower panel) of differential enhancer (G1–G6)-associated genes in Nor (*n* = 10), EC (*n* = 10), and LNC (*n* = 8) samples. The *P* values for all comparisons were <0.001, as calculated by the Kruskal–Wallis rank-sum test. Boxes correspond to interquartile ranges (IQR), thick black lines indicate the median values, and the whiskers extend to the lowest or highest data point that are still within 0.5 IQR of the bottom or top quartile, respectively. **b** Numbers of upregulated and downregulated genes (EC vs. Nor or LNC vs. Nor) linked to differential enhancers (G1–G6). **c** The expression fold changes (EC/Nor or LNC/Nor) in differential enhancer (G1–G6)-associated genes. *P* value was calculated by the two-sided Wilcoxon test (no adjustments). **d** The expression fold change (EC/Nor, LNC/Nor, or LNC/EC) in all differential enhancer-associated genes (EC vs. Nor, LNC vs. Nor, or LNC vs. EC) from Nor (*n* = 10), EC (*n* = 10), and LNC (*n* = 8) samples. *P* value was calculated by the two-sided Wilcoxon test (no adjustments). *P < 10e-10, **P < 10e-20, ***P < 10e-30. **e** The expression fold changes in genes associated with the indicated number of gained (red) and lost enhancers (gray) are presented. *P* value was calculated by the two-sided Wilcoxon test (no adjustments). Upper panel: *P < 10e-8, **P < 10e-14, ***P < 10e-18. Lower panel: *P < 10e-3, **P < 10e-10, ***P < 10e-20. c–e, Data points represent means of Nor (*n* = 10), EC (*n* = 10), and LNC (*n* = 8) samples. Boxes correspond to interquartile ranges (IQR), thick black lines indicate the median values, and the whiskers extend to the lowest or highest data point that are still within 1.5 IQR of the bottom or top quartile, respectively. Source data are provided as a Source Data file.

To explore relationships between epigenomic enhancer alterations and gene expression, we analyzed the RNA-seq data from the same cohorts as well as 22 transcriptomes from eight additional ESCC patients. As previously reported^9^, we assigned predicted enhancers to nearby genes with the nearest TSSs, and then analyzed the expression of differential enhancer-linked genes in the six groups (Fig. 1c). As expected, the expression of genes with nearby differential enhancers was generally positively correlated with H3K27ac enrichment signals, especially for the common gained (G3) and lost (G6) enhancers (Fig. 2a). In the groups with more altered enhancers, the numbers of upregulated genes in G3 and downregulated genes in G6 were much larger than those in the other groups (Fig. 2b). Genes linked to gained enhancers (G1–3) were generally expressed at higher levels in EC or LNC samples, while genes linked to lost enhancers (G4–6) were expressed at lower levels in cancer samples than in normal epithelium (Fig. 2c). For differential enhancer-linked genes with detectable expression levels, the expression of a relatively high percentage of DEGs followed the pattern of H3K27ac alterations, especially in the G3 (*n* = 413; 78.8%) and G6 (*n* = 442; 70.8%) groups, although the expression of some genes was not consistent with the patterns of their nearby H3K27ac marks (Supplementary Fig. 8a, b and Supplementary Data 6). Moreover, the total number of DEGs was larger than the number of altered enhancer-linked DEGs (Supplementary Fig. 8c and Supplementary Data 7), possibly because DESeq2 was used for DEG analysis (*n* = 18) without considering the pairing of patients and the threshold of ≥6/10 patients used for differential enhancer identification (Supplementary Fig. 3). These results indicate that enhancer acquisition and loss is a reliable and stable signature of primary and metastatic ESCC and is even more distinctive and predictive than aberrant gene expression (Supplementary Fig. 8c)^35^.

When considering the difference among the three cohorts, we showed that the expression of genes with gained enhancers was significantly upregulated and that of genes associated with lost enhancers was markedly downregulated, compared to that of control genes with unaltered enhancers, in the three groups of comparisons (Fig. 2d), further verifying the control of gene dysregulation by enhancer alterations. In addition, although a single altered enhancer was sufficient to induce aberrant gene expression, an increased number of gained enhancers was positively correlated with gene activation, while an increased number of lost enhancers was negatively correlated with gene repression in a quantitative manner (Fig. 2e). It further demonstrates that enhancer alterations and reprogramming contribute to the aberrant transcriptional program in primary and metastatic tumors. To identify biomarkers for EC and LNC, we selected G1-, G2-, G3-, and G6-associated genes as input, and analyzed the correlation between their mean expression levels and H3K27ac signals (exemplified by *EHD3*, *ANO1*, and *EN1*); for example, the H3K27ac level in an *EHD3* enhancer was highly correlated with its expression (*r* = 0.86, Spearman correlation) (Supplementary Fig. 9a, b and Supplementary Data 8). The top 100 genes with a positive correlation were ranked by their expression FCs, and the top 10 or 15 genes were assigned to subgroup g1 (*FAM19A5*, *LAMC2*, *SNAI2*, *PMEPA1*, etc.), g2 (*TCF7*, *KLHL6*, *SCIMP*, *CXCR4*, etc.), g3 (*FCGR2A*, *HOXD11*, *ZNF469*, *CLDN3*, etc.), and g6 (such as *TFAP2B*, *MAL*, *EMP1*, *EHD3*, etc.) with region-specific enhancer and transcript enrichment (Supplementary Fig. 9c, d and Supplementary Data 9). The combinations of these four groups of genes were defined as biomarkers for Nor (g1^−^ g2^−^ g3^−^ g6^+^), EC (g1^+^ g2^−^ g3^+^ g6^−^), and LNC (g1^−^ g2^+^ g3^+^ g6^−^) tissues (Supplementary Fig. 9e).

Gene Ontology (GO) and Kyoto Encyclopedia of Genes and Genomes (KEGG) pathway analyses showed that gained enhancers in EC and LNC were enriched in essential cancer processes, such as cell proliferation, motility, and migration, and were associated with genes involved in proteoglycans, chemokine signaling, transcriptional misregulation, Rap1 signaling, and other pathways in cancer (Supplementary Fig. 10a, b). Importantly, consistent with the PCA results (Supplementary Fig. 6), gained enhancers in LNC were associated with cell migration as well as immune system processes regarding the T-cell receptor signaling, which might be elicited by the mixture of minimal immune cells from lymph nodes (Supplementary Fig. 10a, b). However, we also observed that relative to those in EC samples, the gained enhancers in LNC samples were enriched in cell adhesion, cell migration, and other metastatic traits, with the activation of cancer-related pathways (Supplementary Fig. 10c, d). Interestingly, some cancer-specific features, including PI3K-AKT signaling, HIF1 signaling, and Hippo signaling^36^, emerged as EC-specific features but were diminished in LNC samples (Supplementary Fig. 10d). Intriguingly, we found that some genes with nearby active enhancers were not transcriptionally activated in specific states. It has been proposed that the epigenetic patterning and information of enhancers may be established and embedded within enhancer elements before cellular processing^37^. Therefore, we selected the genes for which the nearby enhancers were pre-activated (such as *SORL1*, *BCL2*, *KLHL6*, and *CLDN3*) or pre-silenced (such as *MAPK6*, *ETS2*, *LCN2*, and *LGALSL*) before transcriptional alterations in EC samples, and considered that these enhancers or their associated genes might be the priming factors for the initiation of cancer metastasis-related processes (Supplementary Fig. 11 and Supplementary Data 10). Collectively, we propose that enhancer reprogramming confers aberrant gene expression in esophageal carcinogenesis and metastasis.

### Super-enhancer signatures and heterogeneity in primary and lymph node tumors

**Fig. 3:**
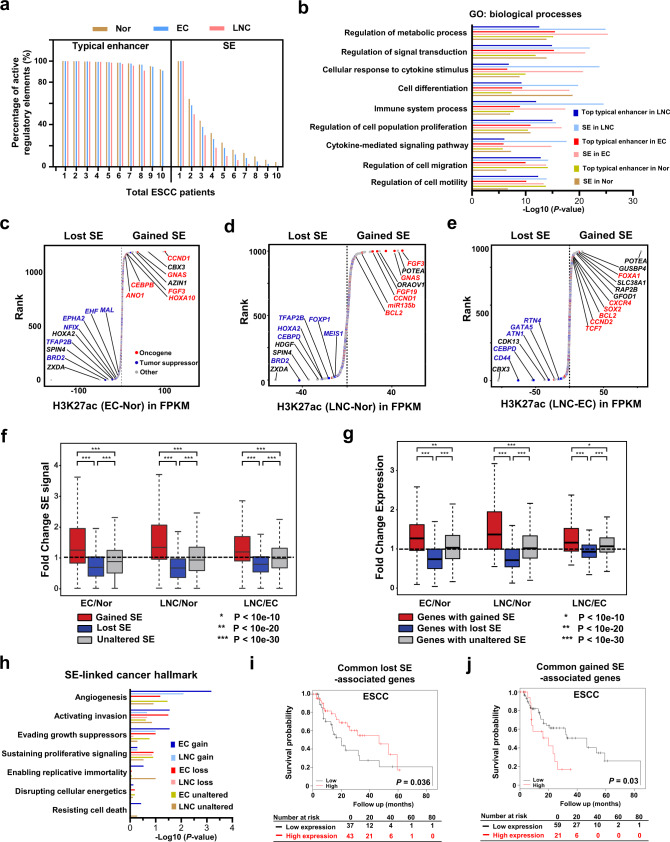
Super-enhancer signatures in primary and lymph node tumors. **a** Percentage of predicted typical enhancers and super-enhancers (SE) across Nor (*n* = 10), EC (*n* = 10), and LNC (*n* = 8) tissues showing H3K27ac enrichment above that of randomly selected regions (99%) across an increasing number of patients. **b** Gene Ontology (GO) analysis of the top 2000 predicted typical enhancer-linked genes and SE-associated genes; the top significantly associated biological processes are presented. **c**–**e** A total of 1317 super-enhancers are ranked by their differential H3K27ac intensity between EC and Nor, LNC and Nor, as well as LNC and EC samples. Genes associated with the top gained and lost SEs are listed; oncogenes are highlighted in red, and tumor suppressors are highlighted in blue. **f**, **g** Fold changes in H3K27ac enrichment signals (**f**) and expression levels for differential SE-associated genes (**g**) (EC/Nor, LNC/Nor, or LNC/EC) and for differential SE (gained and lost)-associated genes (EC vs. Nor, LNC vs. Nor, or LNC vs. EC). Unaltered SE-associated genes were used as controls. Data points represent means of Nor (*n* = 10), EC (*n* = 10), and LNC (*n* = 8) samples. Boxes correspond to interquartile ranges (IQR), thick black lines indicate the median values, and the whiskers extend to the lowest or highest data point that are still within 1.5 IQR of the bottom or top quartile, respectively. *P* value was calculated by the two-sided Wilcoxon test (no adjustments). *P < 10e-10, **P < 10e-20, ***P < 10e-30. **h** Cancer hallmark analysis using differentially predicted SEs showing recurrently gained, recurrently lost, and unaltered H3K27ac signals in EC or LNC relative to Nor. The log (adjust *P* value) obtained from the hypergeometric test is shown. **i**, **j** Survival analysis comparing groups of patients with high or low expression of the top common lost (**i**) or gained (**j**) SE-associated genes in ESCC TCGA data using Kaplan–Meier plotter. A poor prognosis observed for ESCC patients with tumors possessing a high expression signature of gained SE-associated genes and a low expression signature of lost SE-associated genes. Survival data are presented every 20 months. A Log-rank test was performed for survival data. Source data are provided as a Source Data file.

A much higher proportion of SEs than typical enhancers were recurrently gained or lost in esophageal or LNC (Supplementary Figs. 5a and 6g). However, the active typical enhancers were relatively consistent in the same cohort of profiles among multiple patients and were more stable than in multiple cancer cell lines^23^, while SEs varied with a modest recurrent tendency (estimated recurrence rates of 6–16% in ≥5/10 or 5/8 patients) among multiple patients (Fig. 3a), indicating the heterogeneity of SEs in primary and lymph node tumors.

GO analysis showed that the SEs in the EC and LNC cohorts were enriched in genes involved in the regulation of metabolic processes, signal transduction, responses to cytokines, cell proliferation, cell migration, and cell mobility, and the enrichment was more significant than that of the GO terms predicted from the top 2000 typical enhancers with the strongest average H3K27ac signals (Fig. 3b). Because the vast majority of SEs were recurrently gained or lost in EC or LNC samples, we propose that consistent with our previous study^11^, enhancer reprogramming (especially of SEs) may predominantly mediate cellular responses to cytokines, such as TGFβ signaling, to promote tumourigenesis and metastasis.

Using the previously reported strategy^23,26^ to map gained or lost SEs (Supplementary Fig. 6g), we identified some putative targets of the top altered enhancers (Supplementary Data 11), including some notably gained SE-linked oncogenes associated with cell activation, communication, adhesion, and motility (such as *CCND1*, *GNAS*, *FGF3*, *HOXA10*, and *CEBPB*), as well as many lost SE-linked genes (such as *EHF*, *TFAP2B*, *NFIX*, *MAL*, and *MEIS1*) involved in epithelial development, morphogenesis, and developmental processes (Fig. 3c, d and Supplementary Fig. 12a). Interestingly, the putative targets of lost SEs highly overlapped with those reported in RCC, and these genes were expressed at low levels in tumor tissues but at high levels in normal tissues (Fig. 3c, d)^26^, indicating the conserved mechanisms underlying the loss of epithelial characteristics. Moreover, the SEs gained in LNC relative to EC were enriched mainly in the immune system and oncogenic features (Supplementary Fig. 12b), as represented by *FOXA1*, *CXCR4*, *SOX2*, *CCND2*, etc. (Fig. 3e). Compared with the trends in transcriptional changes associated with enhancer alterations (Fig. 2d), the gained or lost SEs-associated genes were deregulated with increased FCs in gene upregulation or downregulation, respectively (Fig. 3f, g). We next performed cancer hallmark analysis^9^ to further explore the biological significance of the predicted SE heterogeneity with a gain or loss status. In line with the GO analysis results (Supplementary Fig. 12a, b), somatic gained SEs were enriched in genes related to angiogenesis, invasion, and evasion of growth suppressors, and this enrichment was more significant than that for the somatic loss and unaltered categories (Fig. 3h). These data indicate that gained somatic SEs in EC and LNC can be used to predict the progression and aggressiveness of ESCC and LNC, and highlight the importance of SEs in carcinogenesis.

The heterogeneity and variance of SEs in clinical sample profiling (Supplementary Fig. 12c) may lead to some top-ranked differential SEs exhibiting a large absolute difference value but a small FC. Therefore, we established another pipeline to rank the differential predicted SEs considering sample pairing for each patient with a threshold of ≥5/10 or 5/8 (for LNC analysis) patients (the SEs identified in individual samples and recurrently identified in ≥3 patients are listed in Supplementary Data 12 and Supplementary Fig. 12d). The numbers of gained and lost SEs obtained in each group were slightly greater than those calculated from the mean signals, with a large proportion of overlapping altered SEs (Supplementary Fig. 12e). To avoid missing essential SEs, we combined the differential SEs identified through the two strategies and ranked these SEs by their FC (Supplementary Fig. 12f and Supplementary Data 12). The SEs with the highest FC (FC >2 for gain and FC <0.7 for loss; because there were much fewer lost SEs than gained SEs) overlapped with those with the highest absolute difference values (>2 for gain and < −2 for loss), and dozens of high-confidence altered SEs were identified (Supplementary Fig. 12f). As shown in Supplementary Figs. 13a and 14 (Supplementary Data 13), H3K27ac signal gain or loss was positively correlated with the upregulation or downregulation patterns of nearby genes, respectively, in a stringent manner, and numerous top representative genes with gained (such as *FGF3, ANO1*, *HOXA9*, and *CCND1*) or lost (such as *NFIX*, *TFAP2B*, and *HOXA3*) SEs overlapped with the results shown in Fig. 3c–e. These gained SE-linked genes in EC or LNC were also associated with tumor invasion and inflammation processes (Supplementary Fig. 13b). Taking together, we identified four groups of deregulated genes associated with the differential SEs (*n* = 456) (Supplementary Data 14), the majority of which were commonly gained or lost in EC and LNC, along with a minor group of genes with LNC-specific gained or lost SEs (exemplified by *MEIS1* and *CXCR4*) (Supplementary Figs. 13c and 14), consistent with altered typical enhancer-linked genes (Supplementary Fig. 8b).

To explore the potential roles of the putative targets of the predicted SEs in other cancers, we analyzed the expression of differential SE-linked genes (Supplementary Fig. 13c) in primary and metastatic pancreatic adenocarcinoma (PDA) and colorectal cancer (CA) datasets from The Cancer Genome Atlas (TCGA). Surprisingly, we identified 78 and 212 DEGs that showed either common gain/loss or metastasis-specific gain/loss patterns in PDA and CA, respectively, among which the common upregulated (gain) and downregulated (loss) genes accounted for the majority of the DEGs (Supplementary Fig. 15a, b and Supplementary Data 15). The examined DEGs from ESCC, PDA, and CA overlapped, demonstrating that many DEGs are coincident among these three cancers, especially those in the groups with common gain or loss between ESCC and CA (Supplementary Fig. 15c); 12 genes, including *ANO1*, *CTTN*, *PYGB*, *SOX4*, *SOX9*, *PMEPA1*, etc., were commonly upregulated in all three types of cancers (Supplementary Fig. 15d and Supplementary Data 16), an effect possibly mediated by SE activation. We also identified six genes (*KHDRBS2*, *IQSEC1*, *ARHGEF1*, *ARID5A*, *IRX5*, and *TMC6*) that were activated in metastatic cancers in two of the three cancer types (Supplementary Fig. 15d) and might be associated with metastatic traits. To elucidate the clinical relationship between altered SEs and ESCC patient survival, we identified differential SE-linked genes that displayed a high correlation with the expression of their target genes and performed survival analysis via Kaplan–Meier Plotter with TCGA data. Higher expression of common lost SE-associated genes predicted a higher probability of patient survival (Fig. 3i), while higher expression of common gained SE-associated genes predicted a lower probability of survival in EC, PDA, and CA patients (Fig. 3j and Supplementary Fig. 15e–g). It demonstrates the close relationship between cancer-associated SEs and clinical events during ESCC carcinogenesis.

### Transcription factor circuitries of ESCC and LNC

**Fig. 4:**
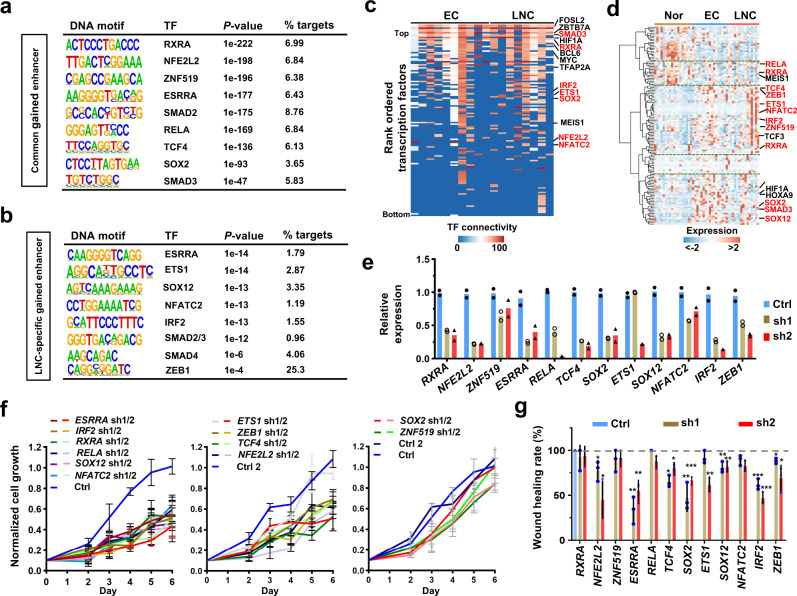
Transcription factor circuitries of esophageal carcinogenesis and metastasis. **a**, **b** Top transcription factor (TF) binding motifs predicted from common gained (**a**) or LNC-specific gained (**b**) enhancers using HOMER. The predicted TFs are ranked by their *P* value (two-sided binomial test), and the proportion of targets potently regulated by these TFs is also presented. **c** Heatmap of TFs ranked by their predicted activity using core circuitry analysis. Representative genes with high TF connectivity are presented. **d** Heatmap showing the expression of common gained or LNC-specific gained enhancer predicted TFs in the Nor, EC, and LNC groups. The TFs shown in **a**, **b** are highlighted in red. **e** Assessment of knockdown efficiency by shRNAs. Two shRNAs were constructed for each TF, and shRNA expressing TE1 cells were subjected to quantitative real-time PCR analysis. The expression of each TF in control cells expressing the control shRNA was normalized to “1”. Ctrl, control. *n* = 2 biologically independent experiments examined. **f** Control or shRNA expressing TE1 cells were counted using a cell counter and control cells were duplicated in this experiment (Ctrl and Ctrl2). The number of cells from three wells was averaged as a single replicate for each sample, and results were obtained from three replicates. The time course curve of the normalized cell number was plotted. Data were presented as mean values ± s.d. *n* = 3 biologically independent experiments examined. **g** Wound healing assays were performed with control and shRNA expressing TE1 cells, and the wound healing rate was calculated for each group. The wound healing rate was calculated as shown in Supplementary Fig. 17b. Data were presented as mean values ± s.d. *n* = 3 biologically independent experiments examined. Statistics: *P < 0.05, **P < 0.01, ***P < 0.001, unpaired *t*-test. Source data are provided as a Source Data file.

The core circuitry of cell type- or status-specific transcriptional regulation is often dominated by several key TFs to establish context dependence^38^. To identify principal TFs that promote ESCC tumourigenesis and metastasis, we predicted *trans*-acting factors associated with common gained or LNC-specific gained enhancers using HOMER^39^. Binding motifs for many essential TFs, such as RXRA, NFE2L2, ESRRA, IRF2, RELA, ESRRA, SOX2, and SMAD2/3 (Fig. 4a, b), key TFs that mediate TGFβ signaling in epithelial-mesenchymal transition (EMT) and cancer metastasis^11,40^, were enriched in common gained or LNC-specific gained enhancers, with some overlapping TFs. Core regulatory circuitry analysis^25,41^ further showed that many TFs predicted by gained enhancers, including SMAD3, RXRA, IRF2, ETS1, etc., were displayed among the top rank-ordered TFs interacting with other predicted TFs (Supplementary Data 17); the identification of FOSL2, SOX2, and RXRA (Fig. 4c), which were also previously revealed in ependymoma^25^, supports the reliability of our data and strategy. Notably, the majority of the top enriched TFs were highly expressed in the EC and/or LNC groups compared to the normal esophageal epithelium group (Fig. 4d and Supplementary Data 18). The *trans*-acting factors predicted by commonly lost enhancers, such as TBX21, NFIA, MEIS1, and ELF3, were involved in epithelial development (Supplementary Fig. 16a), while the functions of some LNC-specific lost enhancer predicted TFs remained largely unclear in LNC (Supplementary Fig. 16b). Interestingly, many TFs predicted by commonly lost enhancers were negatively correlated with TFs predicted by common gained or LNC-specific gained enhancers (Supplementary Fig. 16c and Supplementary Data 19), indicating the distinctive roles of these TFs in normal and cancer tissues.

Two independent short hairpin RNAs (shRNAs) for each target were used to investigate the functional roles of altered enhancer-predicted top-ranked TFs by efficiently knocking down the corresponding TFs (RXRA, NFE2L2, ZNF519, ESRRA, RELA, TCF4, SOX2, ETS1, SOX12, NFATC2, IRF2, and ZEB1), with individual low efficient shRNAs (i.e., *ETS1* shRNA1 and ZNF519 shRNA2) (Fig. 4e). Then, cell growth was assessed in TE1 esophageal cancer cells expressing the control shRNA or shRNAs targeting these 12 TFs, showing that the depletion of *IRF2*, *RXRA*, *RELA*, *SOX12*, and *NFATC2* resulted in a significant decrease in cell proliferation and that the knockdown of *ETS1* (with shRNA2), *ZEB1*, *TCF3*, and *NFE2L2* led to a modest decrease in cell growth (Fig. 4f and Supplementary Fig. 17a). Wound healing assays further demonstrated that depletion of *NFE2L2*, *ESRRA*, *TCF4*, *SOX2*, *ETS1* (with shRNA2), SOX12 (weak but significant), and *IRF2* resulted in impaired cell migration (Fig. 4g and Supplementary Fig. 17b). Consistent with these cellular phenotypes, the knockdown of some key TFs, such as *IRF2*, RELA, *RXRA*, and *NFE2L2*, induced upregulation of the cell cycle inhibitor *p21* and downregulation of the oncogene *C-Myc* and the EMT inducers *SNAI1* and *FN1* (Supplementary Fig. 17c).

Moreover, the binding sites of eight TFs in cancer cell lines (data available from ENCODE; the data source is provided in Supplementary Data 20) largely overlapped with predicted enhancers (Supplementary Fig. 18a); dozens to hundreds of common or LNC-specific gained enhancers that were associated with important oncogenesis- or metastasis-related genes, such as *ZXDC*, *CTNNB1*, *CXCR5*, *CCND2*, *STAT3*, and *SNAI1*, were bound by IRF2, RXRA, RELA, or ZEB1 (Supplementary Fig. 18b and Supplementary Data 20). Thus, we propose that these TFs mediate the enhancer gain process. In summary, we successfully identified several important TFs that regulate ESCC carcinogenesis and metastasis through the prediction of *trans*-TFs by gained enhancers.

### Gained SE maps identify candidate drugs against ESCC carcinogenesis and metastasis

**Fig. 5:**
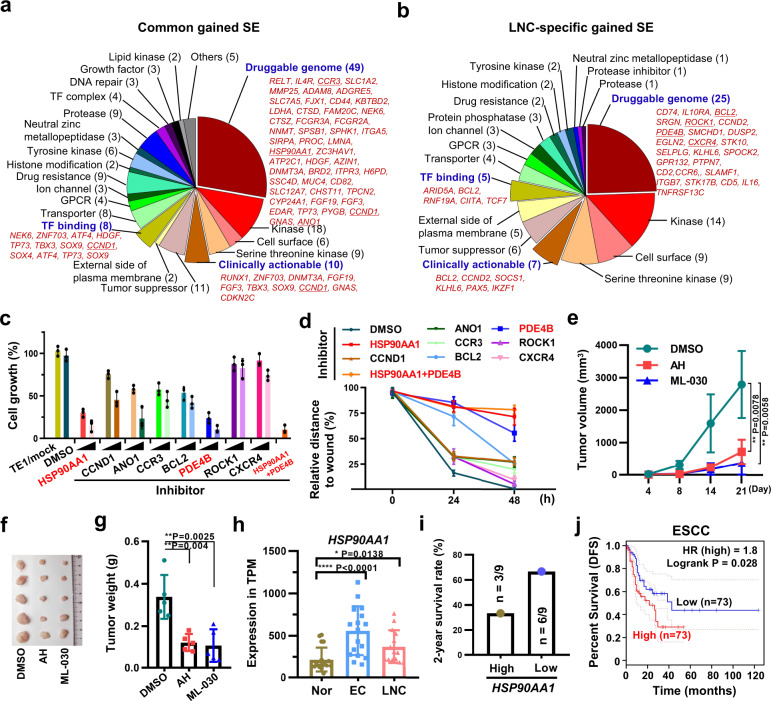
Active regulatory super-enhancers predict candidate drugs against esophageal carcinogenesis and metastasis. **a**, **b** Pie charts showing candidate drug compounds detected through the integrated analysis of common gained (**a**) and LNC-specific (**b**) super-enhancers with the Washington University Drug Gene Interaction Database. Three categories are highlighted in red. **c** TE1 cells were treated with eight chemical inhibitors separately or combinationally (AH + ML-030). Cells were counted at 72 h. Data were presented as mean values ± s.d. *n* = 3 biologically independent experiments examined. **d** Wound healing assays for control and shRNA expressing TE1 cells. The wound width was examined every 24 h, and the wound width at 0 h was normalized to “1”, and the relative width to the width at 0 h was calculated. Data were presented as mean values ± s.d. *n* = 3 biologically independent experiments examined. **e**–**g** Xenograft analysis of tumor growth. Immunodeficient nude mice injected with TE1 cells were treated with AH or ML-030, and tumor volumes were determined (**e**). The tumors were collected from sacrificed mice on day 21 for image acquisition (**f**) and tumor weight determination (**g**). For pooled data from five repeats, values indicate the mean ± s.d. A presentative data from *n* = 2 independent experiments was shown. **e** two-way ANOVA test with Geisser-Greenhouse correction, **P < 0.01. **g** unpaired *t*-test (two-tailed), **P < 0.01. **h** Expression of *HSP90AA1* in Nor, EC, and LNC samples from our RNA-seq data. Data were presented as mean values ± s.d. *n* = 18 for Nor and EC; *n* = 14 for LNC. *P < 0.05, ****P < 0.0001, unpaired *t-*test (two-tailed). **i** The 2-year survival rate for two groups of patients (*n* = 9 for each group) possessing high or low *HSP90AA1* expression as determined from clinical data in the present study. *n* = 3/9 (33.33%) in *HSP90AA*1-high and *n* = 6/9 (66.67%) *HSP90AA*1-low patients, presented as a single 2-year survival rate, were survival till Sep 2020. **j** The disease-free survival (DFS) rate was analyzed by comparing ESCC patient groups with high (*n* = 73) or low (*n* = 73) *HSP90AA1* expression using GEPIA. Source data are provided as a Source Data file. A Log-rank test was performed for the survival data.

To screen candidate drugs for clinical use in ESCC and lymph node metastasis, we performed an integrated analysis of our tumor-specific SE-linked genes with the Washington University Drug Gene interaction database^42^, informing 69 common gained SE-linked genes and 43 LNC-specific gained SE-linked genes, which were redundantly categorized into 21 and 18 classes, respectively, including druggable genome, kinase, clinically actionable, cell surface, TF binding, etc. (Fig. 5a, b and Supplementary Data 21). Among the 49 and 25 putative druggable genome targets, our analysis revealed 236 drugs interacting with 36 druggable genes associated with commonly gained SEs and 170 drugs interacting with 18 druggable genes associated with LNC-specific gained SEs (Supplementary Fig. 19a and Supplementary Data 21). Because many genes can be targeted by several drugs, we ranked these genes according to the numbers of interactions with drugs, and the top eight druggable targets with the most interactions (HSP90AA1, CCND1, ANO1, and CCR3 from the commonly gained SEs; and BCL2, PDE4B, ROCK1, and CXCR4 from the LNC-specific gained SEs), which were identified as candidate genes responsive to small molecule inhibitors, were selected for subsequent functional investigations using commercial chemical inhibitors (Supplementary Fig. 19b). Except for the ROCK1 and CXCR4 inhibitors, six inhibitors, particularly the HSP90AA1 and PDE4B inhibitors, markedly suppressed TE1 cell growth in a dose-dependent manner, and these two inhibitors exhibited synergistic effects on cell proliferation (Fig. 5c and Supplementary Fig. 19c, d). Moreover, a high rate of cell apoptosis was induced by HSP90AA1 or PDE4B inhibitors or dual inhibitors (Supplementary Fig. 19e, f). Notably, the inhibitory effects on cell proliferation (Supplementary Fig. 20a–d) and proapoptotic effects (Supplementary Fig. 20e, f) of HSP90AA1 inhibitor and PDE4B inhibitor were perfectly validated in another two ESCC lines (KYSE30 and KYSE150). Thus, we demonstrate that the inhibition of HSP90AA1 or PDE4B activity substantially suppresses cell proliferation and induces cell apoptosis.

To assess the functions of these gained SE-linked putative targets in cancer metastasis, we performed wound healing assays upon inhibitors treatment and found that nearly all of these applied inhibitors showed strong (HSP90AA1, PDE4B, and BCL2) or weak (ANO1, CCR3, CCND1, and CXCR4) suppressive effects on cell migration (Fig. 5d and Supplementary Fig. 19g). Furthermore, treatment of immunodeficient mice bearing esophageal cancer xenografts (TE1 cell-derived) with alvespimycin hydrochloride (AH, an HSP90AA1 inhibitor) or ML-030 (a PDE4B inhibitor) remarkably suppressed tumor growth (Fig. 5e–g). Coincidently, the expression of *HSP90AA1* and *PDE4B* as well as their nearby SEs was more significantly enriched in the EC and LNC groups than in the normal control group (Fig. 5h and Supplementary Fig. 21a, b), validating their potential as clinically achievable antitumor targets. Considering the high expression of *HSP90AA1* relative to *PDE4B* in EC and LNC samples, we prioritized *HSP90AA1* as a potential clinical diagnostic and therapeutic target. We divided 18 EC patients recruited in the present study into two groups with high or low *HSP90AA1* expression in EC samples (Supplementary Fig. 21c) and found that the 2-year survival rate of *HSP90AA1*-low patients (66.7%, *n* = 6/9) was twofold higher than that of *HSP90AA1*-high patients (33.3%, *n* = 3/9) (Fig. 5i). Although the survival curve showed no significant difference, the *HSP90AA1*-low group displayed a better survival trend in our analysis of a limited number of patients (Supplementary Fig. 21d). To support our hypothesis, we analyzed the survival probability of multiple types of cancer patients in the TCGA database, showing that lower *HSP90AA1* expression predicted better survival and prognosis in ESCC, EAC, head-neck squamous cell carcinoma, and liver cancer (Fig. 5j and Supplementary Fig. 21e–g). Considering the specific gain of PDE4B SEs, we tested the effect of PDE4B inhibition in cancer metastasis. As we expected, ML-030 treatment remarkably reduced the metastasis of KYSE150 cell-derived primary tumors in an intramuscular injection model to mimic primary EC (Supplementary Fig. 21h). These data suggest that the SE landscape can inform therapeutic targets, such as HSP90AA1 and PDE4B, to combat ESCC carcinogenesis and metastasis. Given that these gained SEs might be mediated by key TFs (Fig. 3), we analyzed the chromatin architectures around representative gained SEs near the *ANO1*, *HSP90AA1*, and *PMEPA1* genes, showing that enhancer gain or loss occurred mainly within the same topologically associating domains (TADs), which were analyzed from a set of Hi-C data in fetal lung fibroblasts (IMR90)^11^, with some predicted TFs binding sites (Supplementary Fig. 22a). Therefore, we examined the expression of three putative targets of gained SEs (*ANO1*, *HSP90AA1*, and *PMEPA1*) upon TF depletion, demonstrating that the knockdown of some key TFs, which may mediate SE gain, resulted in the downregulation of *ANO1*, *HSP90AA1*, or *PMEPA1* expression (Supplementary Fig. 22b). Collectively, our results identify primary and metastatic tumor-specific enhancers and SEs as well as their driven TFs, reveal a molecular basis for the regulation of oncogenic transcriptional programs, and provide a strategy for discovering therapeutic targets involved in ESCC carcinogenesis and metastasis.

## Discussion

Esophageal cancer is a severe disease with high mortality, accompanied by poor prognosis and frequent metastasis to lymph nodes; in particular, its survival rate is inversely correlated with its metastasis and recurrence. In addition to surgery and chemotherapy, very few chemical drugs targeting biological molecules are clinically approved^1–4^. Therefore, for this kind of clinically heterogeneous disease, understanding its epigenomic deregulation signatures has emerged as a pivotal strategy to identify biomarkers, vulnerabilities, and therapeutic targets for this clinically heterogeneous disease^23^. By profiling histone H3K27ac modifications in paired normal, primary, and lymph node tumor pairs, we generated a comprehensive compendium of promoter and enhancer alterations in ESCC.

Our study characterizes a comprehensive epigenetic profile of primary and metastatic ESCC tumors beyond genomic alterations^17^ and provides a valuable resource of epigenomic information for investigating cancer metastasis. Similar to a recent report regarding DNA methylation alterations in ESCC^43^, the significance of our work was partially limited by the limited sample size; a larger number of patients may help to dissect more clinical and therapeutic highlights, such as clinical subgrouping and personalized treatment, as reported previously in EAC^44^. Second, we show that the epigenomic and transcriptomic profiles of LNC are quite similar to those of primary tumors, with some LNC-specific characteristics. It suggests that targeting the common gained enhancer-linked oncogenic targets in primary and metastatic tumors, such as HSP90AA1, is a potential strategy for ESCC therapy. In addition, the blockade of LNC-specific enhancer-linked genes may specifically inhibit cancer cell metastasis. It demonstrates that epigenomic landscapes, which might be more stable than transcriptional differences^35^, are able to dissect the molecular differences between similar tumor entities. Third, we demonstrate that variant enhancer and super-enhancer reprogramming contributes to transcriptional remodeling, which is further emphasized by the reverse engineering of core transcriptional regulatory circuitries functionally involved in tumor cell proliferation and migration (Fig. 4). Finally, integrated analysis of somatically gained SEs with drug interaction databases enables us to identify and validate potent master regulators and cancer dependencies that are responsive to pharmacologic drugs or inhibitors (Fig. 5), highlighting the reliability of the enhancer landscape to inform precision therapies obtained from drug databases.

In recent years, epigenomic studies regarding promoter alterations^24,45^ and distal enhancer dynamics have illustrated a fundamental role for epigenomic elements in controlling the cell status in human diseases and cancers^8,9,23,25,26,46^, which motivate us to investigate a consistent epigenomic feature of carcinogenesis and metastasis in esophageal cancer. Because of the limitations of obtaining an appropriate number of cells with high purity from donors for ChIP-seq analysis, here we mapped only the H3K27ac profiles (Fig. 1), similar to the approach used in a recent report regarding ependymoma;^25^ however, although enhancer-specific histone H3 lysine 4 mono-methylation (H3K4me1) profiling will be quite helpful for precisely separating active promoter- and enhancer-enriched H3K27ac signals in promoter regions^26^. Therefore, we focused mainly on the distal enhancers outside promoter regions, which account for the majority of enhancer-linked *cis*-regulatory elements^47^. The identification of promoters herein using H3K27ac alone was not accurate, so we defined TSS ±2 kb regions with significant H3K27ac occupancy as active promoter elements (including promoters and a few proximal enhancers) but not as active promoters. Considering the neighboring occupancy between enhancer-marked H3K27ac and H3K4me3 (tri-methylation) around TSSs, the number of our identified active promoter elements was comparable to that of H3K27ac + /H3K4me3 + -defined active promoters within TSS ±2 kb regions reported in another work^26^, indicating similarities between active promoter elements and active promoters.

Although a large number of nearest deregulated genes were associated with altered enhancer elements (Fig. 2), there were still hundreds of DEGs that were not positively correlated with nearest H3K27ac alterations (Supplementary Fig. 8). As reported previously, approximately half of the predicted enhancer/gene or SE/gene interactions directly regulate the nearest proximal genes^23,48^. To build a perfect distal interaction model capable of identifying enhancers and paired target genes within a TAD, it will be meaningful to generate ESCC-specific Hi-C data; alternatively, integrating the altered enhancers or SEs with data from interaction datasets, such as PreSTIGE30, GREAT31, and RNAPII ChIA-PET, is another feasible strategy^23^ to accurately map the functional distal enhancers. Additionally, we identified a subgroup of genes, with delayed expression changes after H3K27ac alterations (Supplementary Fig. 11), which indicates that these enhancers may prime the transcriptional regulation of their targets. This possibility may also account for part of the inconsistency between gene expression and nearby H3K27ac alterations. Intriguingly, it has been proposed that the adjacent “normal” tissues may contain accumulated prevalent genomic alterations^49–52^. Investigating the link between epigenomic alterations and genomic mutations in the adjacent normal tissues will be quite meaningful.

Compared with those in matched nonmalignant esophageal tissues, recurrent predicted SEs in EC and LNC tissues were identified at known oncogenes, such as *CCND1*, *CEBPB*, and *RXRA*^25,26^, and at essential oncogenic promoters. We also observed a wide range of SE variations between different patients, with a heterogeneity markedly exceeding that of typical enhancers (Fig. 3). Although it has been proposed that over 60% of SEs are tissue- or cancer type-specific^23^, the top differential SE-associated genes were quite similar to those in other types of cancer^20,23,25,26^, highlighting the importance of these top gained SEs in carcinogenesis (Fig. 3b). We attempted to combine the absolute difference and FC data to successfully examine the highly reliable most altered SEs (Supplementary Figs. 12, 13), which might be a strategy worth recommending for other studies. The high overlap of gained and lost enhancers/SEs between ESCC and LNC (Figs. 1 and 3) validate the origin of metastatic LNC cells from primary ESCC and suggests the relative consistency of epigenomic features between primary and metastatic tumors, as well as the molecular consistency with other types of cancer (Supplementary Fig. 15).

Meanwhile, we recovered a series of potential tumor suppressors (EHF, MAL, and TFAP2B)^26^ that were associated with lost enhancers and could be uncovered only in normal tissues (Fig. 3c–e). We also identified a series of LNC-specific enhancers and SEs; however, we could not exclude the minor possibility of specific gain of a few enhancers reflected by a minimal number of immune cells. Actually, many gained enhancers in LNC tissues relative to EC, which were associated with oncogenes, were also gained in EC relative to normal tissues (Supplementary Fig. 13). Common gained/lost enhancers might be more reliable than LNC-specific altered enhancers. In support of this notion, (Fig. 3i, j), our survival analysis indicated that somatic gained SEs contribute to aberrant gene expression and predict a poor clinical prognosis.

Our epigenetic landscape contains an appealing pool of potential targets that can contribute to ESCC tumorigenesis and metastasis. We performed *trans*-element prediction using gained enhancers (Fig. 4) and integrated SEs with drug interaction databases to predict potential targets responsive to inhibitors (Fig. 5). By elucidating core transcriptional regulatory circuitries from the set of gained enhancers, we identified several key TFs, including RXRA, NFE2L2, IRF2, RELA, etc., involved in cell growth and migration (Fig. 4f, g), some of which were also revealed to have tumor-promoting functions in ependymoma and RCC^25,26^. Clarification of the potential roles of these TFs in establishing cancer-specific enhancer profiles will be of great importance. Consistent with this presumption, among TF circuitries predicted from gained enhancers, SOX2 has been previously identified as a factor of the core regulatory circuitry for controlling epigenetic and transcription patterns in ESCC cell lines^53^.

Our notable finding is the identification of HSP90AA1 and PDE4B as potent therapeutic targets, both of which have been previously proposed as potential biomarkers for ESCC^54–56^, further supporting our findings here. Importantly, the pharmacologic inhibition of these two molecules significantly suppressed cell proliferation, migration, and xenograft tumor growth (Fig. 5). PDE4B is a protective cyclic AMP-phosphodiesterase involved in heart failure and colon cancer^57,58^. Although the clinical relevance between PDE4B and ESCC is not as strong as that between HSP90AA1 and ESCC, PDE4B inhibitor exhibits very strong antitumor effects. Therefore, the mechanisms underlying the tumorigenic role of PDE4B remain to be elucidated in the future. HSP90AA1, a heat shock protein critical for the stability of its target proteins, is important for autophagy and drug resistance^59,60^. Here we validated its positive correlation with tumor progression and cancer mortality, and emphasized its role in ESCC carcinogenesis and metastasis.

In summary, we demonstrate that enhancer and SE deregulation attributes primarily to transcriptional reprogramming during carcinogenesis and metastasis and that LNC enhancer signatures are quite similar to that of primary ESCC but possess metastasis-specific features. By *trans*-element analysis from the set of altered enhancers, we successfully identified IRF2, RELA, NFATC2, etc., as essential TFs involved in cancer cell growth and migration, possibly via establishing oncogenic enhancer programs. SE-linked HSP90AA1 and PDE4B, emerge as crucial oncogenes and potential therapeutic targets for esophageal carcinogenesis.

## Methods

### ESCC patients

ESCC patients were recruited from the Department of Thoracic Surgery, Shanghai Chest Hospital Affiliated with Shanghai Jiaotong University, Shanghai, China. Tumor samples and clinical information were approved by the local Ethics Committee of Shanghai Chest Hospital Affiliated with Shanghai Jiaotong University. We complied with all relevant ethical regulations for this work with human participants. A total of 18 primary tumor samples from ESCC patients, including 15 males and three females aged from 49 to 74, were collected from November 2017 to April 2018, and written informed consent was obtained from all participants. All subjects received chemotherapy or radiotherapy before surgical therapy. These subjects were pre-diagnosed by biopsy with a high proportion of tumor cells and a squamous subtype. During surgery, fresh primary tumor tissues, lymph nodes, and adjacent normal tissues were collected and divided into four sections: one for cryosectioning and hematoxylin and eosin staining, one for storage in the sample bank, one for lysis with TRIzol reagents, and one for fixation with 1% formaldehyde for ChIP assays. The tumor sample purity was confirmed by hematoxylin and eosin staining^61^, with an estimated tumor cell content of at least 80%.

### Cell lines and reagents

Human HEK293T cells and TE1 cells were purchased from the Cell Bank (Shanghai Institutes for Biological Sciences, CAS, China). KYSE30 and KYSE150 cells were gifts from Dr. Zhihua Liu’s lab (Cancer Hospital Chinese Academy of Medical Science). All cells were tested without mycoplasma contamination. TE1 cells were authenticated by Cell Bank (Shanghai Institutes for Biological Sciences, CAS, China) using STR profiling. KYSE30 and KYSE150 cells were authenticated by Dr. Zhihua Liu’s lab using STR profiling. HEK293T cells were maintained in DMEM supplemented with 10% FBS (Gibco), and TE1, KYSE30, and KYSE150 cells were maintained in RPMI 1640 medium supplemented with 10% FBS (Gibco), 100 U/mL penicillin, and 100 μg/mL streptomycin at 37 °C in a humidified incubator with 5% CO2. Chemical inhibitors, including AH, palbociclib, endovion, SB297006, navitoclax, ML-030, hydroxyfasudil, and IT1t dihydrochloride (IT1t), were purchased from MCE, and the powders were dissolved according to the manufacturer’s instructions.

### Knockdown assay

shRNAs targeting TFs predicted from gained enhancers were constructed into the lentiviral vector pLVX-shRNA2 (Clontech), and an shRNA targeting luciferase coding genes was used as a control. shRNAs were designed with an online tool (https://www.sigmaaldrich.com/life-science/functional-genomics-andrnai/sirna/mission-predesigned-sirna.html), and two shRNAs were constructed for each TF. The shRNA sequences are provided in Supplementary Table 1. To generate viable lentiviruses, shRNA expressing vectors were co-transfected into HEK293T cells with the packaging vector delta-8.9 and VSVG using the calcium phosphate method. Cells infected with lentivirus expressing the shRNAs were subjected to quantitative real-time PCR analysis to assess knockdown efficiency and gene expression.

### Cell growth assay

Cells infected with lentivirus expressing indicated shRNAs or mock cells were plated into 24-well plates at 10,000 cells per well, and cell numbers were counted using a cell counter (Counter Star) every day until day 6. For chemical treatment, inhibitors were added to the medium 24 h after cells were seeded, and the cell number was determined at 72 h. Three wells were counted, and the average number of live cells was designated as a single replicate for each sample; each experiment was repeated three times.

### Cell apoptosis analysis

TE1 cells were plated into 24-well plates at 50,000 cells per well and treated with the indicated chemical drugs or DMSO after attachment. Cell apoptosis was detected using a Cell Cycle and Apoptosis Detection Kit (C1052; Beyotime Institute of Biotechnology, China). After cells were treated for three days, they were trypsinized, centrifuged, washed, and fixed with cold 70% ethanol at 4 °C overnight. Then, the fixation solution was removed, and the cells were incubated at 37 °C for 30 min in a solution containing propidium iodide and RNase A. Then the treated cells were analyzed using a FACScan flow cytometer (BDFACSCalibur, USA) and 10,000 cells were collected per sample. The data were analyzed using FlowJo 10.0 software (TreeStar, USA) with two or three replicates per sample, and each sample was analyzed independently three times.

### Wound healing assay

Mock TE1 cells or cells expressing indicated shRNAs were cultured in 12-well plates, and a wound was made in the confluent cell monolayer with 1000 µl pipette tip. Then, the wound width was monitored every 24 h using an inverted phase microscope. The relative cell migration rates were analyzed relative to the wound width at the time of initial wounding, with three replicates per sample.

### Xenograft experiments

Animal studies were conducted according to animal protocols approved by the local Ethics Committee of Shanghai Chest Hospital Affiliated with Shanghai Jiaotong University. We complied with all relevant ethical regulations for animal experiments. Male BALB/c nude mice (6 weeks old) were implanted with 1 × 10^6^ TE1 cells. The tumor volume was monitored every 2–3 days. The tumor volume was calculated as the length x width x width x 0.52; tumors were weighed after the mice were sacrificed on day 21. For evaluating the effects of AH and ML-030 on tumor growth, a total of 1 × 10^6^ cells were injected subcutaneously into nude mice (*n* = 5 for each group). Mice injected with TE1 cells were treated with AH (2.5 mg/kg every time) or ML-030 (2.5 mg/kg every time) every 3 days starting on day 2 after cell implantation. Tumor volumes were determined on days 4, 8, 14, and 21. On day 21, the tumors were collected from sacrificed mice for image acquisition and tumor weight determination. For detecting the metastasis of tumors in vivo, KYSE150 cells stably expressing firefly luciferase (Luc) cDNA was established by using lentiviruses. A total of, 1 × 10^5^ KYSE150/Luc cells were intramuscularly injected into the neck region to mimic esophagus cancer. Four weeks postinjection, in vivo luciferase imaging was conducted upon intraperitoneal injection of beetle luciferin (Promega, Madison, WI, USA) (0.2 ml of 15 mg/ml) using an IVIS Spectrum equipment (Caliper-PE). For administration of chemical inhibitor, ML-030 (2.5 mg/kg) or was intraperitoneally injected every 3 days starting from the day after tumor cell implantation.

### Survival analysis

Kaplan–Meier survival analysis was employed to analyze the overall survival probability with default parameters. To analyse survival related to altered SE-linked genes, because the maximum number of genes is limited with this tool, only the top altered SE-associated genes (gain: *PPL, SIM2, TRIOBP, VPS37B, THSD4, SLC7A1, AMOTL2, ZBTB7A, AHDC1, ZSWIM4, PHYHD1, PSCA, CCDC85C, PAQR7, ST3GAL4, EHF, TFAP2B, EPHA2, MINK1, RREB1, SMURF1, ESYT2, ZBTB7C, ATP8B1, RFX2, TMEM105, TMEM211, HOXA2, ATP5D*, and *WASF2*; loss: *RELT, IL4R, CCR3, MMP25, ADAM8, ADGRE5, THEMIS2, EPPK1, SLC7A5, NCF2, CD44, LDHA, CTSD, TMSB10, IER5L, FCGR3A, FCGR2A, SOCS3, NNMT, SPHK1, ITGA5, SIRPA, LMNA, DAD1, RPL35, ZC3HAV1, AHSA2, TBCD, IRX5*, and *HRAT92*) were used for survival analysis. Log-rank tests were applied to examine the significance of differences between Kaplan–Meier curves (Log-rank *P* < 0.05 was considered significant). GraphPad Prism 8 was used for analyzing the 2-year survival rate using our clinical data from 18 donors. For analysis of the correlation between ESCC and HSP90AA1, GEPIA (http://gepia.cancer-pku.cn/) was used for disease-free survival (DFS) analysis.

### Quantitative real-time PCR

Total RNA was isolated using TRIzol reagents (Invitrogen), and 1 µg of RNA was reverse transcribed using a Superscript III First-Strand Synthesis Kit (Vazyme, China). Then complementary DNA was diluted with ddH_2_O (1:10) and amplified using SYBRGreen PCR Master Mix (Vazyme, China). Relative gene expression levels were normalized to those of *GAPDH*. All relative quantification results were obtained from three independent experiments, and the primer sequences are listed in Supplementary Table 2.

### RNA-seq

Total RNA was extracted from fresh samples using TRIzol reagent. RNA-seq libraries were constructed using Stranded Total RNA Sample Prep Kit (Illumina, San Diego, CA) according to the manufacturer’s instructions, and 500 ng of total RNA was used for library construction using a TruSeq™ RNA Sample Prep Kit v2 (Illumina, San Diego, CA, USA) according to the manufacturer’s protocol. Sequencing was performed using the 150 bp pair-end read protocol (BerryGenomics, Beijing, China).

### ChIP-seq

ChIP-seq was performed as previously described^62,63^. Fresh tissues obtained during surgery were collected and digested into single cells or small cell aggregates, and ~1 × 10^5^ cells were fixed with 1% formaldehyde for 10 min in PBS at room temperature. Then, fixation was terminated with 125 nM glycine. After washing with cold PBS three times, the cells were centrifuged for storage at –80 °C. When a batch of samples was collected, the stored samples were subjected to ChIP experiments. Generally, cells were lysed in 100 µl of lysis buffer and sonicated for 15 cycles (30 s on, 60 s off; Diagenode Bioruptor). Then, 5% of the lysate was resolved for genomic DNA purification as input DNA, and 95 µl of the lysate was diluted to a volume of 1 mL. Through the ChIP experiments, samples were supplemented with a protease inhibitor. Then, 2 µg of an anti-H3K27ac antibody (Active Motif, 39133) was added to the diluted lysis buffer and mixed at 4 °C for 2 h. Protein G Dynabeads (Life Technologies; Thermofisher) were precleaned with wash buffer three times, and then a 20 µl volume of beads was added to the mixture and incubated overnight at 4 °C. Subsequently, the beads were washed with wash buffer five times and ChIP DNA was recovered for quantification. ChIP and input DNAs were subjected to library preparation with NEBNext ChIP-seq Library Pre Reagent (NEB, E7370S). Each library was sequenced to an average of 30 million raw reads on the X10 sequencing platform (BerryGenomics, Beijing, China).

### RNA-seq analysis

RNA-seq reads were aligned to the human genome (hg19) using STAR-2.6.1^64^ with the following parameters:–twopassMode Basic–sjdbOverhang 149–outFilterMultimapNmax 20–alignSJoverhangMin 8–alignSJDBoverhangMin 1–outFilterMismatchNmax 999–outFilterMismatchNoverLmax 0.1–alignIntronMin 20–alignIntronMax 500000–alignMatesGapMax 1000000–outFilterScoreMinOverLread 0.33–outFilterMatchNminOverLread 0.33–outSAMstrandField intronMotif–chimSegmentMin 15–chimJunctionOverhangMin 15–chimOutType WithinBAM SoftClip–chimMainSegmentMultNmax 1 and other default parameters. Transcript abundances at the gene level were calculated as TPM values using RSEM^65^. Transcripts with a count per million (cpm) of >1 in at least 18 samples were retained for subsequent analysis, and 16,173 genes passed this filtering criterion. The TPM values were further normalized through *Z*-score transformation when presented in heatmaps.

### Public data analysis

Independent public RNA-seq datasets of PDA (*n* = 340 samples; GSE71729) and CA (*n* = 246 samples; GSE41258) were downloaded from the NCBI Gene Expression Omnibus (GEO) database (https://www.ncbi.nlm.nih.gov/geo/)^66,67^. ChIP-seq data for eight crucial TFs were downloaded from ENCODE, and detailed information about these datasets is provided in Supplementary Data 20.

### ChIP-seq and analysis

ChIP-seq raw reads were mapped to the human reference genome (hg19) using Bowtie2 (version 2.3.4)^68^. Quality control for the aligned BAM files was performed with SAMtools^69^, enabling only uniquely mapped reads to be retained, and PCR duplicates were removed by Picard (“Picard Toolkit” 2019. Broad Institute, GitHub Repository. http://broadinstitute.github.io/picard/; Broad Institute) for subsequent analyses. Significant H3K27ac peaks were called by using MACS2 (2.1.1.20160309) with all default parameters except -p 1e-9 and -f BAMPE^70^. Bigwig files were generated from BAM files using deepTools^71^ with the following parameters: -binSize 50 -extendReads 200 and–normalizeUsing RPKM. The signal intensities of each ChIP library were scored against the corresponding input library, respectively. The input-subtracted peak signal within a region was measured as the RPKM value using bigWigAverageOverBed. The RPKM values were further normalized through *Z*-score transformation when presented in heatmaps.

### Epigenome roadmap datasets

Two publicly available H3K27ac peaks from normal esophageal tissues were downloaded from the NIH Roadmap Epigenomics Project Data (https://www.ncbi.nlm.nih.gov/geo/roadmap/epigenomics/) under accession numbers GSM906393 and GSM1013127. Similarities between the epigenome roadmap and our H3K27ac ChIP-seq data were assessed by determining the percentage of overlapping peaks relative to our H3K27ac peaks using BEDtools^72^.

### Differential gene expression analysis

Differential gene expression analysis between groups was performed with the DESeq2 R package^73,74^. Genes with an FC value of >1.5 and a false discovery rate (FDR)-adjusted *P* value of <0.05 were considered to be significantly differentially expressed.

### PCA

PCA was performed on the altered promoter elements or enhancers or all SEs using the first two PCs of the H3K27ac signals, and the cumulative variance and the proportion of variance accounted for each PC were computed using the FactorMineR R package^75^.

### Enhancer, promoter, and SE analysis

We defined active promoter elements as peaks located completely within ±2.0 kb of TSSs, which were excluded from enhancers. Furthermore, the H3K27ac peaks were merged into integrated but nonoverlapping peak sets across all samples (both normal and tumor samples). Quantile-normalization was performed using the R package preprocessCore (preprocessCore: A collection of preprocessing functions. R package version 1.44.0. https://github.com/bmbolstad/preprocessCore) to reduce potential batch effects. SE regions were identified using Rank Ordering of Super-Enhancers (ROSE)^33^ from merged enhancers or individual enhancers from each sample with default parameters.

### Identification of differential enhancers

Differential analysis was performed to identify altered elements. Customized criteria —a FC of ≥2 and an absolute difference of ≥0.5 in the H3K27ac RPKM value in ≥6/10 patients (≥6/8 for LNC comparisons), were applied to identify gained or lost regions. The track of differential regions were visualized using Integrative Genomics Viewer (IGV)^76^.

### Saturation analysis

Saturation analysis was performed for all enhancers and promoter elements. Specifically, the possible frequency of enriched regions in all samples (*n* = 1–28) was stated, and as the number of samples increased, the number of identified regions tended to be stable. The same methods were used to analyze altered enhancers and promoter elements.

### Motif analysis

To detect enriched sequence motifs in altered enhancers, we performed motif analysis using HOMER^39^ with default parameters. Predicted TF binding sites with at least 60% of overlap with altered enhancers were counted, and the ranks of the most highly enriched TFs are presented according to their *P* value.

### Core transcriptional regulatory circuitry analysis

All TFs predicted from common gained and LNC-specific gained enhancers were subjected to core circuitry analysis by CRCmapper to computationally infer TF connectivity using^41^ default parameters. The final connectivity scores were normalized to 0–100.

### Identification of regulatory networks for altered enhancer-associated TFs

TFs predicted from common gained, common lost, and LNC-specific gained enhancers were subjected to TF regulatory network analysis. To construct EC-, LNC- and Nor-specific TF regulatory networks, we selected the top enriched TFs in each subgroup and calculated the Spearman correlation coefficient for each TF-TF pair. Genes with a high degree are presented, and the resulting networks highlight the TF for functional validation. Networks were visualized using Cytoscape v3.6.1^77^.

### TAD analysis

We accessed publicly available topology-associated domains (TADs) previously obtained in IMR90 cells [ENCFF307RGV]. The Hi-C maps of regions around the TADs covering the *ANO1*, *HSP90AA1*, and *PMEPA1* genes were constructed using Juicebox v1.11.08^78^. Enrichment of H3K27ac within the same regions is presented in snapshots from IGV files. In addition, the predicted TFs with potential binding sites within these regions are presented using HOMER.

### GO and KEGG pathway analyses

We performed GO enrichment analysis to identify key terms enriched in genes associated with recurrent enhancers or predicted SEs http://geneontology.org/ and performed KEGG pathway analysis using DAVID (http://david.abcc.ncifcrf.gov/home.jsp). The terms and pathways with an adjusted *P* value < 0.05 were considered significantly enriched.

### Identification of drug–gene interactions linked to SEs

Common gained and LNC-specific gained super-enhancer-associated genes were used to query the Washington University Drug Gene Interaction database (http://dgidb.org/search_interactions); the interactions between genes and drugs are presented in Supplementary Data 21; these interactions were used to identify druggable gene targets^25^.

### Statistics and reproducibility

The results were obtained from three independent experiments and are presented as the mean ± s.d. values. For analyzing relative expression levels as FPKM values, the FPKM values from RNA-seq data were used and are presented as the mean ± s.d. values. All original data presented in the main figures or supplemental figures are provided in the Source Data file. Each treatment was performed in triplicate, and the values for the individual treatments were averaged and normalized to those for the control group. Statistical analyses and plotting were carried out using GraphPad Prism 8.0. Comparisons of mean values were performed with Student’s unpaired *t*-test. Differences were considered significant when *P < 0.05, **P < 0.01, or ***P < 0.001 without statements. For analyzing differences between gained and lost enhancers or linked genes, the cut-off values are presented within the figures. For cell growth, wound healing rate, and apoptosis analysis, two or three independent experiments were performed, and similar results were obtained.

### Reporting Summary

Further information on research design is available in the Nature Research Reporting Summary linked to this article.

## Supplementary information

Supplementary Information

Description of Additional Supplementary Files

Supplementary Data 1

Supplementary Data 2

Supplementary Data 3

Supplementary Data 4

Supplementary Data 5

Supplementary Data 6

Supplementary Data 7

Supplementary Data 8

Supplementary Data 9

Supplementary Data 10

Supplementary Data 11

Supplementary Data 12

Supplementary Data 13

Supplementary Data 14

Supplementary Data 15

Supplementary Data 16

Supplementary Data 17

Supplementary Data 18

Supplementary Data 19

Supplementary Data 20

Supplementary Data 21

Reporting Summary

## Data Availability

The ChIP-seq and RNA-seq data generated in this study have been deposited in the Sequence Read Archive (SRA) database under accession code PRJNA665151 and PRJNA665149. Published RNA-seq datasets in PDA and CA were obtained from the Gene Expression Omnibus (GEO) database “GSE71729” and “GSE41258”^66,67^, respectively. Two publicly available H3K27ac ChIP-Seq peaks in normal esophageal tissues were downloaded from the NIH Roadmap Epigenomics Project Data (and “GSM1013127”). Processed Hi–C interactions in IMR90 cells was obtained from ENCODE “ENCFF307RGV”. Other public TFs RXRA, NFE2L2, ZNF519, ESRRA, RELA, ETS1, IRF2, and ZEB1 ChIP-Seq datasets in HepG2, HEK293, and GM12878 were obtained from ENCODE (“ENCSR500WXT”, “ENCSR488EES”, “ENCSR754SOI”, “ENCSR000EEW”, “ENCSR000EAG”, “ENCSR681WHQ”, “ENCSR604UJV”, and “ENCSR000BND”). And file names of the used ChIP-seq data for TFs were detailed in Supplementary Data 20. Source data are provided with this paper. The remaining data are available within the article, Supplementary Information, Supplementary Data files, or Source Data file. Source data are provided with this paper.

## Source data

Source Data
