# Supplementary material for: Oncogenic enhancers drive esophageal squamous cell carcinogenesis and metastasis
Source: Nat Commun. 2021 Jul 22;12:4457. doi: 10.1038/s41467-021-24813-2 (PMC8298514; doi:10.1038/s41467-021-24813-2)
Supplement: Supplementary file 1 — Supplementary Information [file 41467_2021_24813_MOESM1_ESM.pdf]

a

No.	Type	Tumor Size (cm)	Stage	LNC location	RNA-seq	ChIP-seq
2	ESCC	7.5×4×3	IV	Mid-paraesophageal; Right recurrent laryngeal nerve; Para-Cardia	√	-
3	ESCC	7×5×1.5	III	Paragastric; Mid-paraesophageal	√	-
4	ESCC	9×5×3	IV	Right recurrent laryngeal nerve	√	-
5	ESCC	4.5×3.5×1.5	III	Left recurrent laryngeal nerve	√	-
6	ESCC	5.6×3.8×2.7	IV	Right/left recurrent laryngeal nerve	√	-
7	ESCC	2.2×2.1×1.8	III	-	√ (no LNC)	√ (no LNC)
8	ESCC	3×2×1.5	III	Left recurrent laryngeal nerve	√	-
9	ESCC	3×3×2.5	III	-	√(no LNC)	-
10	ESCC	4.2×3×1.5	IV	Paragastric; Lower-paraesophageal; Left recurrent laryngeal nerve; Left principal bronchus	√	√
11	ESCC	3×1.5×0.3	III	Left/right recurrent laryngeal nerve; Left supraclavicular	√	√
12	ESCC	5×3.5×1.5	IV	Upper/mid/lower-paraesophageal; Left recurrent laryngeal nerve; Left principal bronchus	√	√
13	ESCC	2.4×2×1.3	III	Left recurrent laryngeal nerve; Left/right principal bronchus	√	√
14	ESCC	8.5×3×1.5	IV	Right/left recurrent laryngeal nerve; Left/right principal bronchus; Upper-paraesophageal; Para-thoracic duct	√	√
17	ESCC	5×3×2	III	-	√ (no LNC)	√ (no LNC)
18	ESCC	2.5×2×1.2	III	Right recurrent laryngeal nerve	√ (no LNC)	-
19	ESCC	5.5×3.5×1.5	IV	Right recurrent laryngeal nerve	√	√
20	ESCC	4.5×4.4×3	IV	Upper/lower-paraesophageal; Right recurrent laryngeal nerve	√	√
21	ESCC	3.6×2.5×1.1	III	Upper-paraesophageal; Right recurrent laryngeal nerve	√	√

b

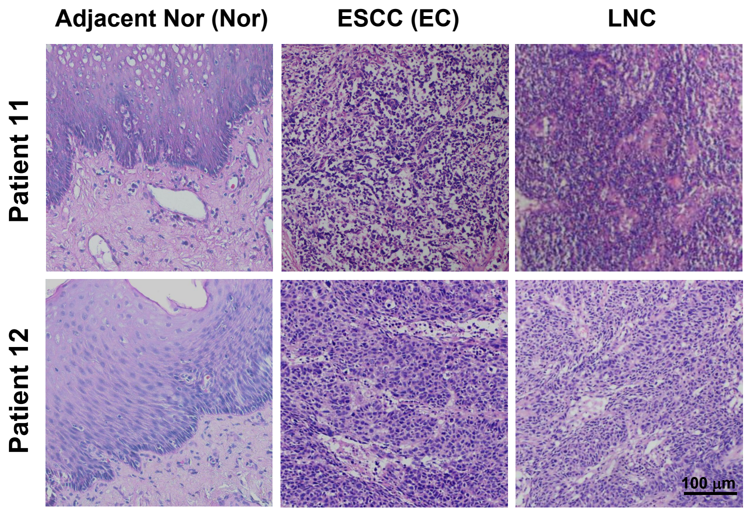

Supplementary Figure 1. The clinical data for esophageal cancer patients.

a. The clinical data for cancer patients in the present study. All patients were numbered, and No. 1, 15, and 16 patients were excluded because of low tumor cell purity in pathological detection.

b. Clinical H&E staining results for two representative patients.

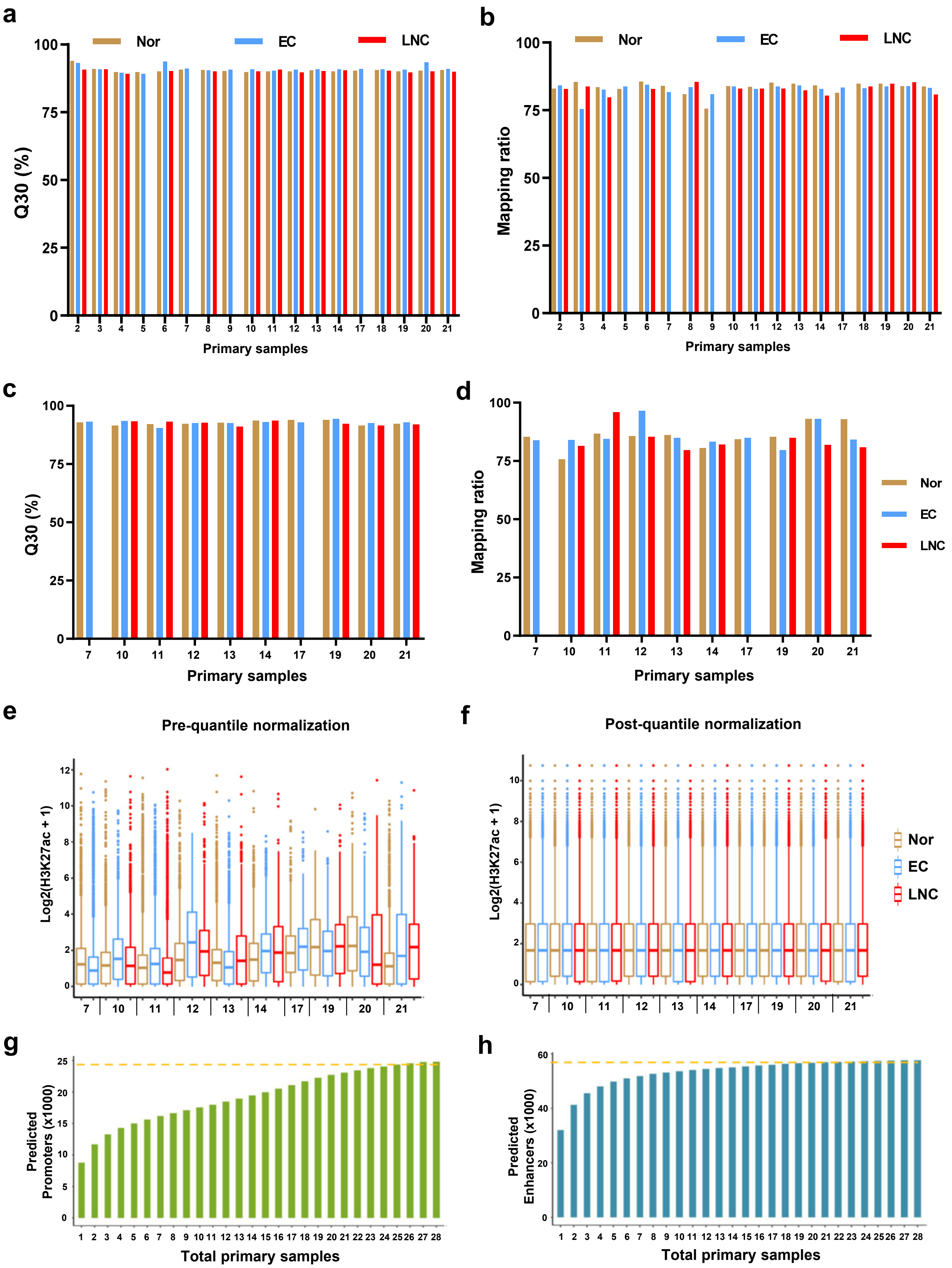
a
Nor
EC
LNC
Q30 (%)
100
75
50
25
0
2 3 4 5 6 7 8 9 10 11 12 13 14 17 18 19 20 21
Primary samples
b
Nor
EC
LNC
Mapping ratio
100
75
50
25
0
2 3 4 5 6 7 8 9 10 11 12 13 14 17 18 19 20 21
Primary samples
c
Q30 (%)
100
75
50
25
0
7 10 11 12 13 14 17 19 20 21
Primary samples
d
Mapping ratio
100
75
50
25
0
7 10 11 12 13 14 17 19 20 21
Primary samples
Nor
EC
LNC
e
Pre-quantile normalization
Log2(H3K27ac + 1)
12
10
8
6
4
2
0
7 10 11 12 13 14 17 19 20 21
f
Post-quantile normalization
Log2(H3K27ac + 1)
10
8
6
4
2
0
7 10 11 12 13 14 17 19 20 21
Nor
EC
LNC
g
Predicted Promoters (x1000)
25
20
15
10
5
0
1 2 3 4 5 6 7 8 9 10 11 12 13 14 15 16 17 18 19 20 21 22 23 24 25 26 27 28
Total primary samples
h
Predicted Enhancers (x1000)
60
40
20
0
1 2 3 4 5 6 7 8 9 10 11 12 13 14 15 16 17 18 19 20 21 22 23 24 25 26 27 28
Total primary samples

Supplementary Figure 2. ChIP-seq and RNA-seq data quality control.

a. Percentage of RNA-seq reads for which all bases have scores > Q30 (this is equivalent to the base-call accuracy = 99.9%).

b. Mapping ratio for RNA-seq data.

c. Percentage of ChIP-seq reads for which all bases have scores > Q30.

d. Mapping ratio for ChIP-seq data.

e. Box plots of H3K27ac enhancer profiles (57,675 enhancer loci evaluated per sample) before quantile normalization for Nor, EC, and LNC samples (28 independent samples).

f. Box plots of H3K27ac enhancers after quantile normalization. e-f, Box plots are shown with the center (median), upper, and lower quartile range per sample, and thick lines indicate the median values. The whiskers indicate 1.5 IQR and the points indicates values that are bigger than 1.5 IQR.

g-h. Saturation analysis shows the numbers of predicted promoter elements (g) and enhancers (h) across increasing number of primary samples. The yellow dotted lines indicate the total number of predicted regulatory elements by integrating 28 ChIP-seq profiles. Source data are provided as a Source Data file.

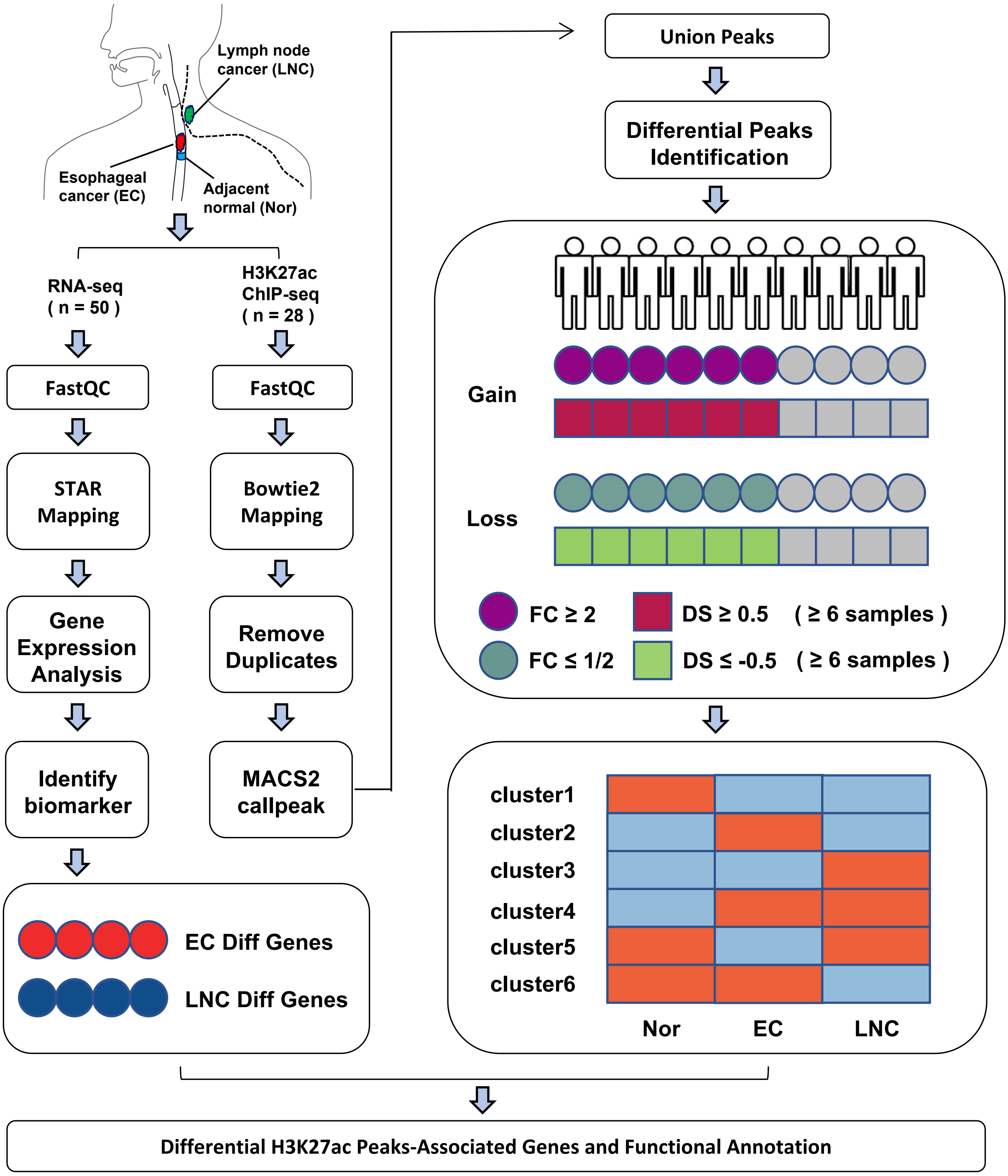

Lymph node cancer (LNC)
Esophageal cancer (EC)
Adjacent normal (Nor)
RNA-seq (n = 50)
H3K27ac ChIP-seq (n = 28)
FastQC
FastQC
STAR Mapping
Bowtie2 Mapping
Gene Expression Analysis
Remove Duplicates
Identify biomarker
MACS2 callpeak
EC Diff Genes
LNC Diff Genes
Union Peaks
Differential Peaks Identification
Gain
Loss
FC ≥ 2
DS ≥ 0.5
(≥ 6 samples)
FC ≤ 1/2
DS ≤ -0.5
(≥ 6 samples)
cluster1
cluster2
cluster3
cluster4
cluster5
cluster6
Nor
EC
LNC
Differential H3K27ac Peaks-Associated Genes and Functional Annotation

Supplementary Figure 3. Diagram for experimental and bioinformatic procedures. Briefly, 50 RNA-seq profiles were generated from 18 paired patients, and 28 H3K27ac ChIP-seq profiles were generated from 10 paired patients. After regular quality control and processing, RNA-seq data were subjected to gene expression analysis, Differential Expression Gene Analysis (EC Diff genes: EC vs. Nor; LNC Diff genes: LNC vs. Nor), and biomarker analysis; ChIP-seq data were subjected to differential peak identification (promoter elements and enhancers), including *cis*-regulatory element gain and loss between two groups of samples (EC vs. Nor, LNC vs. Nor, and LNC vs.EC). Then, these differential enhancers were subjected to unsupervised hierarchical clustering, and 6 clusters (G1-G6) of differential enhancers were generated (as shown in Figure 1b). Diff, differential; FC, fold change. DS, differential signal.

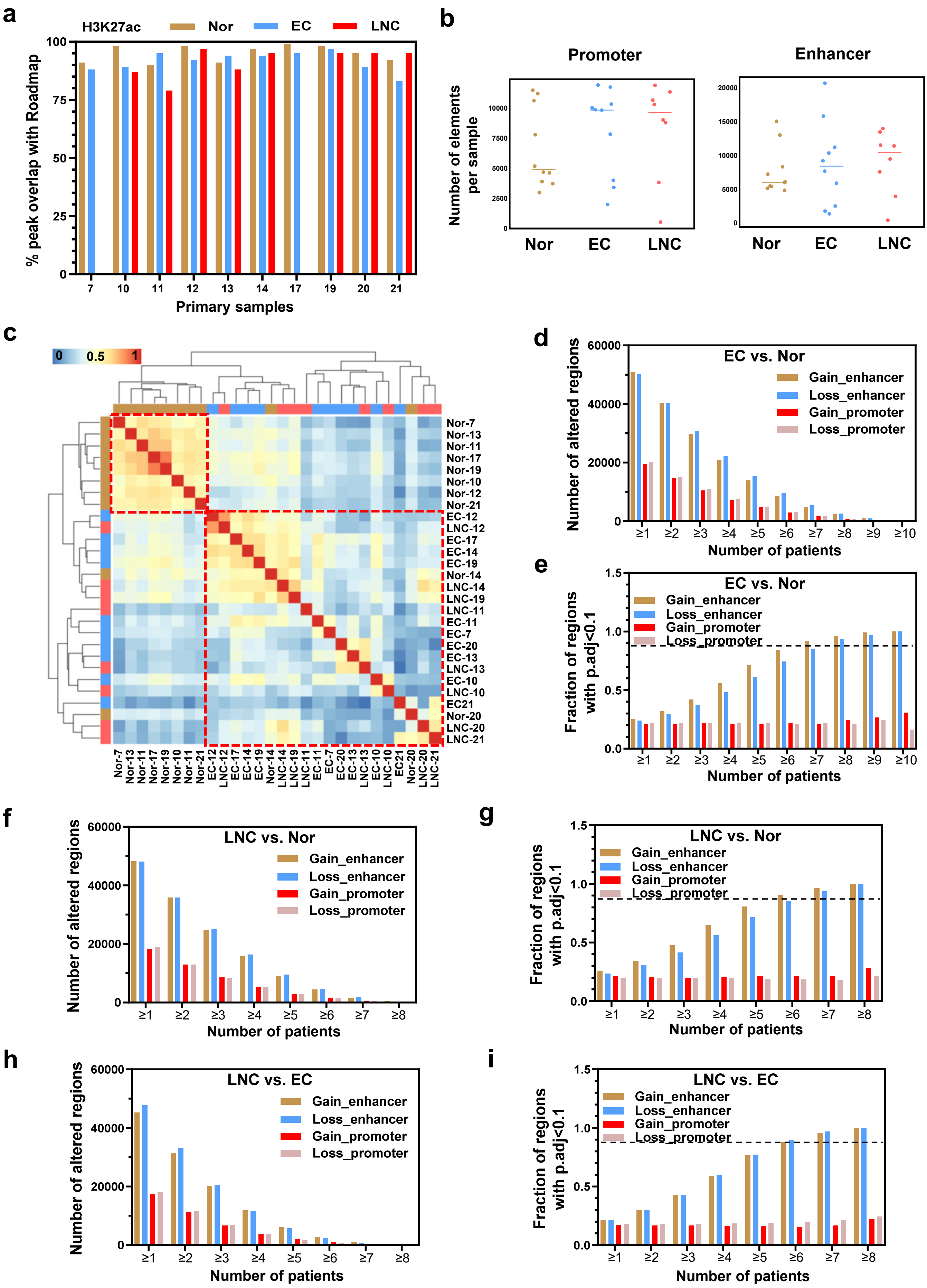
a
H3K27ac
Nor
EC
LNC
% peak overlap with Roadmap
100
75
50
25
0
7
10
11
12
13
14
17
19
20
21
Primary samples
b
Promoter
Enhancer
Number of elements per sample
10000
7500
5000
2500
0
20000
15000
10000
5000
0
Nor
EC
LNC
c
0
0.5
1
Nor-7
Nor-13
Nor-11
Nor-17
Nor-19
Nor-10
Nor-12
Nor-21
EC-12
LNC-12
EC-17
EC-14
EC-19
Nor-14
LNC-14
LNC-19
LNC-11
EC-11
EC-7
EC-20
EC-13
LNC-13
EC-10
LNC-10
EC21
Nor-20
LNC-20
LNC-21
d
EC vs. Nor
Gain_enhancer
Loss_enhancer
Gain_promoter
Loss_promoter
Number of altered regions
60000
40000
20000
0
≥1
≥2
≥3
≥4
≥5
≥6
≥7
≥8
≥9
≥10
Number of patients
e
EC vs. Nor
Fraction of regions with p.adj<0.1
1.5
1.0
0.5
0.0
f
LNC vs. Nor
g
LNC vs. Nor
h
LNC vs. EC
i
LNC vs. EC

Supplementary Figure 4. Enhancer supporting data.

a. Percentage of H3K27ac peaks across 28 profiles overlapping with H3K27ac peaks from normal esophageal tissues (Epigenome Roadmap; GSM1013127 and GSM906393).

b. Number of promoter elements and enhancers across 28 H3K27ac profiles. Lines indicate the mean values.

c. Unsupervised hierarchical clustering of all enhancers with H3K27ac signal across Nor, EC, and LNC samples. Nor can be generally separated from EC and LNC.

d-i. Number of altered promoter elements and enhancers at different cut-offs of recurrence for different comparisons (d: EC vs. Nor; f: LNC vs. Nor; h: LNC vs.EC). The fraction of altered elements (e, g, i) that meet statistical significance defined by paired *t*-tests (one-tailed) with Benjamini-Hochberg correction ($p.adj < 0.1$) at different cut-offs of recurrence. At the threshold of ≥ 6/10 patients, 80% of the altered regions achieved statistical significance. Source data are provided as a Source Data file.

a

Regions	Definition	Total	Relative to Nor				Relative to EC	
			Recurrently gained in EC	Recurrently lost in EC	Recurrently gained in LNC	Recurrently lost in LNC	Recurrently gained in LNC	Recurrently lost in LNC
**Promoter**	H3K27ac+/ ±2 kb TSS	24,823	2,917	3,027	1,471	1,403	864	715
**Enhancer**	H3K27ac+/ promoter-	57,675	8,587	9,642	4,399	4,624	2,733	2,466

***p* < 1e-9**

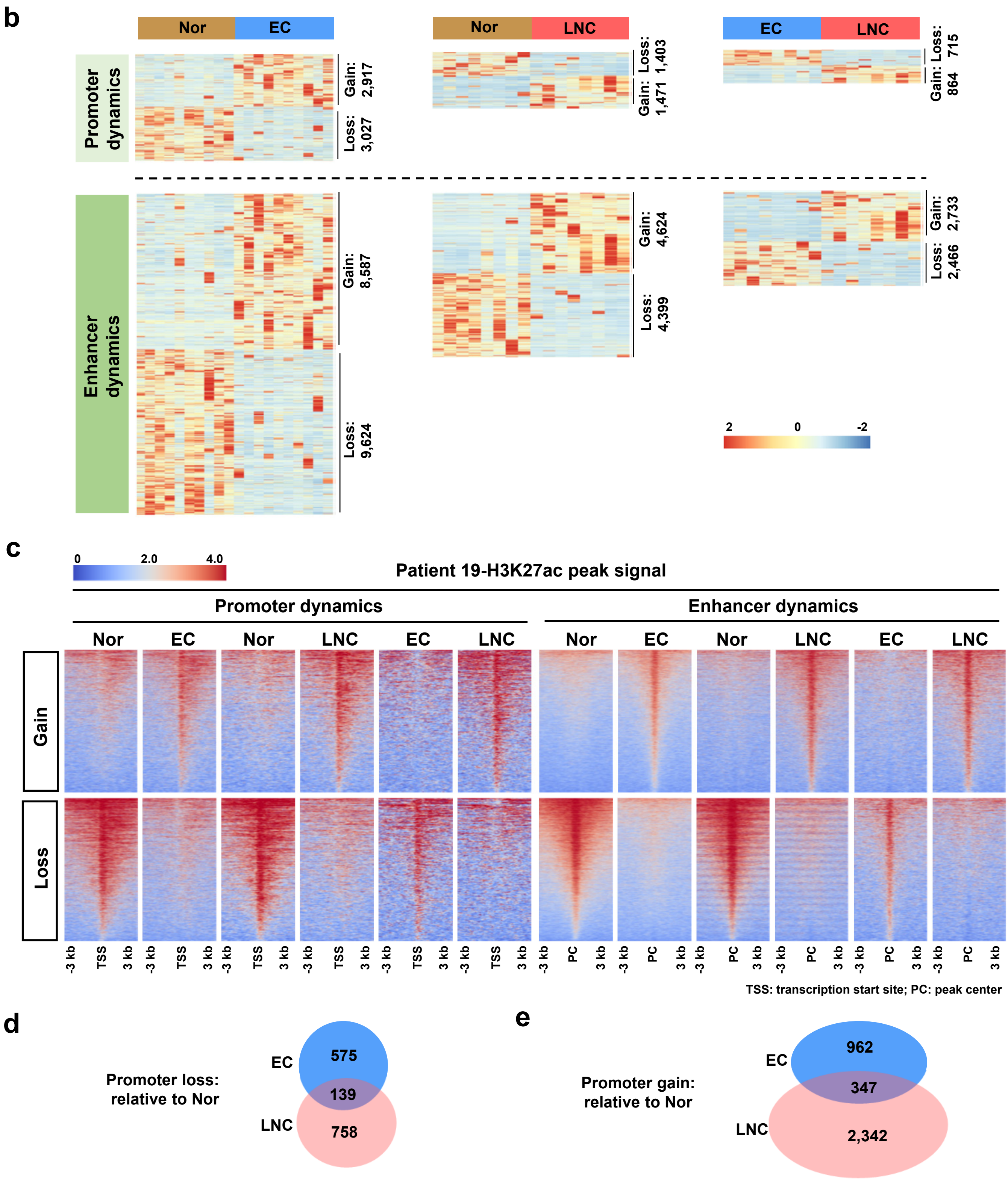

Supplementary Figure 5. Differential promoter and enhancer analysis.

a. Number of the total, recurrently gained, and recurrently lost promoter elements and enhancers in EC or LNC relative to Nor, as well as number of recurrently gained and lost promoter elements and enhancers in LNC relative to EC. Putative active promoter elements were defined by H3K27ac occupancy and proximity to TSS within 2 kb. Putative active enhancers are defined by the presence of H3K27ac exclusive with promoters.

b. Heatmap showing the gained and lost promoter elements and enhancers listed in a.

c. Representative heat maps showed H3K27ac levels of altered promoter elements and enhancers in a set of paired samples from a representative patient (patient 19: EC vs. Nor, LNC vs. EC, and LNC vs.EC). Each line represents a peak and neighboring 6 kb. TSSs were shown as the center for promoter dynamics and peak centers were shown as the centers of altered enhancer regions.

d-e. Veen diagram showing the overlapping number of lost (d) or gained (e) promoter elements relative to Nor.

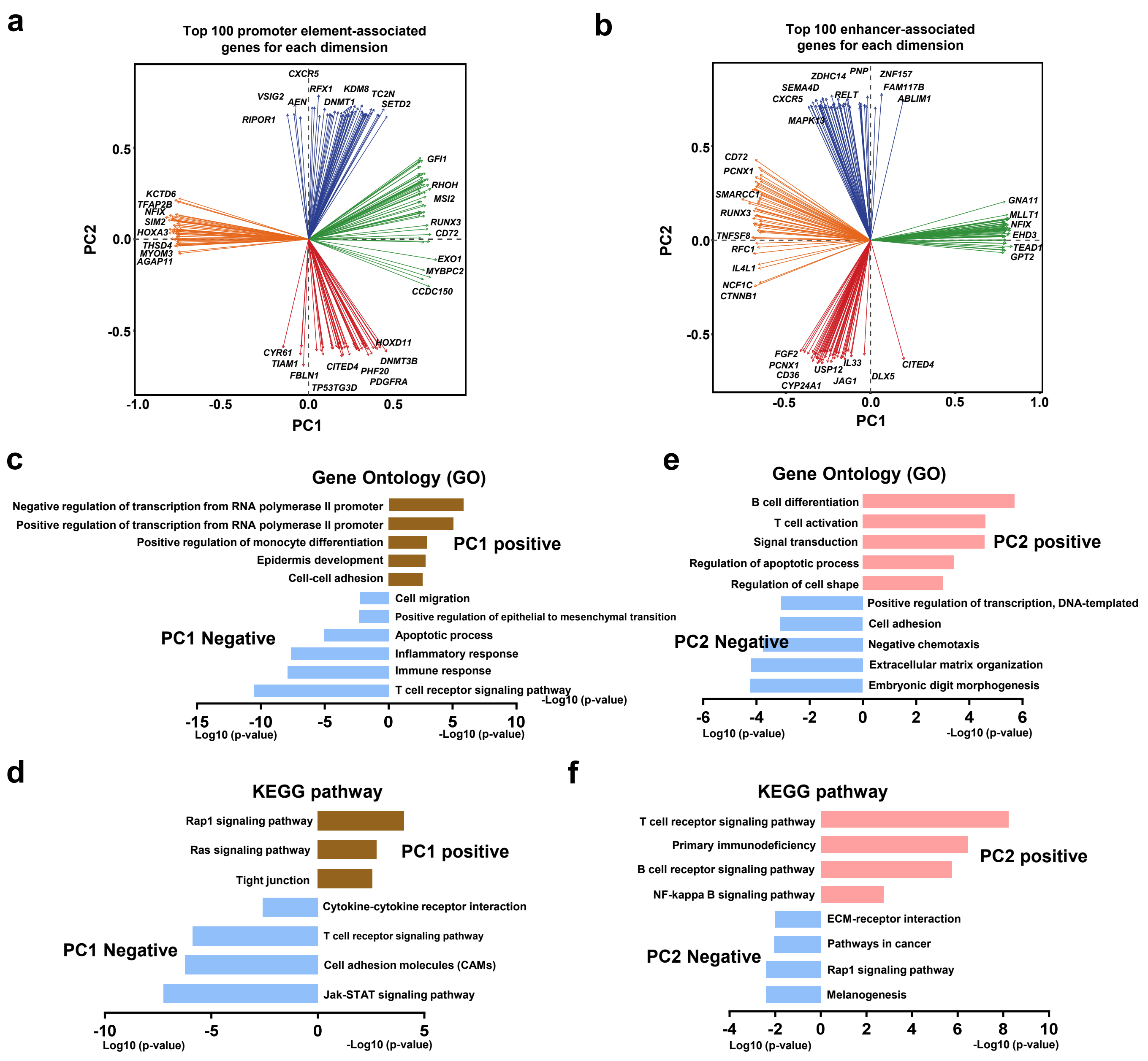

g

Regions	Definition	Total	Relative to Nor				Relative to EC	
			Recurrently gained in EC	Recurrently lost in EC	Recurrently gained in LNC	Recurrently lost in LNC	Recurrently gained in LNC	Recurrently lost in LNC
Super enhancer	H3K27ac+/ promoter-	1,042	436	481	427	310	437	217

Identification of SEs for each group based on the mean signal:
SE gain: the average diff_signal of all samples > 0, and gained in 5/10 (8)
SE loss: the average diff_signal of all samples < 0, and lost in 5/10 (8)

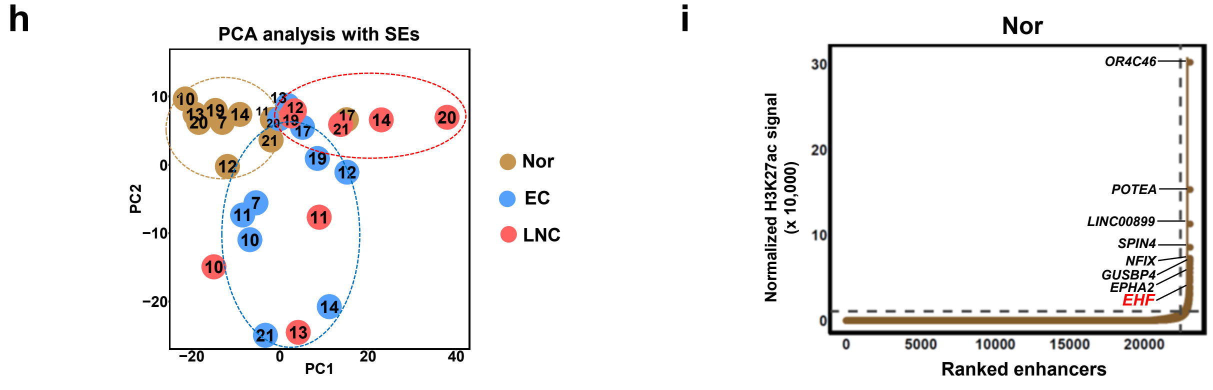

Supplementary Figure 6. Supplemental data for analysis of enhancers and super-enhancers.

a. The top 100 (50 positive and 50 negative) promoter element-associated genes for each dimension and highlighted genes were presented in PCA analysis.

b. The top 100 (50 positive and 50 negative) enhancer-associated genes for each dimension and highlighted genes were presented in PCA analysis.

c-f. The top 1,000 enhancer-associated genes for each dimension (PC1 positive, PC1 negative, PC2 positive, and PC2 negative) were subjected to Gene Ontology (GO) (c and e) and KEGG pathway (d and f) analysis.

g. Number of the total, recurrently gained, lost super-enhancers in EC or LNC relative to Nor, as well as number of recurrently gained and lost super-enhancers in LNC relative to EC. Super-enhancers (SEs) were identified for each group based on the mean signals. The gained SEs were defined according to the criteria: the average difference signal of all samples > 0, and absolute difference > 0 in at least 5 patients (gained in 5/10 or 5/8); the lost SEs were defined according to the criteria: the average difference signal of all samples < 0, and absolute difference < 0 in at least 5 patients (lost in 5/10 or 5/8).

h. PCA analysis of 28 Nor, EC, and LNC samples using all super-enhancers.

i. Identification of super-enhancers in Nor samples and top super-enhancer-associated genes. Source data are provided as a Source Data file.

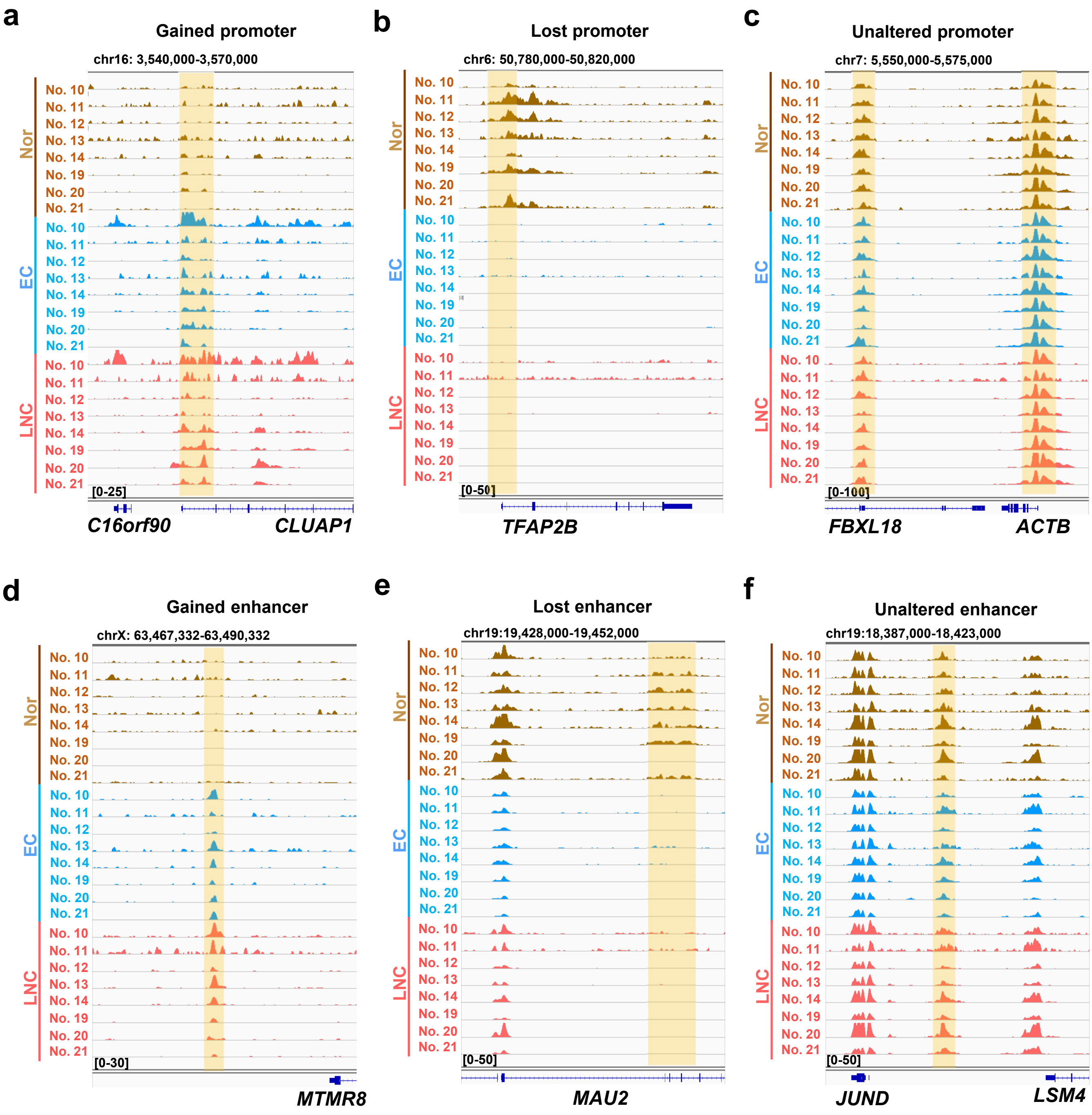
a
Gained promoter
chr16: 3,540,000-3,570,000
Nor
EC
LNC
No. 10
No. 11
No. 12
No. 13
No. 14
No. 19
No. 20
No. 21
[0-25]
C16orf90
CLUAP1
b
Lost promoter
chr6: 50,780,000-50,820,000
[0-50]
TFAP2B
c
Unaltered promoter
chr7: 5,550,000-5,575,000
[0-100]
FBXL18
ACTB
d
Gained enhancer
chrX: 63,467,332-63,490,332
[0-30]
MTMR8
e
Lost enhancer
chr19:19,428,000-19,452,000
[0-50]
MAU2
f
Unaltered enhancer
chr19:18,387,000-18,423,000
[0-50]
JUND
LSM4

Supplementary Figure 7. Examples for altered elements.

a-f. Examples for gained promoter elements (c), lost promoter elements (b), gained enhancers (d), and lost enhancers (e). Randomly selected unaltered promoters (c) and enhancers (f) served as a control. The altered or unaltered regions were highlighted in yellow, and H3K27ac tracks were presented using normalized H3K27ac enrichment data.

a

	G1	G2	G3	G4	G5	G6
**Peak number**	985	837	1974	871	548	2715
**Annotated gene number**	868	733	1573	758	514	1841
**Expressed gene number**	574	524	1146	582	383	1389
**Differentially expressed genes (DEGs)**	237	247	524	253	177	624
**DEGs matched to H3K27ac pattern**	71	82	413	51	9	442

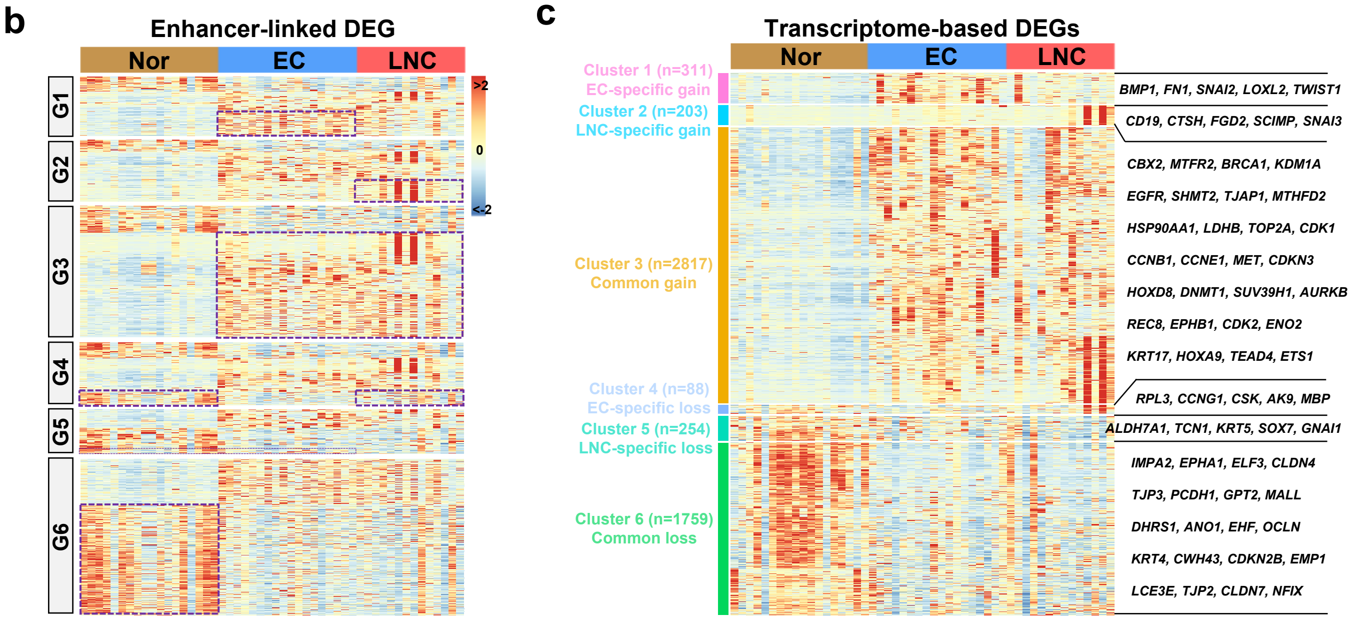

Supplementary Figure 8. Supplemental data for analysis of enhancers and super-enhancers.

a. The expression of differential enhancer (G1-G6; Figure 1b)-associated genes in all samples were determined, and the number of peaks, annotated genes, and expressed genes was presented. The expressed genes (count per million (CPM) > 1 in at least 18 sample) were subjected to DEG analysis between two of three groups, and the number of DEGs was also presented. Among these DEGs, the genes with positively matched H3K27ac enrichment patterns were numbered.

b. Heatmap showing the expression of expressed genes associated with differential enhancers (G1-G6). The genes matched with H3K27ac enrichment patterns were highlighted with purple boxes.

c. The expression of DEGs analyzed from 50 transcriptomes. Representative genes were presented at the right panel. Source data are provided as a Source Data file.

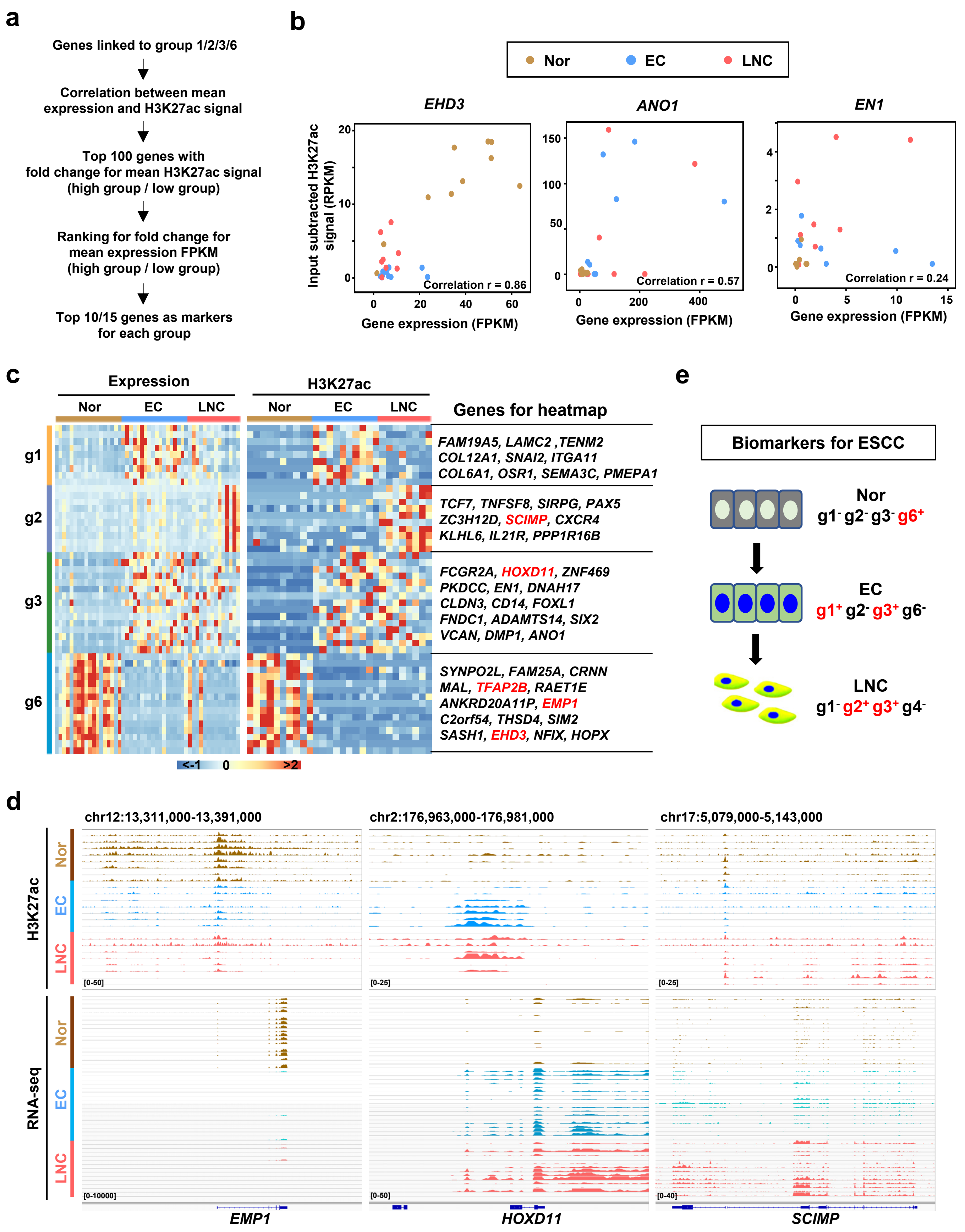

a
Genes linked to group 1/2/3/6
Correlation between mean expression and H3K27ac signal
Top 100 genes with fold change for mean H3K27ac signal (high group / low group)
Ranking for fold change for mean expression FPKM (high group / low group)
Top 10/15 genes as markers for each group
b
Nor
EC
LNC
EHD3
ANO1
EN1
Input subtracted H3K27ac signal (RPKM)
Correlation r = 0.86
Correlation r = 0.57
Correlation r = 0.24
Gene expression (FPKM)
c
Expression
H3K27ac
Genes for heatmap
g1
g2
g3
g6
FAM19A5, LAMC2 ,TENM2
COL12A1, SNAI2, ITGA11
COL6A1, OSR1, SEMA3C, PMEPA1
TCF7, TNFSF8, SIRPG, PAX5
ZC3H12D, SCIMP, CXCR4
KLHL6, IL21R, PPP1R16B
FCGR2A, HOXD11, ZNF469
PKDCC, EN1, DNAH17
CLDN3, CD14, FOXL1
FNDC1, ADAMTS14, SIX2
VCAN, DMP1, ANO1
SYNPO2L, FAM25A, CRNN
MAL, TFAP2B, RAET1E
ANKRD20A11P, EMP1
C2orf54, THSD4, SIM2
SASH1, EHD3, NFIX, HOPX
<-1 0 >2
e
Biomarkers for ESCC
Nor
g1- g2- g3- g6+
EC
g1+ g2- g3+ g6-
LNC
g1- g2+ g3+ g4-
d
chr12:13,311,000-13,391,000
chr2:176,963,000-176,981,000
chr17:5,079,000-5,143,000
H3K27ac
RNA-seq
[0-50]
[0-25]
[0-25]
[0-10000]
[0-50]
[0-40]
EMP1
HOXD11
SCIMP

Supplementary Figure 9. Supplemental data for analysis of enhancers and super-enhancers.

a. The pipeline for identifying biomarkers for three groups of samples. Briefly, genes linked to differential enhancers (G1, G2, G3, and G6) were subjected to Pearson correlation analysis with its nearest H3K27ac peak signals in 28 profiles. Genes with positive correlation coefficient were selected out for subsequent analysis. The top 100 genes with most differential H3K27ac enhancers calculated from the ratio of mean H3K27ac peak signals between high group over low group were collected; then these genes were ranked by mRNA expression fold changes of mean expression in TPM (high group / low group). Finally, the top 10/15 genes were designated as biomarkers for G1, G2, G3, and G6 separately.

b. Examples for the correlation between gene expression (TPM) and nearest H3K27ac enrichment signals (input subtracted; RPKM). Correlation coefficient r was presented.

c. Heatmap showing expression of identified biomarkers for g1 (EC-specific high), g2 (LNC-specific high), g3 (common gained), and g6 (common lost). The nearest H3K27ac peak density was shown in the middle panel and the gene names were listed in the right panel.

d. Representative tracks of H3K27ac ChIP-seq and RNA-seq at the *EMP1*, *HOXD11*, and *SCIMP* loci across Nor, EC, and LNC samples in 10 patients.

e. Diagram showing biomarker combinations for discriminating Nor, EC, and LNC. Source data are provided as a Source Data file.

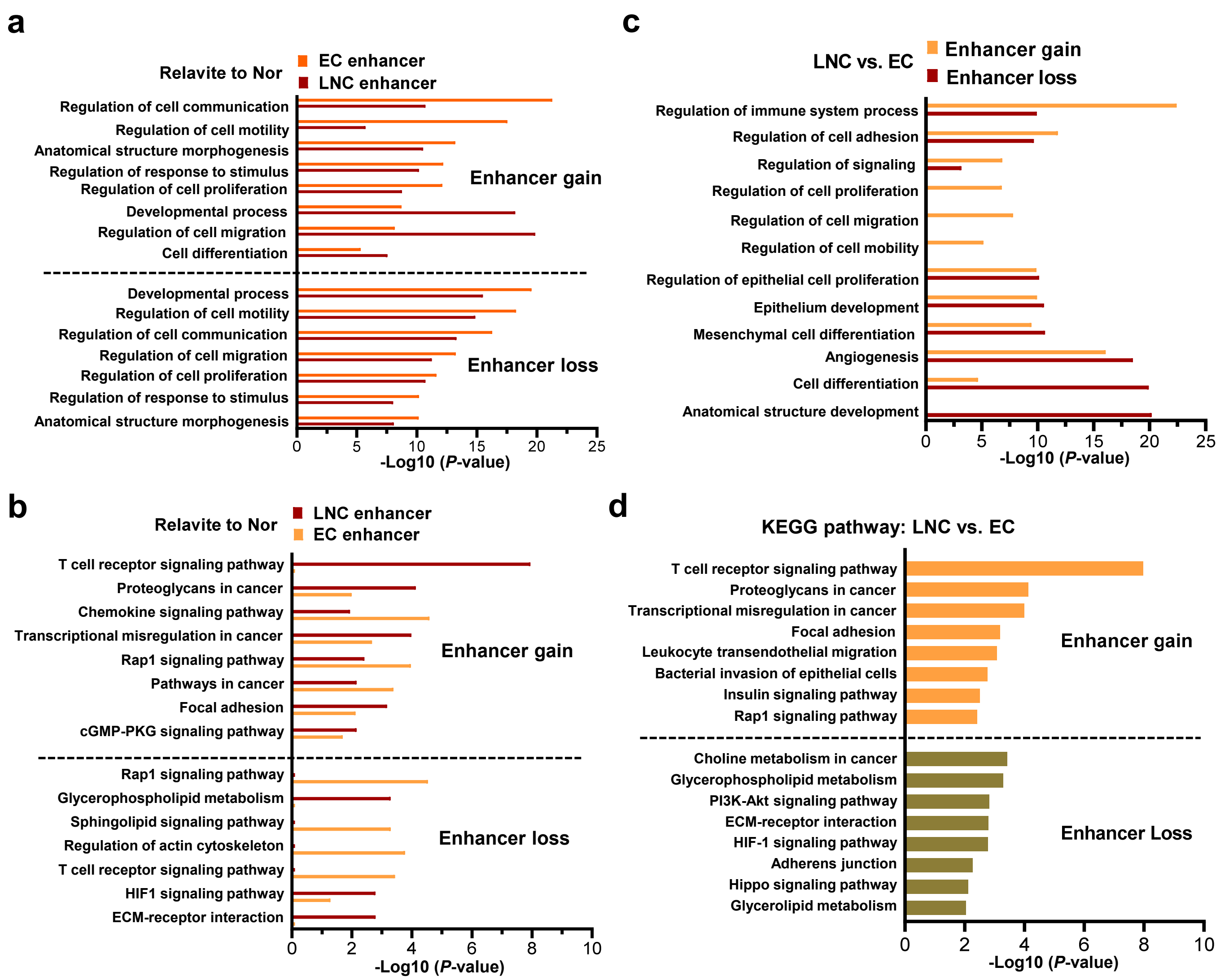
a
Relavite to Nor
EC enhancer
LNC enhancer
Regulation of cell communication
Regulation of cell motility
Anatomical structure morphogenesis
Regulation of response to stimulus
Regulation of cell proliferation
Developmental process
Regulation of cell migration
Cell differentiation
Enhancer gain
Developmental process
Regulation of cell motility
Regulation of cell communication
Regulation of cell migration
Regulation of cell proliferation
Regulation of response to stimulus
Anatomical structure morphogenesis
Enhancer loss
0 5 10 15 20 25
-Log10 (P-value)
c
LNC vs. EC
Enhancer gain
Enhancer loss
Regulation of immune system process
Regulation of cell adhesion
Regulation of signaling
Regulation of cell proliferation
Regulation of cell migration
Regulation of cell mobility
Regulation of epithelial cell proliferation
Epithelium development
Mesenchymal cell differentiation
Angiogenesis
Cell differentiation
Anatomical structure development
0 5 10 15 20 25
-Log10 (P-value)
b
Relavite to Nor
LNC enhancer
EC enhancer
T cell receptor signaling pathway
Proteoglycans in cancer
Chemokine signaling pathway
Transcriptional misregulation in cancer
Rap1 signaling pathway
Pathways in cancer
Focal adhesion
cGMP-PKG signaling pathway
Enhancer gain
Rap1 signaling pathway
Glycerophospholipid metabolism
Sphingolipid signaling pathway
Regulation of actin cytoskeleton
T cell receptor signaling pathway
HIF1 signaling pathway
ECM-receptor interaction
Enhancer loss
0 2 4 6 8 10
-Log10 (P-value)
d
KEGG pathway: LNC vs. EC
T cell receptor signaling pathway
Proteoglycans in cancer
Transcriptional misregulation in cancer
Focal adhesion
Leukocyte transendothelial migration
Bacterial invasion of epithelial cells
Insulin signaling pathway
Rap1 signaling pathway
Enhancer gain
Choline metabolism in cancer
Glycerophospholipid metabolism
PI3K-Akt signaling pathway
ECM-receptor interaction
HIF-1 signaling pathway
Adherens junction
Hippo signaling pathway
Glycerolipid metabolism
Enhancer Loss
0 2 4 6 8 10
-Log10 (P-value)

Supplementary Figure 10. Functional analysis for differential enhancer-associated genes.

a-b. GO (a) and KEGG pathway (b) analysis of the top 2,000 gained or lost enhancer-linked genes in EC and LNC relative to Nor.

c-d. GO (c) and KEGG pathway (d) analysis of the top 2,000 gained or lost enhancer-linked genes in LNC relative to EC.

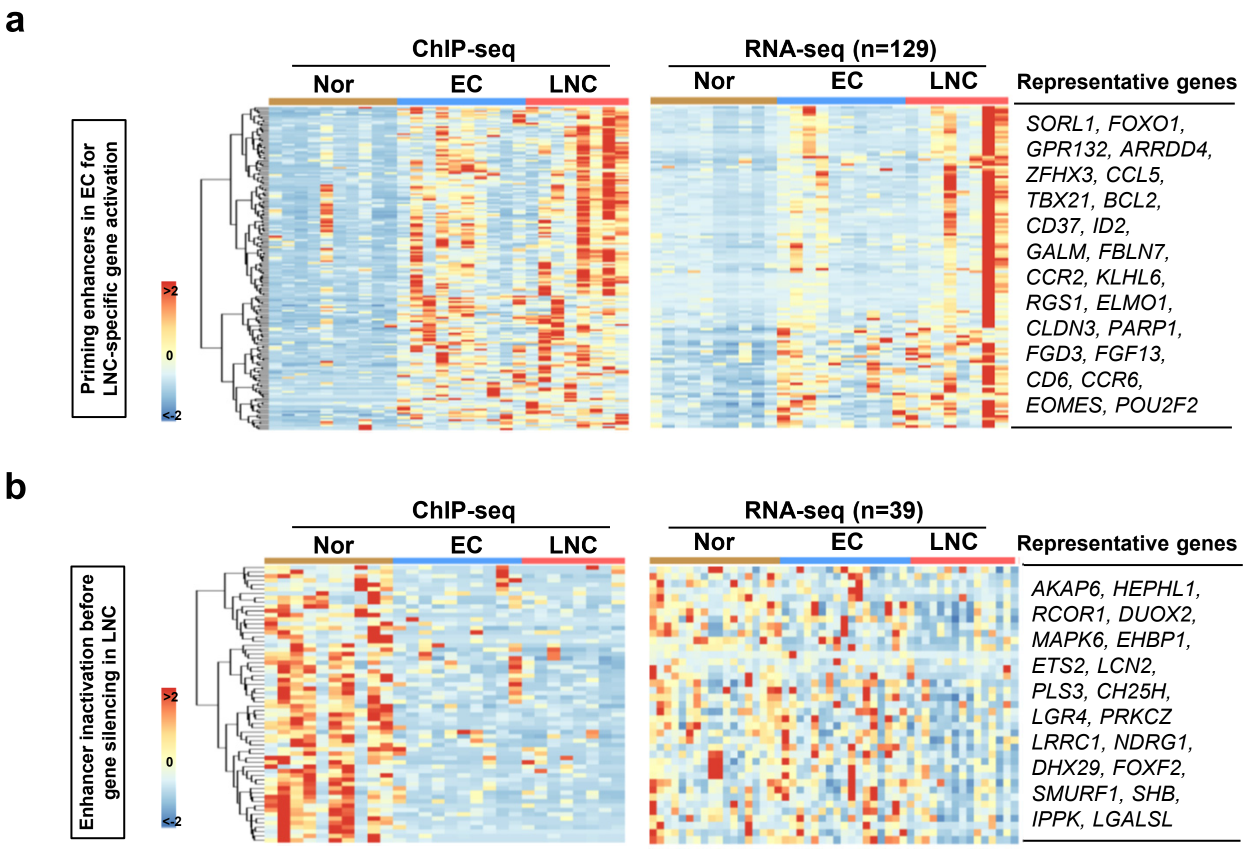
a
ChIP-seq
RNA-seq (n=129)
Nor
EC
LNC
Nor
EC
LNC
Representative genes
Priming enhancers in EC for LNC-specific gene activation
>2
0
<-2
SORL1, FOXO1,
GPR132, ARRDD4,
ZFHX3, CCL5,
TBX21, BCL2,
CD37, ID2,
GALM, FBLN7,
CCR2, KLHL6,
RGS1, ELMO1,
CLDN3, PARP1,
FGD3, FGF13,
CD6, CCR6,
EOMES, POU2F2
b
ChIP-seq
RNA-seq (n=39)
Nor
EC
LNC
Nor
EC
LNC
Representative genes
Enhancer inactivation before gene silencing in LNC
>2
0
<-2
AKAP6, HEPHL1,
RCOR1, DUOX2,
MAPK6, EHBP1,
ETS2, LCN2,
PLS3, CH25H,
LGR4, PRKCZ
LRRC1, NDRG1,
DHX29, FOXF2,
SMURF1, SHB,
IPPK, LGALSL

Supplementary Figure 11. Enhancer gain or loss prior to transcriptional alterations.

a. Heatmap showing a subgroup of gained H3K27ac-marked enhancers in EC and LNC. The associated gene expression was not significantly gained in EC but was significantly gained in LNC samples.

b. Heatmap showing a subgroup of lost H3K27ac-marked enhancers in EC and LNC. The associated gene expression was not significantly lost in EC but was significantly lost in LNC samples. Source data are provided as a Source Data file.

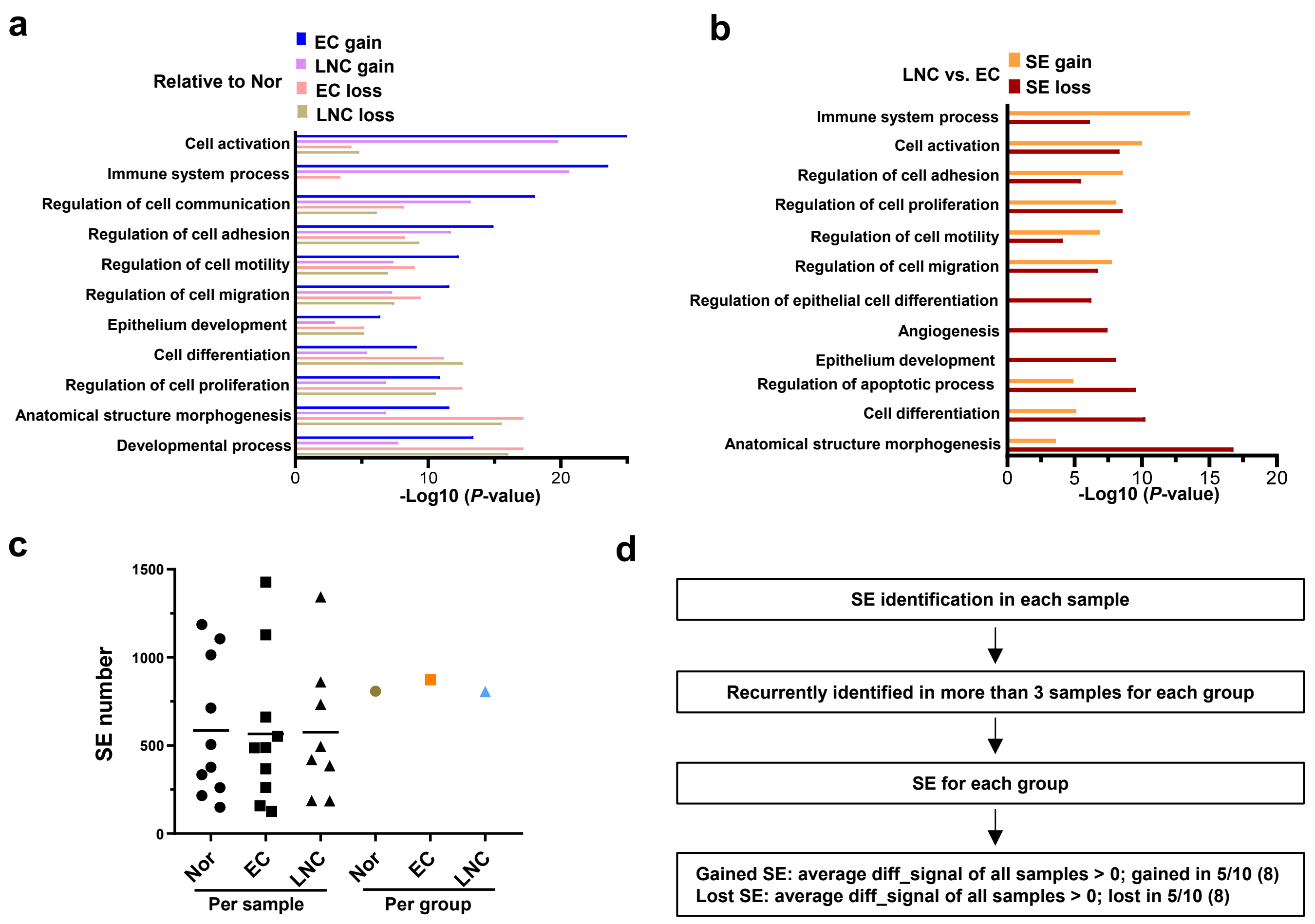

e

Differential SE identification by each sample

Regions	Definition	Total	Relative to Nor				Relative to EC	
			Recurrently gained in EC	Recurrently lost in EC	Recurrently gained in LNC	Recurrently lost in LNC	Recurrently gained in LNC	Recurrently lost in LNC
**SEs**	H3K27ac+/ promoter-	1,223	615	505	491	429	373	415
**Overlap with (Fig. S6g)**	H3K27ac+/ promoter-	948 (1,042)	387 (436)	355 (481)	427 (341)	310 (255)	271 (437)	179 (217)
**Combination**	H3K27ac+/ promoter-	1,317	657	536	531	472	406	449

Note: Fig. S6g-differential SE identification by mean signal for each group.

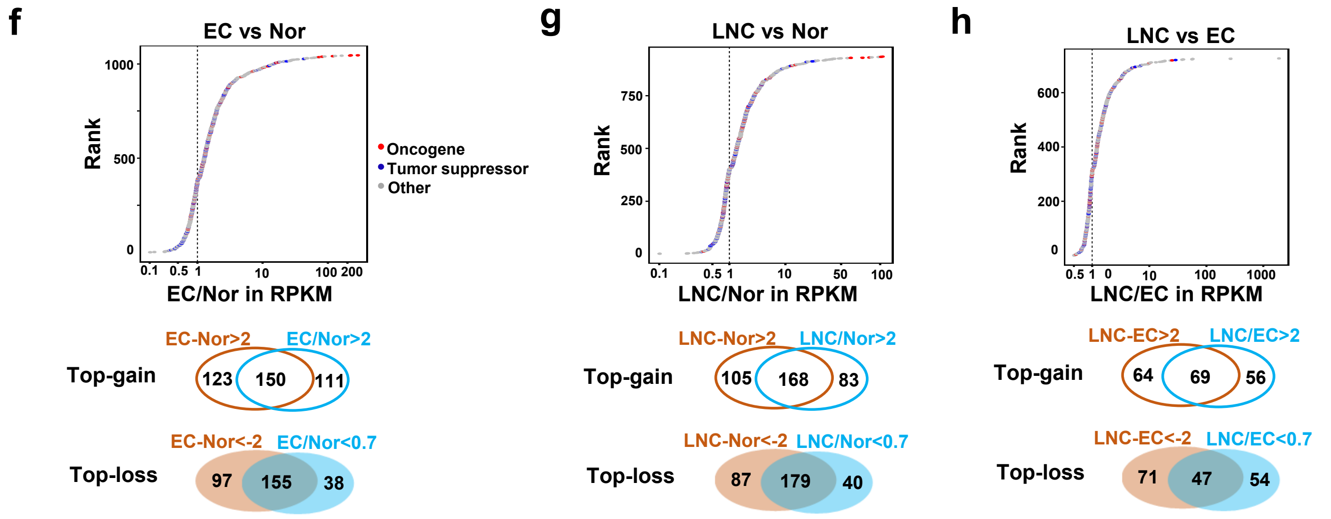

Supplementary Figure 12. Supporting data for super-enhancer analysis.

a. GO analysis of altered (gained or lost) super-enhancer-associated genes in EC and LNC relative to Nor.

b. GO analysis of altered (gained or lost) super-enhancer-associated genes in LNC relative to EC.

c. Comparison of the number of super-enhancers calculated from each H3K27ac profile or average H3K27ac signals per group. Lines indicate the mean values of the number of super-enhancers.

d. The pipeline for identifying altered super-enhancers based on SEs identified from each sample. Super-enhancers that were recurrently identified in ≥ 3 samples for each group were defined as super-enhancers for each group. The criteria for identifying altered enhancers were identical to that in Supplementary Figure 6g.

e. Number of the total, recurrently gained, lost super-enhancers in EC or LNC relative to Nor, as well as number of recurrently gained and lost super-enhancers in LNC relative to EC. Super-enhancers (SEs) were identified from combinations from each sample. The overlapped and combined number of presented super-enhancers were displayed. The number of super-enhancers shown in Supplementary Figure 6g was presented in red.

f-h. Identification of differential super-enhancers by fold change of super-enhancer-covered H3K27ac signals. All super-enhancers were ranked according to the fold change between two compared groups. In the lower panel, we combined fold change and absolute difference of super-enhancer-covered H3K27ac signals to identify top gained or lost super-enhancers (EC vs. Nor, LNC vs. Nor, and LNC vs.EC). For identifying top gained super-enhancers, the super-enhancers with absolute difference > 2 and fold change > 2 were overlapped; for identifying top lost super-enhancers, the super-enhancers with absolute difference < -2 and fold change < 0.7 (to retain a comparable number of lost super-enhancers when considering fold change) were overlapped. Source data are provided as a Source Data file.

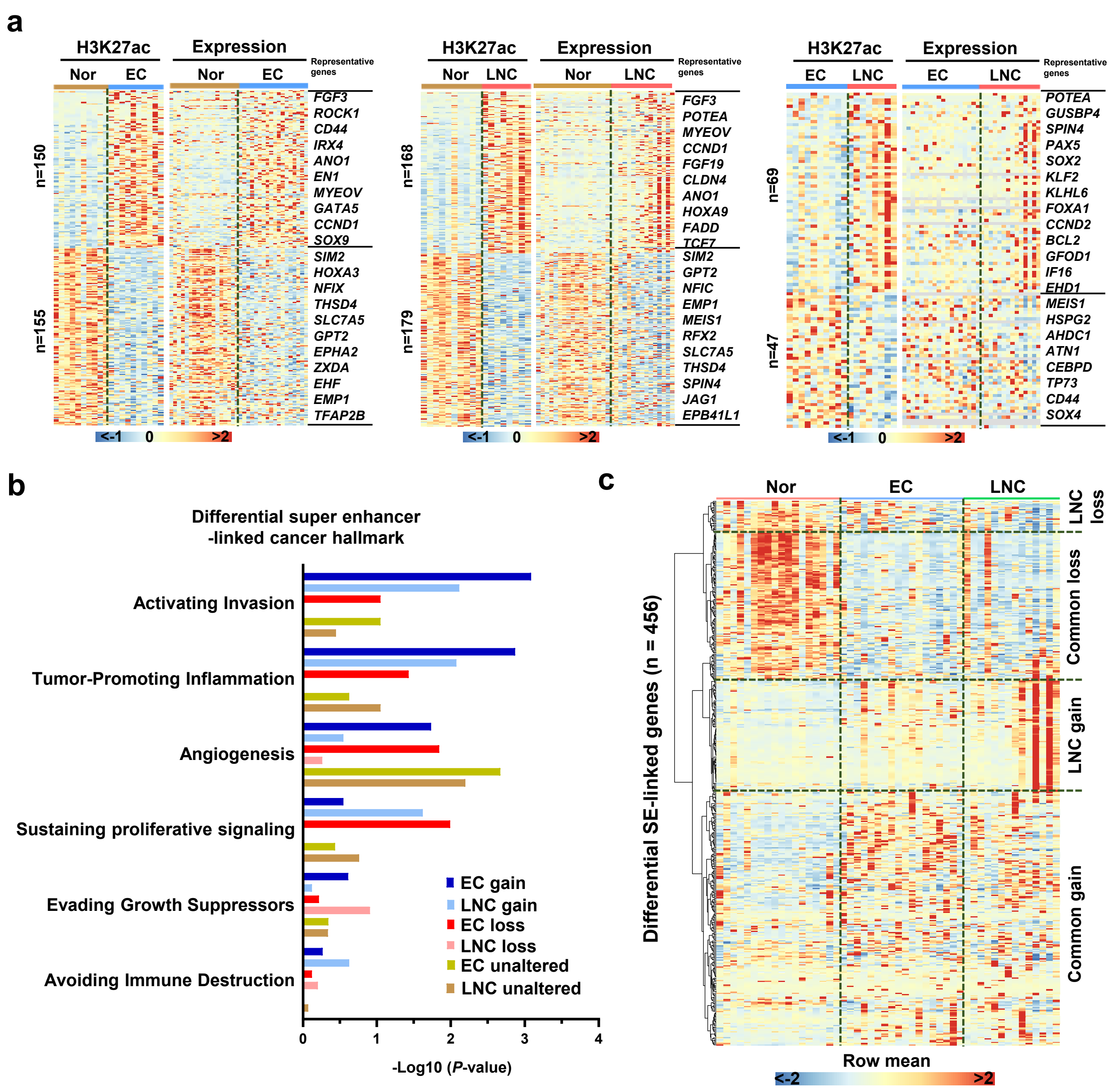

a
H3K27ac
Expression
Nor
EC
LNC
Representative genes
n=150
n=155
FGF3
ROCK1
CD44
IRX4
ANO1
EN1
MYEOV
GATA5
CCND1
SOX9
SIM2
HOXA3
NFIX
THSD4
SLC7A5
GPT2
EPHA2
ZXDA
EHF
EMP1
TFAP2B
n=168
n=179
FGF3
POTEA
MYEOV
CCND1
FGF19
CLDN4
ANO1
HOXA9
FADD
TCF7
SIM2
GPT2
NFIC
EMP1
MEIS1
RFX2
SLC7A5
THSD4
SPIN4
JAG1
EPB41L1
n=69
n=47
POTEA
GUSBP4
SPIN4
PAX5
SOX2
KLF2
KLHL6
FOXA1
CCND2
BCL2
GFOD1
IF16
EHD1
MEIS1
HSPG2
AHDC1
ATN1
CEBPD
TP73
CD44
SOX4
<-1
0
>2
b
Differential super enhancer -linked cancer hallmark
Activating Invasion
Tumor-Promoting Inflammation
Angiogenesis
Sustaining proliferative signaling
Evading Growth Suppressors
Avoiding Immune Destruction
EC gain
LNC gain
EC loss
LNC loss
EC unaltered
LNC unaltered
0
1
2
3
4
-Log10 (P-value)
c
Nor
EC
LNC
Differential SE-linked genes (n = 456)
LNC loss
Common loss
LNC gain
Common gain
Row mean
<-2
>2

Supplementary Figure 13. Supporting data for super-enhancer analysis.

a. Heatmap showing the H3K27ac signals within differential super-enhancers and expression of differential super-enhancer-associated genes. The differential super-enhancers were obtained from overlapped terms in Supplementary Figure 12f-g. Representative genes for each group were listed in the right panel.

b. Cancer hallmark analysis using differential SEs (EC gain, EC loss, LNC gain, and LNC loss relative to Nor)-associated genes. Nearby genes associated with unaltered super-enhancers in EC and LNC relative to Nor served as a control.

c. Heatmap showing the expression of 456 differential super-enhancer-associated genes across Nor, EC, and LNC samples. These genes were a combination of differential super-enhancer-associated genes in a. These genes were classified into 4 sub-groups: LNC-specific loss, common loss, LNC-specific gain, and common gain. Source data are provided as a Source Data file.

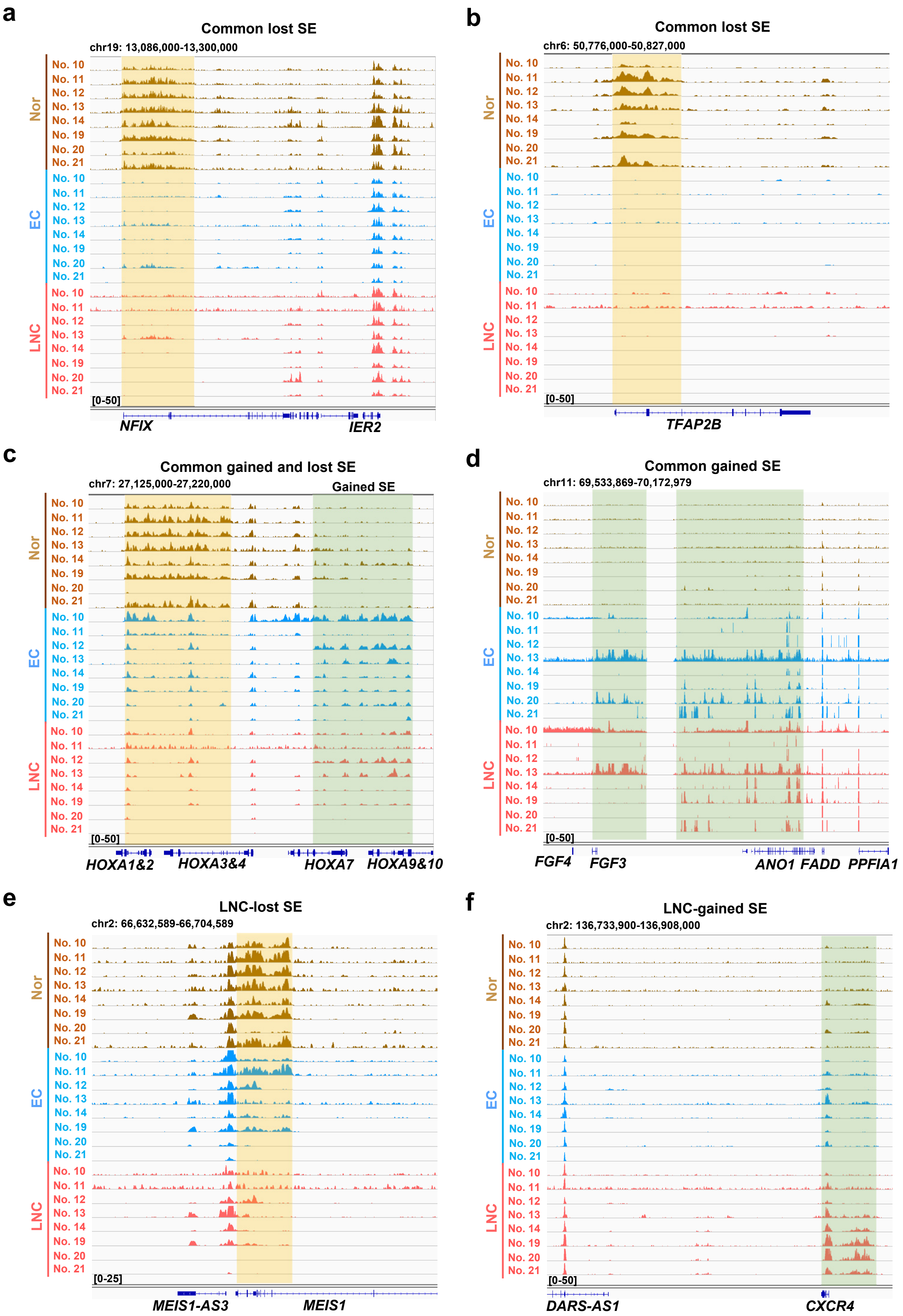
a
Common lost SE
chr19: 13,086,000-13,300,000
Nor
EC
LNC
No. 10
No. 11
No. 12
No. 13
No. 14
No. 19
No. 20
No. 21
[0-50]
NFIX
IER2
b
Common lost SE
chr6: 50,776,000-50,827,000
[0-50]
TFAP2B
c
Common gained and lost SE
chr7: 27,125,000-27,220,000
Gained SE
[0-50]
HOXA1&2
HOXA3&4
HOXA7
HOXA9&10
d
Common gained SE
chr11: 69,533,869-70,172,979
[0-50]
FGF4
FGF3
ANO1
FADD
PPFIA1
e
LNC-lost SE
chr2: 66,632,589-66,704,589
[0-25]
MEIS1-AS3
MEIS1
f
LNC-gained SE
chr2: 136,733,900-136,908,000
[0-50]
DARS-AS1
CXCR4

Supplementary Figure 14. Examples for gained and lost super-enhancers.

a-f. H3K27ac tracks showing commonly lost (highlighted in yellow) and gained (highlighted in green) super-enhancers (SEs) relative to Nor. It was worth noticing that there were two neighboring altered super-enhancers, including a lost SE covering *HOXA1/2/3/4* as well as a gained SE covering *HOXA7/9/10*.

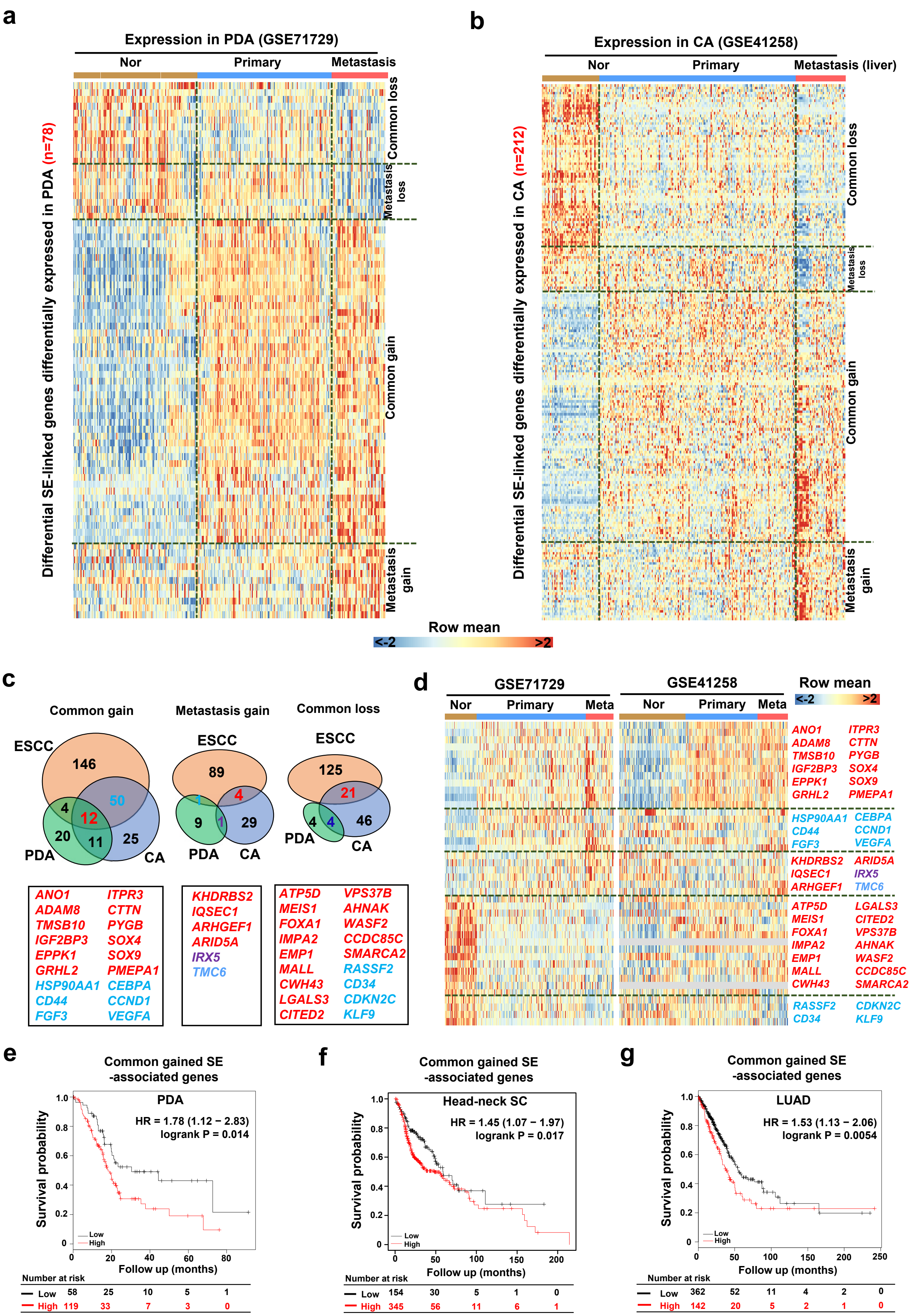
a
Expression in PDA (GSE71729)
Nor
Primary
Metastasis
Differential SE-linked genes differentially expressed in PDA (n=78)
Common loss
Metastasis loss
Common gain
Metastasis gain
b
Expression in CA (GSE41258)
Nor
Primary
Metastasis (liver)
Differential SE-linked genes differentially expressed in CA (n=212)
Common loss
Metastasis loss
Common gain
Metastasis gain
Row mean
<-2
>2
c
Common gain
Metastasis gain
Common loss
ESCC
146
50
4
12
20
11
25
PDA
CA
89
1
4
9
1
29
125
21
4
4
46
ANO1 ITPR3
ADAM8 CTTN
TMSB10 PYGB
IGF2BP3 SOX4
EPPK1 SOX9
GRHL2 PMEPA1
HSP90AA1 CEBPA
CD44 CCND1
FGF3 VEGFA
KHDRBS2
IQSEC1
ARHGEF1
ARID5A
IRX5
TMC6
ATP5D VPS37B
MEIS1 AHNAK
FOXA1 WASF2
IMPA2 CCDC85C
EMP1 SMARCA2
MALL RASSF2
CWH43 CD34
LGALS3 CDKN2C
CITED2 KLF9
d
GSE71729
GSE41258
Nor
Primary
Meta
Row mean
<-2
>2
ANO1 ITPR3
ADAM8 CTTN
TMSB10 PYGB
IGF2BP3 SOX4
EPPK1 SOX9
GRHL2 PMEPA1
HSP90AA1 CEBPA
CD44 CCND1
FGF3 VEGFA
KHDRBS2 ARID5A
IQSEC1 IRX5
ARHGEF1 TMC6
ATP5D LGALS3
MEIS1 CITED2
FOXA1 VPS37B
IMPA2 AHNAK
EMP1 WASF2
MALL CCDC85C
CWH43 SMARCA2
RASSF2 CDKN2C
CD34 KLF9
e
Common gained SE
-associated genes
PDA
HR = 1.78 (1.12 − 2.83)
logrank P = 0.014
Survival probability
Follow up (months)
Low
High
Number at risk
Low 58 25 10 5 1
High 119 33 7 3 0
f
Common gained SE
-associated genes
Head-neck SC
HR = 1.45 (1.07 − 1.97)
logrank P = 0.017
Survival probability
Follow up (months)
Low
High
Number at risk
Low 154 30 5 1 0
High 345 56 11 6 1
g
Common gained SE
-associated genes
LUAD
HR = 1.53 (1.13 − 2.06)
logrank P = 0.0054
Survival probability
Follow up (months)
Low
High
Number at risk
Low 362 52 11 4 2 0
High 142 20 5 2 1 0

Supplementary Figure 15. Differential super-enhancer-linked gene expression in other cancers.

a-b. DEG analysis of differential super-enhancers-linked genes in PDA (GSE71729) and CA (GSE41258). These genes were categorized into four indicated subgroups.

c. Common gained, metastasis gained, and common lost genes from ESCC, PDA, and CA were overlapped and presented in Venn diagram. Representative genes for overlapped genes were shown below, and the color of gene names was matched to the overlapped groups.

d. Heatmap showing the expression of representative genes in c.

e-g. Survival analysis comparing patient groups with high or low expression of top gained SEs-associated genes in ESCC using Kaplan-Meier plotter with PDA (e), Head-neck squamous cancer (SC) (f), and LUAD (g) patients from TCGA database. LogRank test was performed for the survival data. The number of patients was listed below. Source data are provided as a Source Data file.

a

Common lost enhancer

DNA motif	TF	*P*-value	% targets
TGGGCTACCTAA	OSR1	1e-16	0.33
AGCTTTCACA	TBX21	1e-15	35.1
TATAATTAAAAG	ARID3A	1e-13	0.48
GTTTGGCACA	NFIA	1e-13	11.93
AACGCAGGGC	ZNF416	1e-13	11.12
GAGGCCACTC	NKX2.5	1e-12	12.93
ATAGCCTGAT	TCF4	1e-12	0.29
CTGACATTAG	MEIS1	1e-9	4.16
GTAGAAAAGGAA	ELF3	1e-7	0.44

b

LNC-specific lost enhancer

DNA motif	TF	*P*-value	% targets
GGAGAGTCAGCC	MAFA	1e-10	2.55
GCTCATGGTGTA	SOX14	1e-9	1.28
AAATTCTGCCCA	BCL11A	1e-9	1.28
CTGTCCAGTTTC	ZNF416	1e-9	3.28
TGTTGTAAAT	LIN45	1e-8	3.1
GTTGGCTGCG	NFIX	1e-8	2.19
CGCGTCTCCG	RFXDC2	1e-7	2.01
GTTTTGTGATTT	FOXA2	1e-7	0.73

c

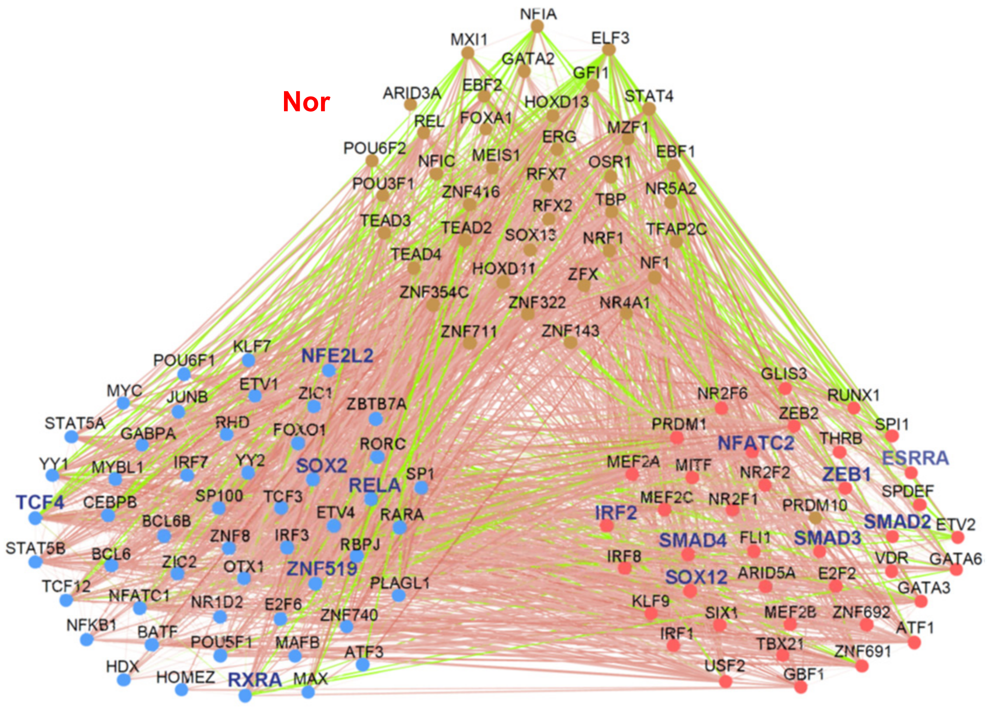

Supplementary Figure 16. Altered enhancer predicted TF binding activities and network.

a-b. Top transcription factor (TF) binding motifs predicted from common lost (a) or LNC-specific lost (b) enhancers using HOMER. The predicted TFs were ranked by *P* value.

c. TF interaction network across three groups of samples using top predicted TFs. Correlation between two TFs was calculated according to the RNA-seq expression in three cohorts, and the correlation within the same group was not presented. A positive correlation between two TFs was linked with red lines, and negative correlation between two TFs was linked with green lines.

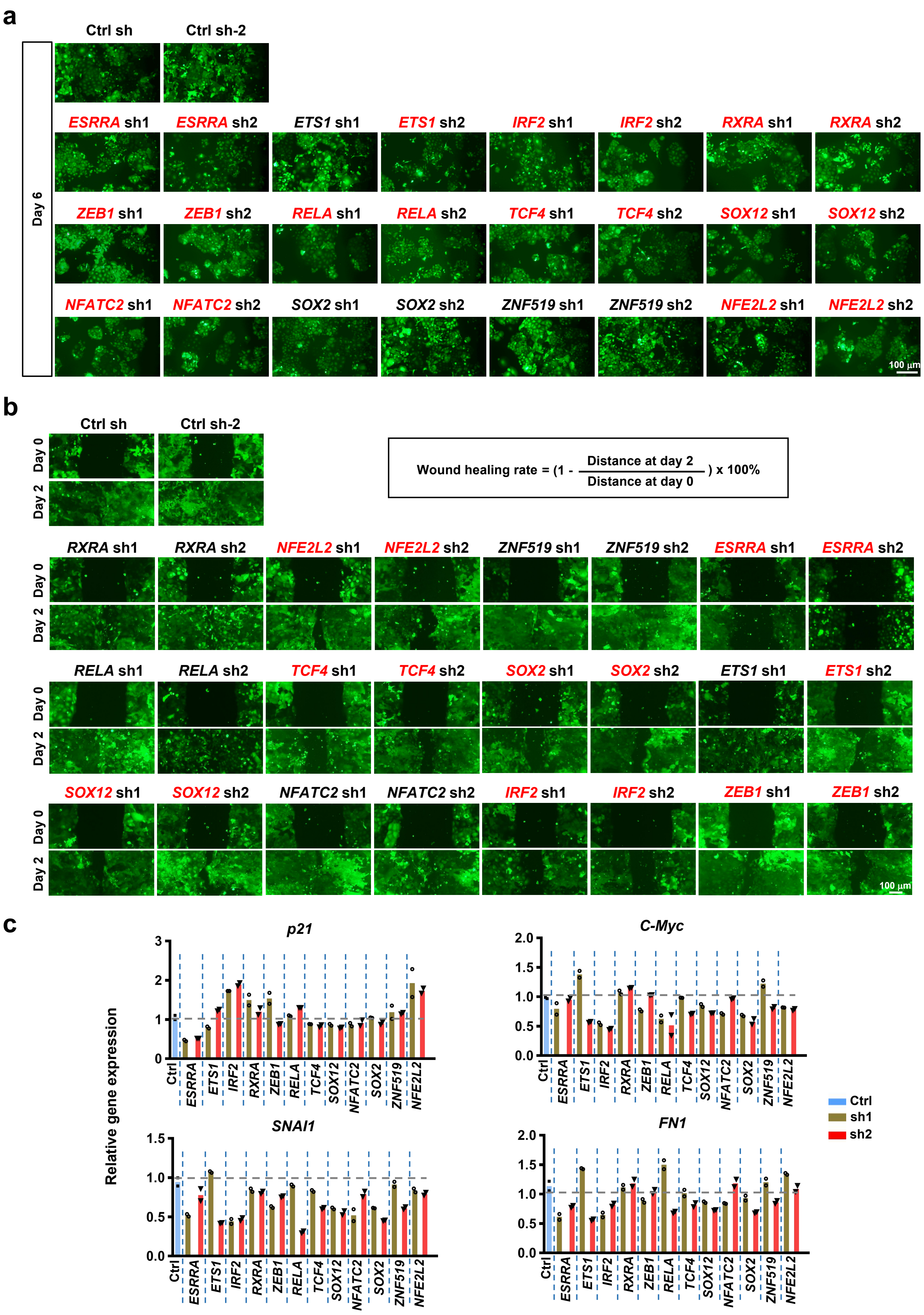

a
Ctrl sh
Ctrl sh-2
Day 6
ESRRA sh1
ESRRA sh2
ETS1 sh1
ETS1 sh2
IRF2 sh1
IRF2 sh2
RXRA sh1
RXRA sh2
ZEB1 sh1
ZEB1 sh2
RELA sh1
RELA sh2
TCF4 sh1
TCF4 sh2
SOX12 sh1
SOX12 sh2
NFATC2 sh1
NFATC2 sh2
SOX2 sh1
SOX2 sh2
ZNF519 sh1
ZNF519 sh2
NFE2L2 sh1
NFE2L2 sh2
100 μm
b
Day 0
Day 2
Wound healing rate = (1 - Distance at day 2 / Distance at day 0) x 100%
c
p21
C-Myc
SNAI1
FN1
Relative gene expression
Ctrl
sh1
sh2

Supplementary Figure 17. Supporting data for functional analysis of gained enhancer-predicted TFs.

a. Representative images showing the GFP fluorescence as indications for shRNAs expression. Lentiviral infected TE1 cells were enriched to obtain 100% GFP positive cells. GFP fluorescence was used for cell counting. Three independent experiments with three repeats were performed, and similar results were obtained.

b. Representative images showing the wound healing status at day 0 and day 2 using GFP fluorescence. The formula for calculating the wound healing rate was provided. Three independent experiments with three repeats were performed, and similar results were obtained.

c. Quantitative real-time PCR analysis of *p21*, *c-Myc*, *SNAI1*, and *FN1* expression upon knockdown of 12 gained enhancer-predicted TFs. n=2 biologically independent experiments examined. Source data are provided as a Source Data file.

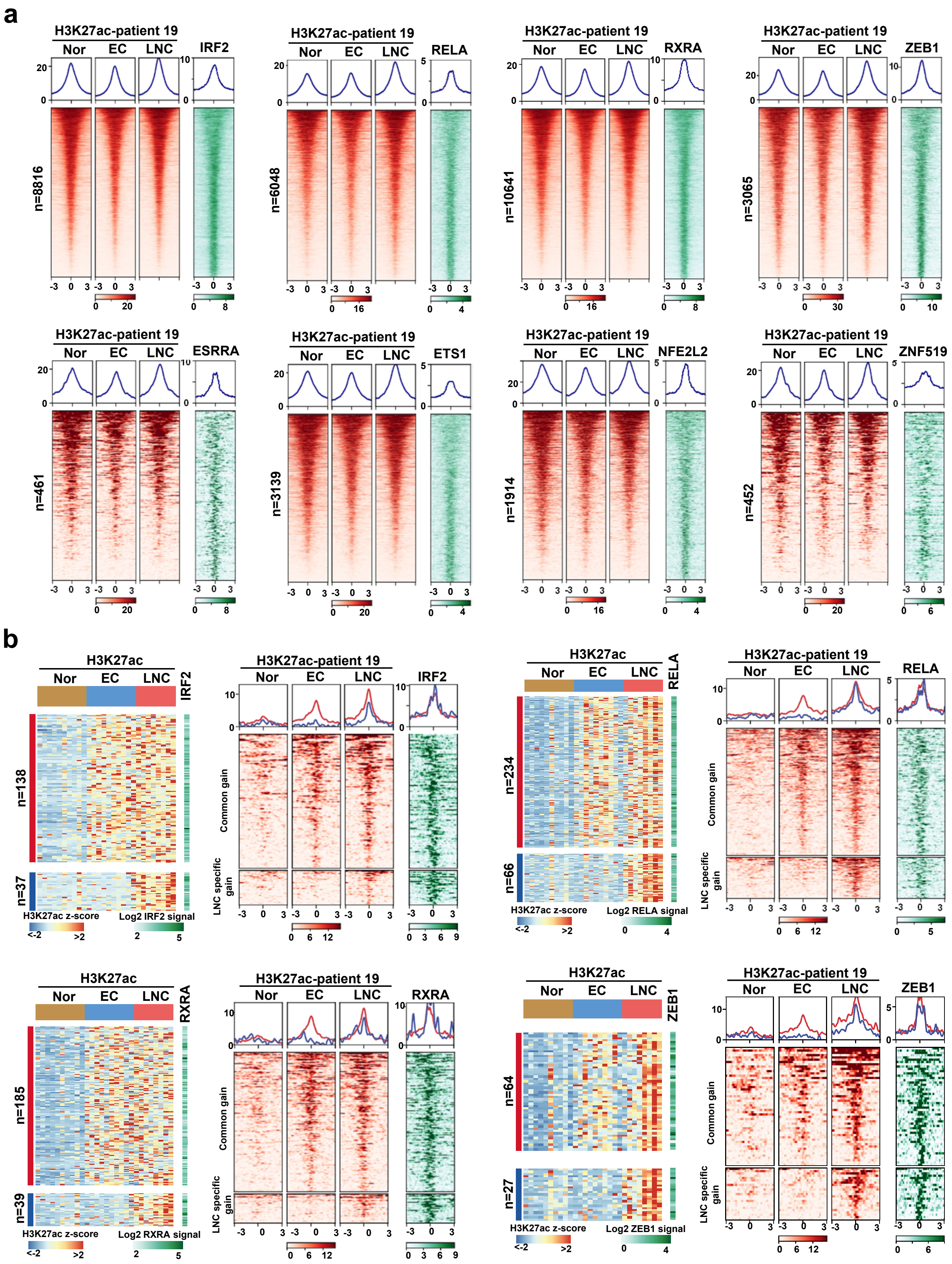

a
H3K27ac-patient 19
Nor
EC
LNC
IRF2
RELA
RXRA
ZEB1
ESRRA
ETS1
NFE2L2
ZNF519
n=8816
n=6048
n=10641
n=3065
n=461
n=3139
n=1914
n=452
b
H3K27ac
n=138
n=37
n=234
n=66
n=185
n=39
n=64
n=27
Common gain
LNC specific gain
H3K27ac z-score
<-2
>2
Log2 IRF2 signal
Log2 RELA signal
Log2 RXRA signal
Log2 ZEB1 signal

Supplementary Figure 18. Co-occupancy between key TF binding peaks and H3K27ac peaks.

a. All H3K27ac enhancers were overlapped with 8 TF binding profiles (dataset available in ENCODE database), and co-occupied regions were presented in a representative patient (patient 19) or in cell lines (Information was provided in Supplementary Data 20).

b. Common gained and LNC-specific gained H3K27ac enhancers were overlapped with 8 TF binding profiles respectively, and co-occupied regions were represented, and associated genes were listed in Supplementary Data 20. Source data are provided as a Source Data file.

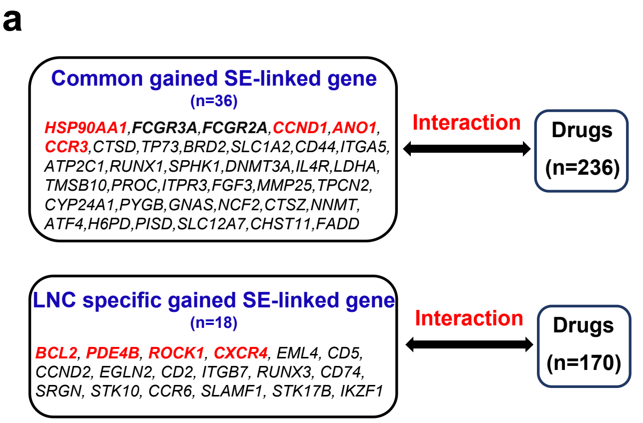

b

Target	Chemical	Type	MCE Cat. No.
HSP90AA1	Alvespimycin hydrochloride (AH)	Inhibitor	HY-12024
CCND1	Palbociclib	Inhibitor	HY-50767
ANO1	Endovion	Inhibitor	HY-105917
CCR3	SB297006	Antagonist	HY-103361
BCL2	Navitoclax	Inhibitor	HY-10087
PDE4B	ML-030	Inhibitor	HY-103050
ROCK1	Hydroxyfasudil	Inhibitor	HY-13911
CXCR4	IT1t dihydrochloride (IT1t)	Antagonist	HY-101458A

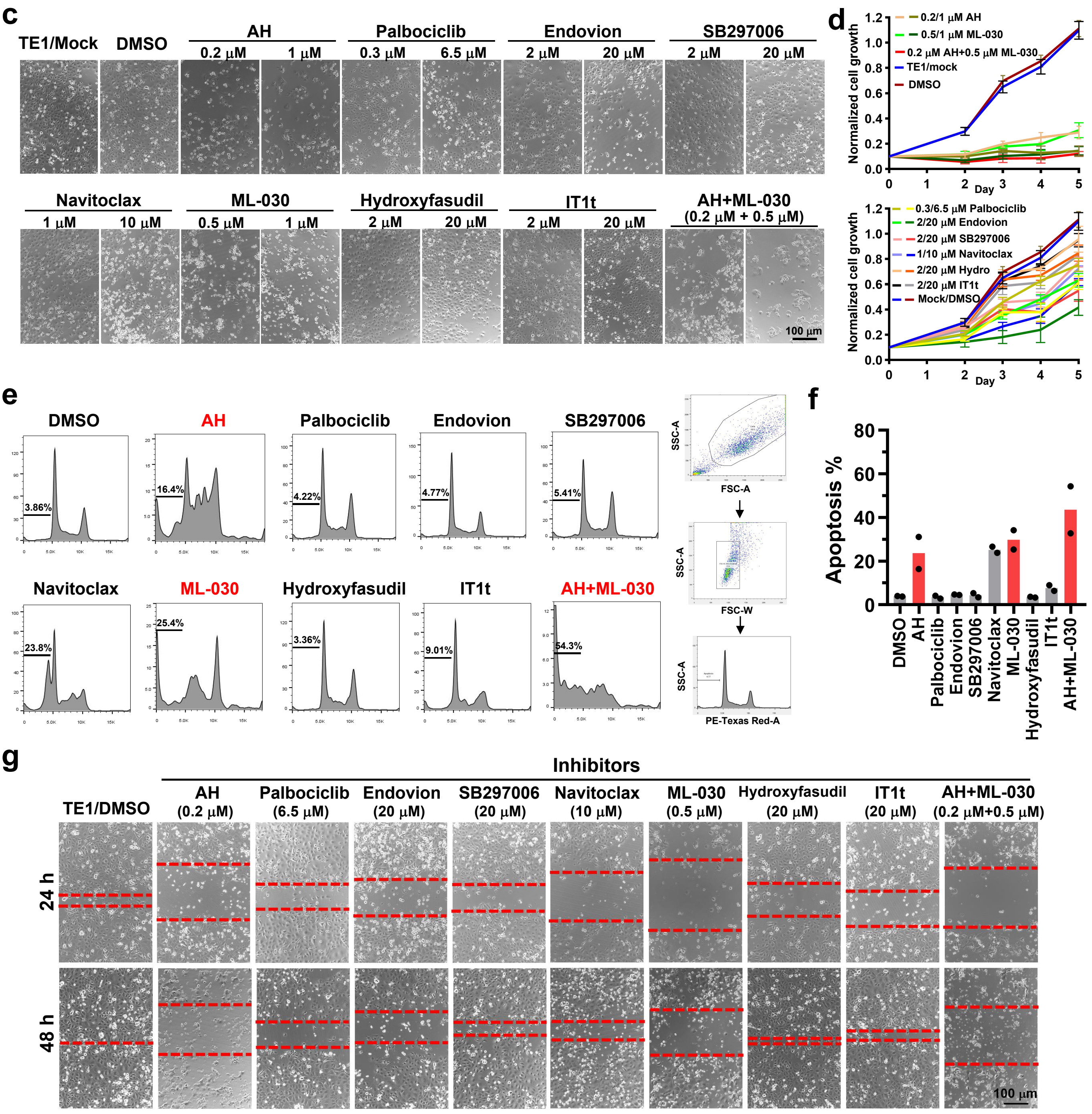

Supplementary Figure 19. Supporting data for functional analysis of super-enhancer predicted drugs.

a. Common gained and LNC specific gained super-enhancer (SE)-linked genes were subjected to the Drug Gene Interaction Database (DGIdb). 36 common gained SE-linked genes were potentially interacting with 236 drugs, and 18 LNC-specific gained SE-linked genes were potentially interacting with 170 drugs.

b. Top 8 targets predicted from gained super-enhancer (SE)-linked gene-drug interactions, their chemical inhibitors, and catalog numbers from MCE (MCE Cat. No.) were listed.

c. Representative images showing TE1 cells responding to indicated concentrations of chemical inhibitors. AH+ML-030: combined use of the two chemicals. The DMSO and mock group served as controls. Two independent experiments with three repeats were performed, and similar results were obtained.

d. TE1 cells treated with chemical inhibitors were counted at day 0, 1, 2, 3, 4, and 5. Mock TE1 cells and DMSO group served as controls. Data are presented as mean values ± s.d.. n=3 biologically independent experiments examined.

e. Apoptotic analysis of TE1 cells responding to indicated chemical inhibitors with higher concentrations used in c. Representative results for apoptotic analysis were presented with the percentages of apoptotic cells, and an example for gating strategy was also presented for Endovion-treated group.

f. Statistical analyses of cell apoptosis in e. n=2 biologically independent experiments examined. Source data are provided as a Source Data file.

g. Representative images showing TE1 cell migration in responding to indicated concentrations of chemical inhibitors after scraping for 24 and 48 h. Dashed red lines indicates the edge of wounds. Three independent experiments with three repeats were performed, and similar results were obtained. Source data are provided as a Source Data file.

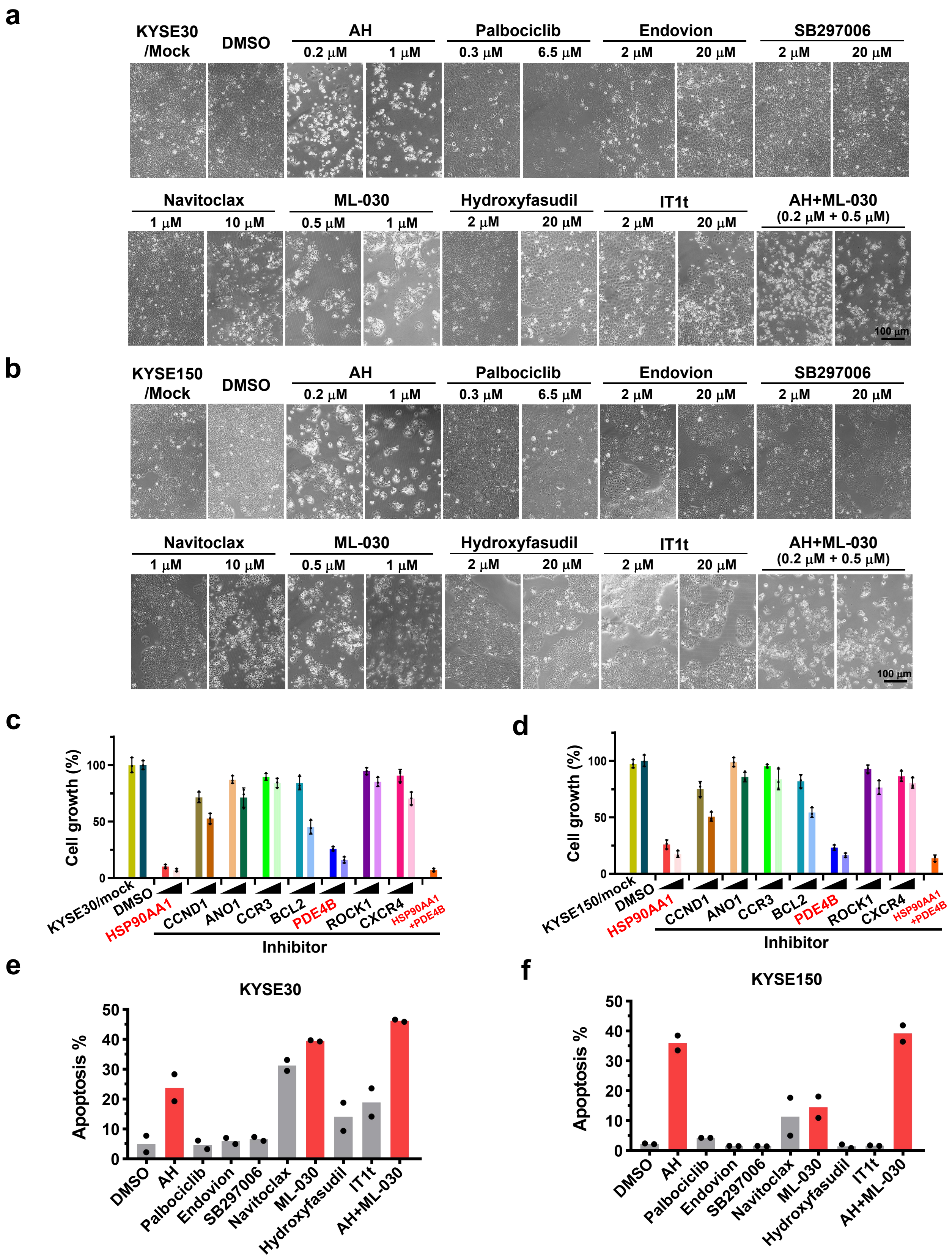
a
KYSE30 /Mock
DMSO
AH
0.2 μM
1 μM
Palbociclib
0.3 μM
6.5 μM
Endovion
2 μM
20 μM
SB297006
2 μM
20 μM
Navitoclax
1 μM
10 μM
ML-030
0.5 μM
1 μM
Hydroxyfasudil
2 μM
20 μM
IT1t
2 μM
20 μM
AH+ML-030
(0.2 μM + 0.5 μM)
100 μm
b
KYSE150 /Mock
DMSO
AH
0.2 μM
1 μM
Palbociclib
0.3 μM
6.5 μM
Endovion
2 μM
20 μM
SB297006
2 μM
20 μM
Navitoclax
1 μM
10 μM
ML-030
0.5 μM
1 μM
Hydroxyfasudil
2 μM
20 μM
IT1t
2 μM
20 μM
AH+ML-030
(0.2 μM + 0.5 μM)
100 μm
c
Cell growth (%)
100
50
0
KYSE30/mock
DMSO
HSP90AA1
CCND1
ANO1
CCR3
BCL2
PDE4B
ROCK1
CXCR4
HSP90AA1 +PDE4B
Inhibitor
d
Cell growth (%)
100
50
0
KYSE150/mock
DMSO
HSP90AA1
CCND1
ANO1
CCR3
BCL2
PDE4B
ROCK1
CXCR4
HSP90AA1 +PDE4B
Inhibitor
e
KYSE30
Apoptosis %
50
40
30
20
10
0
DMSO
AH
Palbociclib
Endovion
SB297006
Navitoclax
ML-030
Hydroxyfasudil
IT1t
AH+ML-030
f
KYSE150
Apoptosis %
50
40
30
20
10
0
DMSO
AH
Palbociclib
Endovion
SB297006
Navitoclax
ML-030
Hydroxyfasudil
IT1t
AH+ML-030

Supplementary Figure 20. Additional data for functional analysis of super-enhancer predicted drugs.

a-b. Representative images showing KYSE30 (a) and KYSE150 (b) cells responding to indicated concentrations of chemical inhibitors. AH+ML-030: combined use of the two chemicals. The DMSO and mock group served as controls. Two independent experiments with three repeats were performed, and similar results were obtained.

c-d. KYSE30 (c) and KYSE150 (d) cells were treated with DMSO, with each of 8 chemical inhibitors (higher concentration used in a) separately, or with a combination of HSP90AA1 and PDE4B inhibitors (AH+ML-030). Cell number was counted at 72 h using cell counter. Data are presented as mean values ± s.d.. n=3 biologically independent experiments presented.

e-f. Apoptotic analysis of KYSE30 (e) and KYSE150 (f) cells responding to indicated chemical inhibitors with higher concentrations used in a. Representative results for apoptotic analysis were presented with the percentages of apoptotic cells. n=2 biologically independent experiments presented. Source data are provided as a Source Data file.

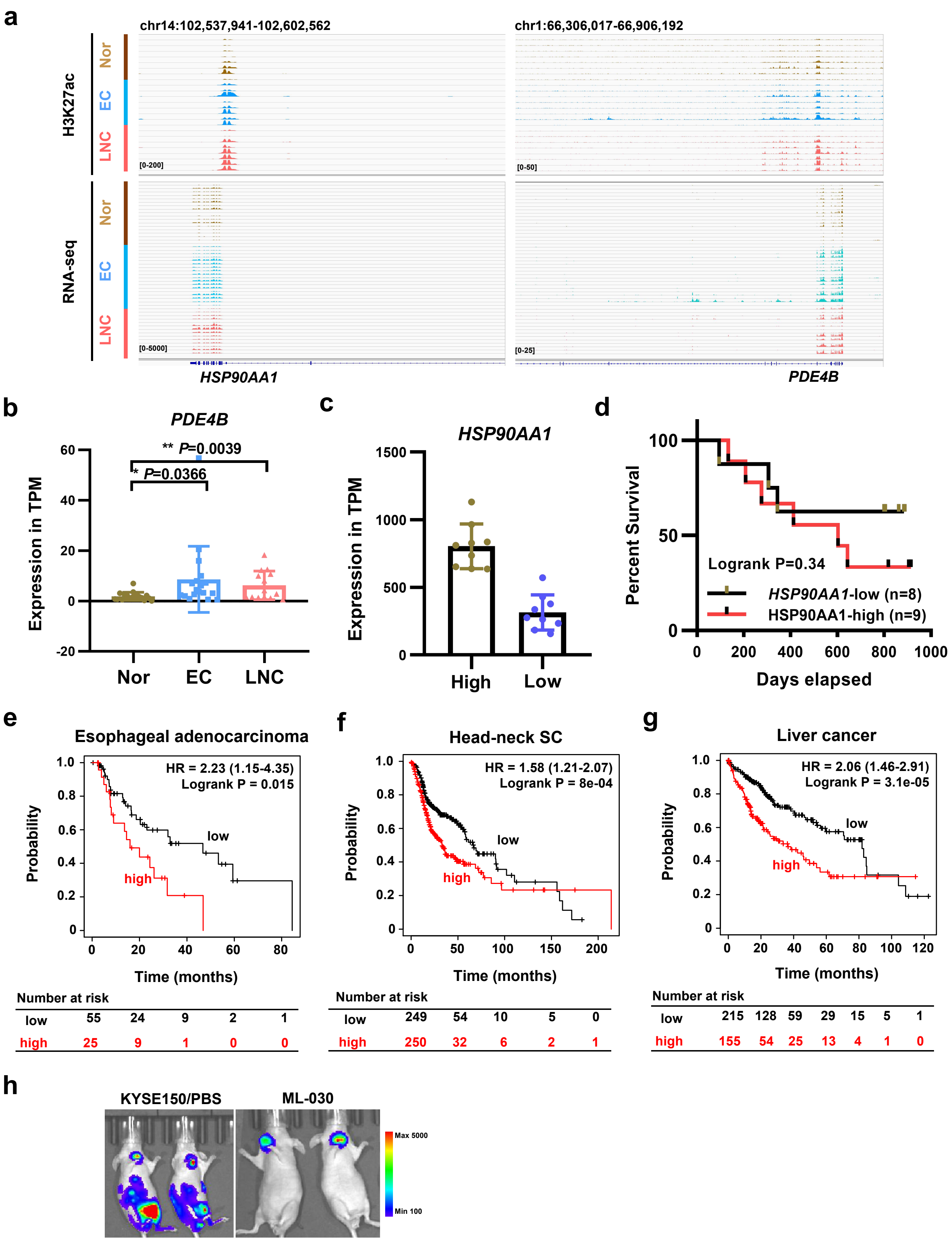
a
chr14:102,537,941-102,602,562
chr1:66,306,017-66,906,192
H3K27ac
RNA-seq
Nor
EC
LNC
[0-200]
[0-50]
[0-5000]
[0-25]
HSP90AA1
PDE4B
b
PDE4B
** P=0.0039
* P=0.0366
Expression in TPM
60
40
20
0
-20
Nor
EC
LNC
c
HSP90AA1
Expression in TPM
1500
1000
500
0
High
Low
d
Percent Survival
100
50
0
Logrank P=0.34
HSP90AA1-low (n=8)
HSP90AA1-high (n=9)
0
200
400
600
800
1000
Days elapsed
e
Esophageal adenocarcinoma
HR = 2.23 (1.15-4.35)
Logrank P = 0.015
low
high
Probability
Time (months)
Number at risk
low 55 24 9 2 1
high 25 9 1 0 0
f
Head-neck SC
HR = 1.58 (1.21-2.07)
Logrank P = 8e-04
low
high
Probability
Time (months)
Number at risk
low 249 54 10 5 0
high 250 32 6 2 1
g
Liver cancer
HR = 2.06 (1.46-2.91)
Logrank P = 3.1e-05
low
high
Probability
Time (months)
Number at risk
low 215 128 59 29 15 5 1
high 155 54 25 13 4 1 0
h
KYSE150/PBS
ML-030
Max 5000
Min 100

Supplementary Figure 21. Validation of HSP90AA1 and PDE4B functions.

a. H3K27ac and RNA-seq tracks showing gained super-enhancers and upregulated gene expression for *HSP90AA1* and *PDE4B*.

b. *PDE4B* expression in TPM in Nor, EC, and LNC samples. n=18 for Nor and EC; n=14 for LNC. Data are presented as mean values ± s.d. $*P < 0.05$, $**P < 0.01$, unpaired *t*-test (two-tailed). Source data are provided as a Source Data file.

c. Patients were divided into two sub-groups with high (n=9) or low (n=9) *HSP90AA1* expression in EC. The expression of *HSP90AA1* in two sub-groups was presented. Data are presented as mean values ± s.d.

d. Survival analysis of the patients with high (n=9) or low (n=8) *HSP90AA1* expression in EC (the survival information for one patient was not available). LogRank *P*-value was calculated using LogRank test.

e-g. Survival analysis comparing patient groups with high or low expression of *HSP90AA1* using Kaplan-Meier plotter with esophageal adenocarcinoma (e), Head-neck SC (f), and liver cancer (g) patients from TCGA database. The number of patients was listed below. LogRank test was performed for the survival data.

h. Bioluminescent imaging at 4 weeks post intramuscular injection (around the neck region to mimic esophagus cancer) of 1×10^5 KYSE150 cells stably expressing firefly luciferase cDNA that was introduced by lentiviruses. ML-030 (20 mg/kg) was administrated through intraperitoneal injection every three days, starting from the day after cell implantation. PBS group served as a control. Four mice were used for each group and two mice were presented in a representative image for image capture. Source data are provided as a Source Data file.

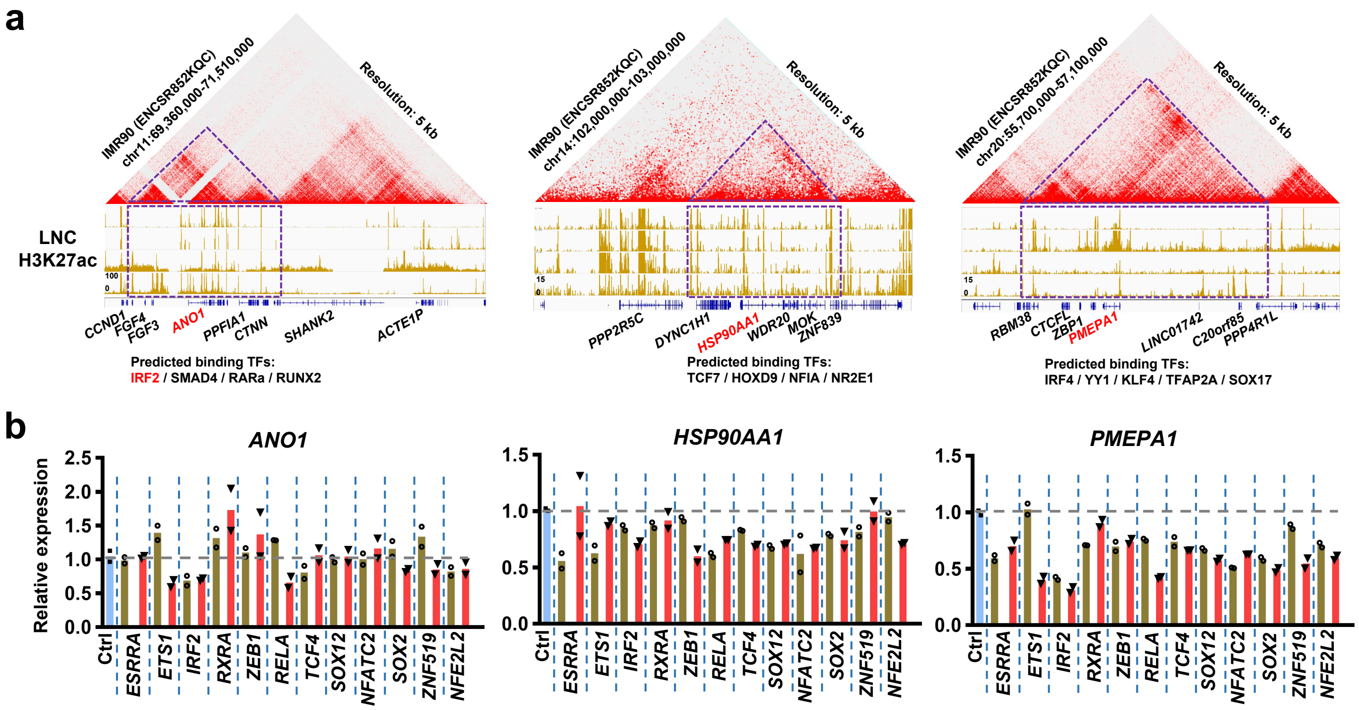
a
IMR90 (ENCSR852KQC)
chr11:69,360,000-71,510,000
Resolution: 5 kb
LNC
H3K27ac
CCND1
FGF4
FGF3
ANO1
PPFIA1
CTNN
SHANK2
ACTE1P
Predicted binding TFs:
IRF2 / SMAD4 / RARa / RUNX2
IMR90 (ENCSR852KQC)
chr14:102,000,000-103,000,000
Resolution: 5 kb
PPP2R5C
DYNC1H1
HSP90AA1
WDR20
MOK
ZNF839
Predicted binding TFs:
TCF7 / HOXD9 / NFIA / NR2E1
IMR90 (ENCSR852KQC)
chr20:55,700,000-57,100,000
Resolution: 5 kb
RBM38
CTCFL
ZBP1
PMEPA1
LINC01742
C20orf85
PPP4R1L
Predicted binding TFs:
IRF4 / YY1 / KLF4 / TFAP2A / SOX17
b
ANO1
HSP90AA1
PMEPA1
Relative expression
Ctrl
ESRRA
ETS1
IRF2
RXRA
ZEB1
RELA
TCF4
SOX12
NFATC2
SOX2
ZNF519
NFE2L2

Supplementary Figure 22. Distal regulation of gained super-enhancer-associated genes by potent TFs.

a. Hi-C maps at the *ANO1*, *HSP90AA1*, and *PMEPA1* neighboring regions within apparent TADs (highlighted in purple rectangles). The Integrative Genomics Viewer (IGV) views of ChIP-seq enrichment for H3K27ac signals in four representative LNC samples (patient No. 10, 13, 19, and 21) were shown. The TFs potentially bound to the highlighted TAD regions were predicted using HOMER and depicted below.

b. Quantitative real-time PCR analysis of *ANO1*, *HSP90AA1*, and *PMEPA1* expression upon knockdown of 12 gained enhancer-predicted TFs. n=2 biologically independent experiments presented. Source data are provided as a Source Data file.

Supplementary Table 1. DNA oligo sequences used for shRNA construction.

Oligo Name	Oligo sequence (5'-3')
shCtrl-For	GATCCGAACAAGATGAAGAGCACCAATTCAAGAGATTGGTGCTCTTCATCTTGTTTTTTTG
shCtrl-Rev	AATTCAAAAAAAACAAGATGAAGAGCACCAATCTCTTGAATTGGTGCTCTTCATCTTGTTCG
shSOX2-1-For	GATCCGAGCTCGCAGACCTACATGAATTCAAGAGATTCATGTAGGTCTGCGAGCTTTTTTG
shSOX2-1-Rev	AATTCAAAAAAAGCTCGCAGACCTACATGAATCTCTTGAATTCATGTAGGTCTGCGAGCTCG
shSOX2-2-For	GATCCGAAGAAGGATAAGTACACGCTTTCAAGAGAAGCGTGTACTTATCCTTCTTTTTTTG
shSOX2-2-Rev	AATTCAAAAAAAAGAAGGATAAGTACACGCTTCTCTTGAAAGCGTGTACTTATCCTTCTTCG
shRXRA-1-For	GATCCGGATTTCTCCACCCAGGTGAATTCAAGAGATTCACCTGGGTGGAGAAATCTTTTTTG
shRXRA-1-Rev	AATTCAAAAAAGATTTCTCCACCCAGGTGAATCTCTTGAATTCACCTGGGTGGAGAAATCCG
shRXRA-2-For	GATCCGAAGGACTGCCTGATTGACAATTCAAGAGATTGTCAATCAGGCAGTCCTTTTTTTG
shRXRA-2-Rev	AATTCAAAAAAAAGGACTGCCTGATTGACAATCTCTTGAATTGTCAATCAGGCAGTCCTTCG
shNFE2L2-1-For	GATCCGTGACAGAAGTTGACAATTATTCAAGAGATAATTGTCAACTTCTGTCATTTTTTG
shNFE2L2-1-Rev	AATTCAAAAAATGACAGAAGTTGACAATTATCTCTTGAATAATTGTCAACTTCTGTCACG
shNFE2L2-2-For	GATCCGGTAAGAAGCCAGATGTTAATTCAAGAGATTAACATCTGGCTTCTTACTTTTTTG
shNFE2L2-2-Rev	AATTCAAAAAAGTAAGAAGCCAGATGTTAATCTCTTGAATTAACATCTGGCTTCTTACCG
shZNF519-1-For	GATCCGCATTCTCAAAGCTTACTCAATTCAAGAGATTGAGTAAGCTTTGAGAATGTTTTTTG
shZNF519-1-Rev	AATTCAAAAAACATTCTCAAAGCTTACTCAATCTCTTGAATTGAGTAAGCTTTGAGAATGCG
shZNF519-2-For	GATCCGCTCCTACAGAACCATGTATTTTCAAGAGAAATACATGGTTCTGTAGGAGTTTTTTG
shZNF519-2-Rev	AATTCAAAAAACTCCTACAGAACCATGTATTTCTCTTGAAAATACATGGTTCTGTAGGAGCG
shESRRA-1-For	GATCCGAGAGGAGTATGTTCTACTAATTCAAGAGATTAGTAGAACATACTCCTCTTTTTTTG
shESRRA-1-Rev	AATTCAAAAAAAGAGGAGTATGTTCTACTAATCTCTTGAATTAGTAGAACATACTCCTCTCG
shESRRA-2-For	GATCCGTGAATGCACTGGTGTCTCATTTCAAGAGAATGAGACACCAGTGCATTCATTTTTTG
shESRRA-2-Rev	AATTCAAAAAATGAATGCACTGGTGTCTCATTCTCTTGAAATGAGACACCAGTGCATTCACG
shRELA-1-For	GATCCGCCTTAATAGTAGGGTAAGTTTTCAAGAGAAACTTACCCTACTATTAAGGTTTTTTG
shRELA-1-Rev	AATTCAAAAAACCTTAATAGTAGGGTAAGTTTCTCTTGAAAACTTACCCTACTATTAAGGCG
shRELA-2-For	GATCCGGGATTGAGGAGAAACGTAAATTCAAGAGATTTACGTTTCTCCTCAATCCTTTTTTG
shRELA-2-Rev	AATTCAAAAAAGGATTGAGGAGAAACGTAAATCTCTTGAATTTACGTTTCTCCTCAATCCCG
shTCF4-1-For	GATCCGACGAAATCTTCGGAGGACAATTCAAGAGATTGTCCTCCGAAGATTTCGTTTTTTTG
shTCF4-1-Rev	AATTCAAAAAAACGAAATCTTCGGAGGACAATCTCTTGAATTGTCCTCCGAAGATTTCGTCG
shTCF4-2-For	GATCCGAAAGGAATCTGAATCCGAAATTCAAGAGATTTCGGATTCAGATTCCTTTTTTTTG
shTCF4-2-Rev	AATTCAAAAAAAAAGGAATCTGAATCCGAAATCTCTTGAATTTCGGATTCAGATTCCTTTCG
shETS1-1-For	GATCCGTGGAATTACTCACTGATAAATTCAAGAGATTTATCAGTGAGTAATTCCATTTTTTG
shETS1-1-Rev	AATTCAAAAAATGGAATTACTCACTGATAAATCTCTTGAATTTATCAGTGAGTAATTCCACG
shETS1-2-For	GATCCGCCCTGGGTAAAGACTGCTTTTTCAAGAGAAAAGCAGTCTTTACCCAGGGTTTTTTG
shETS1-2-Rev	AATTCAAAAAACCCTGGGTAAAGACTGCTTTTCTCTTGAAAAAGCAGTCTTTACCCAGGGCG
shSOX12-1-For	GATCCGATGGCGGATTACCCGGACTATTCAAGAGATAGTCCGGGTAATCCGCCATTTTTTTG
shSOX12-1-Rev	AATTCAAAAAAATGGCGGATTACCCGGACTATCTCTTGAATAGTCCGGGTAATCCGCCATCG
shSOX12-2-For	GATCCGCTGGGCTTTCTGTCCAGGCTTTCAAGAGAAGCCTGGACAGAAAGCCCAGTTTTTTG
shSOX12-2-Rev	AATTCAAAAAACTGGGCTTTCTGTCCAGGCTTCTCTTGAAAGCCTGGACAGAAAGCCCAGCG
shNFATC2-1-For	GATCCGCACATCATGTACTGCGAGAATTCAAGAGATTCTCGCAGTACATGATGTGTTTTTTG
shNFATC2-1-Rve	AATTCAAAAAACACATCATGTACTGCGAGAATCTCTTGAATTCTCGCAGTACATGATGTGCG
shNFATC2-2-For	GATCCGCGAGTCCAAAGTTGTGTTTATTCAAGAGATAAACACAACTTTGGACTCGTTTTTTG
shNFATC2-2-Rve	AATTCAAAAAACGAGTCCAAAGTTGTGTTTATCTCTTGAATAAACACAACTTTGGACTCGCG
shIRF2-1-For	GATCCGCGGTCCTGACTTCAACTATATTCAAGAGATATAGTTGAAGTCAGGACCGTTTTTTG
shIRF2-1-Rve	AATTCAAAAAACGGTCCTGACTTCAACTATATCTCTTGAATATAGTTGAAGTCAGGACCGCG
shIRF2-2-For	GATCCGAGAACGGCCTTCTAAGAAAGTTCAAGAGACTTTCTTAGAAGGCCGTTCTTTTTTTG
shIRF2-2-Rve	AATTCAAAAAAAGAACGGCCTTCTAAGAAAGTCTCTTGAACTTTCTTAGAAGGCCGTTCTCG
shZEB1-1-For	GATCCGCTCTCTGAAAGAACACATTATTCAAGAGATAATGTGTTCTTTCAGAGAGTTTTTTG
shZEB1-1-Rve	AATTCAAAAAACTCTCTGAAAGAACACATTATCTCTTGAATAATGTGTTCTTTCAGAGAGCG
shZEB1-2-For	GATCCGCTACCACTGGATGTAGTAAATTCAAGAGATTTACTACATCCAGTGGTAGTTTTTTG
shZEB1-2-Rve	AATTCAAAAAACTACCACTGGATGTAGTAAATCTCTTGAATTTACTACATCCAGTGGTAGCG

Supplementary Table 2. Quantitative real-time PCR primer sequences.

qPCR primer name (Human)	Primer sequences (5'-to-3')
ZEB1-For	TTACACCTTTGCATACAGAACCC
ZEB1-Rve	TTTACGATTACACCCAGACTGC
GAPDH-For	GGAGCGAGATCCCTCCAAAAT
GAPDH-Rev	GGCTGTTGTCATACTTCTCATGG
SOX2-For	TGGACAGTTACGCGCACAT
SOX2-Rev	CGAGTAGGACATGCTGTAGGT
RXRA-For	ATGGACACCAAACATTTCCTGC
RXRA-Rev	GGGAGCTGATGACCGAGAAAG
NFE2L2-For	TCAGCGACGGAAAGAGTATGA
NFE2L2-Rev	CCACTGGTTTCTGACTGGATGT
ZNF519-For	ACTTCTGACCCATTCTCAA
ZNF519-Rev	CTTATGTCCCTTTAGATGTG
ESRRA-For	GAGATCACCAAGCGGAGACG
ESRRA-Rev	ATGAGACACCAGTGCATTCAC
RELA-For	ATGTGGAGATCATTGAGCAGC
RELA-Rev	CCTGGTCCTGTGTAGCCATT
TCF4-For	CAAGCACTGCCGACTACAATA
TCF4-Rev	CCAGGCTGATTCATCCCACTG
ETS1-For	GATAGTTGTGATCGCCTCACC
ETS1-Rev	GTCCTCTGAGTCGAAGCTGTC
SOX12-For	AAGAGGCCGATGAACGCATT
SOX12-Rev	TAGTCCGGGTAATCCGCCAT
NFATC2-For	GAGCCGAATGCACATAAGGTC
NFATC2-Rve	CCAGAGAGACTAGCAAGGGG
IRF2-For	AATGCTGCCCCTATCAGAACG
IRF2-Rve	CAGGACCGCATACTCAGGAGA
p21-For	TGTCCGTCAGAACCCATGC
p21-Rev	AAAGTCGAAGTTCCATCGCTC
PMEPA1-For	TGTCAGGCAACGGAATCCC
PMEPA1-Rev	CAGGTACGGATAGGTGGGC
ANO1-For	CTGATGCCGAGTGCAAGTATG
ANO1-Rev	AGGGCCTCTTGTGATGGTACA
HSP90AA1-For	GCTTGACCAATGACTGGGAAG
HS90AA1-Rev	AGCTCCTCACAGTTATCCATGA
SNAI1-For	TCGGAAGCCTAACTACAGCG
SNAI1-Rev	AGATGAGCATTGGCAGCGAG
FN1-For	CGGTGGCTGTCAGTCAAAG
FN1-Rev	AAACCTCGGCTTCCTCCATAA
C-MYC-For	GGCTCCTGGCAAAAGGTCA
C-MYC-Rev	CTGCGTAGTTGTGCTGATGT
