# Supplementary material for: Oncogenic enhancers drive esophageal squamous cell carcinogenesis and metastasis
Source: Nat Commun. 2021 Jul 22;12:4457. doi: 10.1038/s41467-021-24813-2 (PMC8298514; doi:10.1038/s41467-021-24813-2)
Supplement: Supplementary file 2 — Description of Additional Supplementary Files [file 41467_2021_24813_MOESM2_ESM.pdf]

Description of Additional Supplementary Files

Supplementary Data 1. Normalized gene expression profiles of all RNA-seq samples in TPM.

Supplementary Data 2. Identified 24823 promoter elements and 57675 enhancers elements.

Supplementary Data 3. Differential promoter elements and enhancer elements.

Supplementary Data 4. Six groups of differential enhancers.

Supplementary Data 5. Super enhancers identified from Nor, EC and LNC and their associated genes.

Supplementary Data 6. Altered super enhancers-associated DEGs.

Supplementary Data 7. Transcriptome-based DEGs.

Supplementary Data 8. Correlation analysis between H3K27ac signal and gene expression in Group 1, 2, 3, 6.

Supplementary Data 9. Biomarker expression in TPM.

Supplementary Data 10. Gene list for heatmap.

Supplementary Data 11. Differential SEs ranked by absolute difference values.

Supplementary Data 12. SEs identified from each sample and differential SEs ranked by fold change.

Supplementary Data 13. Top differential SEs and linked gene expression.

Supplementary Data 14. Clustering analysis of 456 top differential SE-linked gene expression.

Supplementary Data 15. Differential gene expression in PDAC and CA.

Supplementary Data 16. Overlapped DEGs between PDAC, CA and ESCC.

Supplementary Data 17. Normalized TF connectivity.

Supplementary Data 18. Expression of enhancer predicted TFs in TPM.

Supplementary Data 19. TF network weight between two TFs. The P value was calculated by the two-sided *t* test. Weight was calculated from the spearman correlation coefficient between the two genes in a line.

Supplementary Data 20. Key TFs and gained H3K27ac co-occupied peaks and associated genes; ENCODE dataset number.

Supplementary Data 21. Gained SE-associated gene interaction with candidate drugs.
