# Supplementary material for: Oncogenic enhancers drive esophageal squamous cell carcinogenesis and metastasis
Source: Nat Commun. 2021 Jul 22;12:4457. doi: 10.1038/s41467-021-24813-2 (PMC8298514; doi:10.1038/s41467-021-24813-2)
Supplement: Supplementary file 24 — Reporting Summary [file 41467_2021_24813_MOESM24_ESM.pdf]

nature research

Corresponding author(s): Yunbo Qiao

Last updated by author(s): May 29, 2021

Reporting Summary

Nature Research wishes to improve the reproducibility of the work that we publish. This form provides structure for consistency and transparency in reporting. For further information on Nature Research policies, see our Editorial Policies and the Editorial Policy Checklist.

Statistics

For all statistical analyses, confirm that the following items are present in the figure legend, table legend, main text, or Methods section.

n/a	Confirmed	
☐	☒	The exact sample size (*n*) for each experimental group/condition, given as a discrete number and unit of measurement
☐	☒	A statement on whether measurements were taken from distinct samples or whether the same sample was measured repeatedly
☐	☒	The statistical test(s) used AND whether they are one- or two-sided<br>*Only common tests should be described solely by name; describe more complex techniques in the Methods section.*
☐	☒	A description of all covariates tested
☐	☒	A description of any assumptions or corrections, such as tests of normality and adjustment for multiple comparisons
☐	☒	A full description of the statistical parameters including central tendency (e.g. means) or other basic estimates (e.g. regression coefficient) AND variation (e.g. standard deviation) or associated estimates of uncertainty (e.g. confidence intervals)
☐	☒	For null hypothesis testing, the test statistic (e.g. *F*, *t*, *r*) with confidence intervals, effect sizes, degrees of freedom and *P* value noted<br>*Give P values as exact values whenever suitable.*
☒	☐	For Bayesian analysis, information on the choice of priors and Markov chain Monte Carlo settings
☐	☒	For hierarchical and complex designs, identification of the appropriate level for tests and full reporting of outcomes
☒	☐	Estimates of effect sizes (e.g. Cohen's *d*, Pearson's *r*), indicating how they were calculated

Our web collection on statistics for biologists contains articles on many of the points above.

Software and code

Policy information about availability of computer code

Data collection	ChIP-Seq raw reads were mapped to the human reference genome (hg19) using Bowtie2 (version 2.3.4). RNA-seq reads were aligned to the human genome (hg19) using STAR-2.6.1.
Data analysis	RNA-seq reads were aligned to the human genome (hg19) using STAR-2.6.1 51 using the following parameters: --twopassMode Basic --sjdbOverhang 149 --outFilterMultimapNmax 20 --alignSJoverhangMin 8 --alignSJDBoverhangMin 1 --outFilterMismatchNmax 999 --outFilterMismatchNoverLmax 0.1 --alignIntronMin 20 --alignIntronMax 500000 --alignMatesGapMax 1000000 --outFilterScoreMinOverLread 0.33 --outFilterMatchNminOverLread 0.33 --outSAMstrandField intronMotif --chimSegmentMin 15 --chimJunctionOverhangMin 15 --chimOutType WithinBAM SoftClip --chimMainSegmentMultNmax 1 and other default parameters. Transcript abundances at the gene level were calculated as TPM values using RSEM 52. Transcripts with a cpm (count per million) of >1 in at least 18 samples were retained for subsequent analysis, and 16,173 genes passed this filtering criterion. The TPM values were further normalized through Z-score transformation when presented in heatmaps.<br>ChIP-Seq raw reads were mapped to the human reference genome (hg19) using Bowtie2 (version 2.3.4) 55. Quality control for the alignment BAM files was performed with SAMtools 56, enabling only uniquely mapped reads to be retained, and PCR duplicates were removed by Picard ("Picard Toolkit" 2019. Broad Institute, GitHub Repository. http://broadinstitute.github.io/picard/; Broad Institute) for subsequent analyses. Significant H3K27ac peaks were called by using MACS2 (2.1.1.20160309) with all default parameters except -p 1e-9 and -f BAMPE 57. Bigwig files were generated from bam files using deepTools 58 with the following parameters: -binSize 50 -extendReads 200 and --normalizeUsing RPKM. The signal intensities of each ChIP library were scored against the corresponding input library, respectively. The input-subtracted peak signal within a region was measured as a reads per kilobases per million (RPKM) value using bigWigAverageOverBed. The RPKM values were further normalized through Z-score transformation when presented in heatmaps.<br>Statistical analyses and plotting were carried out using GraphPad Prism 8.0.<br>The data from flow cytometer (BDFACSCalibur, USA) were analyzed using FlowJo 10.0 software (TreeStar, USA).

For manuscripts utilizing custom algorithms or software that are central to the research but not yet described in published literature, software must be made available to editors and reviewers. We strongly encourage code deposition in a community repository (e.g. GitHub). See the Nature Research guidelines for submitting code & software for further information.

Data

Policy information about availability of data

All manuscripts must include a data availability statement. This statement should provide the following information, where applicable:

- Accession codes, unique identifiers, or web links for publicly available datasets
- A list of figures that have associated raw data
- A description of any restrictions on data availability

The ChIP-seq and RNA-seq data generated in this study have been deposited in the Sequence Read Archive (SRA) database under accession code PRJNA665151 (https://www.ncbi.nlm.nih.gov/bioproject/PRJNA665151) and PRJNA665149 (https://www.ncbi.nlm.nih.gov/bioproject/PRJNA665149). Published RNA-seq datasets in pancreatic adenocarcinoma and colorectal cancer were obtained from the Gene Expression Omnibus (GEO) database "GSE71729" (https://www.ncbi.nlm.nih.gov/geo/query/acc.cgi?acc=GSE71729) and "GSE41258" (https://www.ncbi.nlm.nih.gov/geo/query/acc.cgi?acc=GSE41258), respectively. Two publicly available H3K27ac ChIP-Seq peaks in normal esophageal tissues were downloaded from the NIH Roadmap Epigenomics Project Data ("GSM906393" and "GSM1013127"). Processed Hi-C interactions in IMR90 cells was obtained from ENCODE "ENCFF307RGV". Other public transcription factors RXRA, NFE2L2, ZNF519, ESRRA, RELA, ETS1, IRF2 and ZEB1 ChIP-Seq datasets in HepG2, HEK293 and GM12878 were obtained from ENCODE ("ENCSR500WXT", "ENCSR488EES", "ENCSR754SOI", "ENCSR000EEW", "ENCSR000EAG", "ENCSR681WHQ", "ENCSR604UJV" and "ENCSR000BND"). And file names of the used ChIP-seq data for transcription factors were detailed in Supplementary Data 20. Source data are provided with this paper. The remaining data are available within the article, Supplementary Information, Supplementary Data files, or Source Data file.

Field-specific reporting

Please select the one below that is the best fit for your research. If you are not sure, read the appropriate sections before making your selection.

☒ Life sciences ☐ Behavioural & social sciences ☐ Ecological, evolutionary & environmental sciences

For a reference copy of the document with all sections, see nature.com/documents/nr-reporting-summary-flat.pdf

Life sciences study design

All studies must disclose on these points even when the disclosure is negative.

Sample size	For RNA-seq analysis, a total of 50 samples form 18 patients were examined. For ChIP-seq analysis, a total of 28 samples from 10 patients were examined. No size calculation was performed. At least 8 samples were collected for a single group, and they are enough for statistical and differential analysis. We also referred to the similar publications using samples from patients.
Data exclusions	Low transfection efficiency for individual groups (much lower than other comparing groups) were excluded in RNA-seq experiments.
Replication	Two or three replications were provided. We confirmed that all attempts at replications were useful.
Randomization	The groups are sure for the present study. Patients with primary tumor and lymph node tumors were subjected to the present study, with only one patient group. Then, the adjacent normal tissues, primary tumors and lymph node tumors were collected and allocated into three groups, without any randomization.
Blinding	Analysis group is sure for the present study. Patients were allocated into just only one group, and the samples were pairly allocated into adjacent normal tissues, primary tumors and lymph node tumors. Thus, it is not needed for blinding analysis.

Reporting for specific materials, systems and methods

We require information from authors about some types of materials, experimental systems and methods used in many studies. Here, indicate whether each material, system or method listed is relevant to your study. If you are not sure if a list item applies to your research, read the appropriate section before selecting a response.

Materials & experimental systems

n/a	Involved in the study	
☐	☒	Antibodies
☐	☒	Eukaryotic cell lines
☒	☐	Palaeontology and archaeology
☐	☒	Animals and other organisms
☐	☒	Human research participants
☒	☐	Clinical data
☒	☐	Dual use research of concern

Methods

n/a	Involved in the study	
☐	☒	ChIP-seq
☐	☒	Flow cytometry
☒	☐	MRI-based neuroimaging

Antibodies

Antibodies used	2 ug of an anti-H3K27ac antibody (Active Motif, 39133) was used for each ChIP experiment.
Validation	This antibody has been commercially confirmed and widely used in our and others' previous studies. The validation of the antibody for the species and applications has been on the manufacturer's website (https://www.activemotif.com/catalog/details/39133/histone-h3-acetyl-lys27-antibody-pab).

Eukaryotic cell lines

Policy information about cell lines

Cell line source(s)	Human HEK293T cells and TE1 cells were purchased from the Cell Bank (Shanghai Institutes for Biological Sciences, CAS, China). KYSE30 cells and KYSE150 cells were kind gifts from Dr. Zhihua Liu's lab (Cancer Hospital Chinese Academy of Medical Science), which were originally provided by Dr. Yutaka Shimada (Kyoto University, Kyoto, Japan).
Authentication	TE1 cells were authenticated by Cell Bank (Shanghai Institutes for Biological Sciences, CAS, China) using STR profiling. KYSE30 and KYSE150 cells were authenticated by Dr. Zhihua Liu's lab using STR profiling.
Mycoplasma contamination	We confirmed that there is no mycoplasma contamination for used cells.
Commonly misidentified lines (See ICLAC register)	None

Animals and other organisms

Policy information about studies involving animals; ARRIVE guidelines recommended for reporting animal research

Laboratory animals	Male BALB/c nude mice with 4-5 weeks
Wild animals	No wildtype animals were used in the present study.
Field-collected samples	Mice were maintained in specific pathogen free (SPF) facility under a 12 h dark-light cycle. No field collected samples were collected in the study.
Ethics oversight	Animal studies were conducted according to animal protocols approved by the local Ethics Committee of Shanghai Chest Hospital Affiliated to Shanghai Jiaotong University.

Note that full information on the approval of the study protocol must also be provided in the manuscript.

Human research participants

Policy information about studies involving human research participants

Population characteristics	A total of 18 ESCC patients were recruited, including three male and 15 female patients at stage III or IV. All subjects received chemotherapy or radiotherapy before surgical therapy. Detailed information for patients is provided in Supplementary Figure 1a.
Recruitment	ESCC patients were recruited from Department of Thoracic Surgery, Shanghai Chest Hospital Affiliated to Shanghai Jiaotong University, Shanghai, China. A total of 18 primary tumour samples from ESCC patients were collected from November 2017 to April 2018, and informed consent was obtained from all donors. All subjects received chemotherapy or radiotherapy before surgical therapy. These subjects were pre-diagnosed by biopsy with a high proportion of tumour cells and a squamous subtype. The tumour sample purity was confirmed by hematoxylin and eosin staining, with an estimated tumor cell content of at least 80%.
Ethics oversight	Tumour samples and clinical information were approved by the local Ethics Committee of Shanghai Chest Hospital Affiliated to Shanghai Jiaotong University, Shanghai, China

Note that full information on the approval of the study protocol must also be provided in the manuscript.

ChIP-seq

Data deposition

- [x] Confirm that both raw and final processed data have been deposited in a public database such as GEO.
- [x] Confirm that you have deposited or provided access to graph files (e.g. BED files) for the called peaks.

Data access links *May remain private before publication.*	ChIP-seq and RNA-seq data are deposited in the Sequence Read Archive (SRA) database (PRJNA665151 and PRJNA665149). The authors declare that all other data are available from the author upon request. For accession: RNA-Seq (https://www.ncbi.nlm.nih.gov/bioproject/PRJNA665151) and ChIP-Seq (https://www.ncbi.nlm.nih.gov/bioproject/PRJNA665149).

Files in database submission	Adj702-K27ac.R1.clean.fastq.gz, Adj1002-K27ac.R1.clean.fastq.gz, Adj1102-K27ac.R1.clean.fastq.gz, Adj1202-K27ac.R1.clean.fastq.gz, Adj1302-K27ac.R1.clean.fastq.gz, Adj1402-K27ac.R1.clean.fastq.gz, Adj1702-K27ac.R1.clean.fastq.gz, Adj1902-K27ac.R1.clean.fastq.gz, Adj2002-K27ac.R1.clean.fastq.gz, Adj2102-K27ac.R1.clean.fastq.gz, Adj17-Input.R1.clean.fastq.gz, Adj19-Input.R1.clean.fastq.gz, Adj20-Input.R1.clean.fastq.gz, Adj21-Input.R1.clean.fastq.gz, CAN701-K27ac.R1.clean.fastq.gz, CAN1001-K27ac.R1.clean.fastq.gz, CAN1101-K27ac.R1.clean.fastq.gz, CAN1201-K27ac.R1.clean.fastq.gz, CAN1301-K27ac.R1.clean.fastq.gz, CAN1401-K27ac.R1.clean.fastq.gz, CAN1701-K27ac.R1.clean.fastq.gz, CAN1901-K27ac.R1.clean.fastq.gz, CAN2001-K27ac.R1.clean.fastq.gz, CAN2101-K27ac.R1.clean.fastq.gz, CAN17-Input.R1.clean.fastq.gz, CAN19-Input.R1.clean.fastq.gz, CAN20-Input.R1.clean.fastq.gz CAN21-Input.R1.clean.fastq.gz, LNC1003-K27ac.R1.clean.fastq.gz, LNC1103-K27ac.R1.clean.fastq.gz, LNC1203-K27ac.R1.clean.fastq.gz, LNC1303-K27ac.R1.clean.fastq.gz, LNC1403-K27ac.R1.clean.fastq.gz, LNC1903-K27ac.R1.clean.fastq.gz, LNC2003-K27ac.R1.clean.fastq.gz, LNC2103-K27ac.R1.clean.fastq.gz, LNC19-Input.R1.clean.fastq.gz, LNC20-Input.R1.clean.fastq.gz, LNC21-Input.R1.clean.fastq.gz, Adj702-K27ac.R2.clean.fastq.gz, Adj1002-K27ac.R2.clean.fastq.gz, Adj1102-K27ac.R2.clean.fastq.gz, Adj1202-K27ac.R2.clean.fastq.gz, Adj1302-K27ac.R2.clean.fastq.gz, Adj1402-K27ac.R2.clean.fastq.gz, Adj1702-K27ac.R2.clean.fastq.gz, Adj1902-K27ac.R2.clean.fastq.gz, Adj2002-K27ac.R2.clean.fastq.gz, Adj2102-K27ac.R2.clean.fastq.gz, Adj17-Input.R2.clean.fastq.gz, Adj19-Input.R2.clean.fastq.gz, Adj20-Input.R2.clean.fastq.gz, Adj21-Input.R2.clean.fastq.gz, CAN701-K27ac.R2.clean.fastq.gz, CAN1001-K27ac.R2.clean.fastq.gz, CAN1101-K27ac.R2.clean.fastq.gz, CAN1201-K27ac.R2.clean.fastq.gz, CAN1301-K27ac.R2.clean.fastq.gz, CAN1401-K27ac.R2.clean.fastq.gz, CAN1701-K27ac.R2.clean.fastq.gz, CAN1901-K27ac.R2.clean.fastq.gz, CAN2001-K27ac.R2.clean.fastq.gz, CAN2101-K27ac.R2.clean.fastq.gz, CAN17-Input.R2.clean.fastq.gz, CAN19-Input.R2.clean.fastq.gz, CAN20-Input.R2.clean.fastq.gz, CAN21-Input.R2.clean.fastq.gz, LNC1003-K27ac.R2.clean.fastq.gz, LNC1103-K27ac.R2.clean.fastq.gz, LNC1203-K27ac.R2.clean.fastq.gz, LNC1303-K27ac.R2.clean.fastq.gz, LNC1403-K27ac.R2.clean.fastq.gz, LNC1903-K27ac.R2.clean.fastq.gz, LNC2003-K27ac.R2.clean.fastq.gz, LNC2103-K27ac.R2.clean.fastq.gz, LNC19-Input.R2.clean.fastq.gz, LNC20-Input.R2.clean.fastq.gz, LNC21-Input.R2.clean.fastq.gz
Genome browser session (e.g. UCSC)	Not applicable

Methodology

Replicates	28 samples from 10 patients were collected.
Sequencing depth	Each library was sequenced to an average of 30 million raw reads on 10X sequencing platform. Sequencing was performed using the 150 bp pair-end read platform. Quality control analysis showed that the Q30 score of the sequencing reads was ~90% and that the mapping ratio to the human genome (hg19) was over 75%.
Antibodies	Anti-H3K27ac antibody (Active Motif, 39133) was used for ChIP-seq analysis.
Peak calling parameters	Significant H3K27ac peaks were called by using MACS2 (2.1.1.20160309) with all default parameters except -p 1e-9 and -f BAMPE.
Data quality	Quality control for the alignment BAM files was performed with SAMtools, enabling only uniquely mapped reads to be retained, and PCR duplicates were removed by Picard ("Picard Toolkit" 2019) for subsequent analyses. Significant H3K27ac peaks were called by using MACS2 (2.1.1.20160309) with the parameters -p 1e-9.
Software	ChIP-Seq raw reads were mapped to the human reference genome (hg19) using Bowtie2 (version 2.3.4). Quality control for the alignment BAM files was performed with SAMtools, enabling only uniquely mapped reads to be retained, and PCR duplicates were removed by Picard ("Picard Toolkit" 2019. Broad Institute, GitHub Repository. http://broadinstitute.github.io/picard/; Broad Institute) for subsequent analyses. Significant H3K27ac peaks were called by using MACS2 (2.1.1.20160309) with all default parameters except -p 1e-9 and -f BAMPE. Bigwig files were generated from bam files using deepTools with the following parameters: -binSize 50 -extendReads 200 and --normalizeUsing RPKM. The signal intensities of each ChIP library were scored against the corresponding input library, respectively. The input-subtracted peak signal within a region was measured as a reads per kilobases per million (RPKM) value using bigWigAverageOverBed. The RPKM values were further normalized through Z-score transformation when presented in heatmaps.

Flow Cytometry

Plots

Confirm that:

- [x] The axis labels state the marker and fluorochrome used (e.g. CD4-FITC).
- [x] The axis scales are clearly visible. Include numbers along axes only for bottom left plot of group (a 'group' is an analysis of identical markers).
- [x] All plots are contour plots with outliers or pseudocolor plots.
- [x] A numerical value for number of cells or percentage (with statistics) is provided.

Methodology

Sample preparation	TE1 cells were plated in 24-well plates at 50,000 cells per well and were treated with the indicated chemical drugs or DMSO after attachment. Cell apoptosis was detected using a Cell Cycle and Apoptosis Detection Kit (C1052; Beyotime Institute of Biotechnology, China). After cells were treated for three days, they were trypsinized, centrifuged, washed, and fixed with cold 70% ethanol at 4 °C overnight. Then, the fixation solution was removed, and the cells were incubated at 37 °C for 30 min in a

	solution containing propidium iodide and RNase A.
Instrument	FACScan flow cytometer (BDFACSCalibur, USA)
Software	The data were analyzed using FlowJo 10.0 software (TreeStar, USA).
Cell population abundance	10,000 cells were collected per sample
Gating strategy	Standard gating for apoptotic cells were performed. All collected cells were subjected for first gating with FSC-A and SSC-A. The cellular debris and impurities were gating out; FSC-W and SSC-A were used for the second gating to exclude agminated cells. Lastly, the PE and SSC-A were used for histogram analysis, and the cells locating the area before G1-phase population were defined as apoptotic cells.

☒ Tick this box to confirm that a figure exemplifying the gating strategy is provided in the Supplementary Information.
